# A century of exercise physiology: key concepts on coupling respiratory oxygen flow to muscle energy demand during exercise

**DOI:** 10.1007/s00421-022-04901-x

**Published:** 2022-02-26

**Authors:** Guido Ferretti, Nazzareno Fagoni, Anna Taboni, Giovanni Vinetti, Pietro Enrico di Prampero

**Affiliations:** 1 Dipartimento di Medicina Molecolare e Traslazionale, Università di Brescia, Brescia, Italy; 2 Département d’Anesthésiologie, Pharmacologie et Soins Intensifs, Université de Genève, Genève, Switzerland; 3 Professore Emerito di Fisiologia, Università di Udine, Udine, Italy

**Keywords:** Oxygen flow, Oxygen consumption, Ventilation, Diffusion, Cardiac output, Haemoglobin, Metabolism, Energetics, Exercise transient, Breath-holding

## Abstract

After a short historical account, and a discussion of Hill and Meyerhof’s theory of the energetics of muscular exercise, we analyse steady-state rest and exercise as the condition wherein coupling of respiration to metabolism is most perfect. The quantitative relationships show that the homeostatic equilibrium, centred around arterial pH of 7.4 and arterial carbon dioxide partial pressure of 40 mmHg, is attained when the ratio of alveolar ventilation to carbon dioxide flow (${\dot{V}}_{A}/{\dot{V}}_{R}{CO}_{2}$) is − 21.6. Several combinations, exploited during exercise, of pertinent respiratory variables are compatible with this equilibrium, allowing adjustment of oxygen flow to oxygen demand without its alteration. During exercise transients, the balance is broken, but the coupling of respiration to metabolism is preserved when, as during moderate exercise, the respiratory system responds faster than the metabolic pathways. At higher exercise intensities, early blood lactate accumulation suggests that the coupling of respiration to metabolism is transiently broken, to be re-established when, at steady state, blood lactate stabilizes at higher levels than resting. In the severe exercise domain, coupling cannot be re-established, so that anaerobic lactic metabolism also contributes to sustain energy demand, lactate concentration goes up and arterial pH falls continuously. The ${\dot{V}}_{A}/{\dot{V}}_{R}{CO}_{2}$ decreases below − 21.6, because of ensuing hyperventilation, while lactate keeps being accumulated, so that exercise is rapidly interrupted. The most extreme rupture of the homeostatic equilibrium occurs during breath-holding, because oxygen flow from ambient air to mitochondria is interrupted. No coupling at all is possible between respiration and metabolism in this case.

## Introduction

The celebration of a century of exercise physiology, since the Nobel Prize award to Archibald Vivian Hill (1886–1977) and Otto Fritz Meyerhof (1884–1951) in 1922, is a very ambitious enterprise. In affording it, one has to look back at the impressive developments that took place since then. A comparison of what exercise physiology was in Hill and Meyerhof’s days with what it has become nowadays makes people suddenly aware of the astonishingly huge size of the scientific developments occurring in that time lapse. All of a sudden, one realises that, apart from classical exercise physiology, the number of brand new fields of research related to exercise is great indeed, from muscle and exercise molecular biology to biomechanics, from training physiology, to exercise neuroscience and exercise pathophysiology. The modern term exercise science, unknown in Hill and Meyerhof’s times, encompasses a wide spectrum of scientific activities related to exercise.

The development of exercise physiology proceeded in parallel with a tremendous societal change. In 1922, most of leisure or sport exercise practise was a privilege of a small number of rich men from aristocracy or high bourgeoisie. The romantic ideas that led to the revival of the Olympic Games inspired them. In 2022, sport has become a widespread popular practice, supporting professional activities of many people, and generating rich and profitable related industrial activities, producing and distributing sport shoes, sportswear, technical tools, training ergometers, ergogenic products, therapeutic tools, and so on. Indeed, the development of exercise physiology, and, more generally speaking, exercise science, proceeded together with, and partly induced, the impressive, enormous societal, industrial, medical, sport evolution that took place, since Hill and Meyerhof established the first basis of “modern” exercise physiology.

We are honoured to contribute to the present celebration of one century of exercise physiology by taking the heavy task of this review on our shoulders. The aim is to discuss and revise from a contemporary holistic perspective, but in the light of historical developments, a very complex concept indeed, that of coupling energy demand by the exercising muscles to energy delivery by the respiratory system. The latter is defined in its broadest sense, as the entire pathway from ambient air to mitochondria. Gas flow along this pathway is sustained by lung ventilation, lung diffusion and perfusion, cardiovascular blood flow and gas transport, peripheral diffusion and perfusion. The functional state of the respiratory system is modulated in such a way as to maintain as much as possible an equilibrium ensuring adequate oxygen delivery to support cell metabolism (at exercise mostly muscle fibres).

The concept of steady state is strictly related to the concept of coupling. Steady state, another intellectual creation of the 1920s’ physiology, implies that, whenever the rate of energy metabolism is kept invariant at the level that is necessary to support ATP resynthesis and ATPase activities, whether at rest or at exercise, also the oxygen and carbon dioxide flows, and the physiological variables that set them along the entire respiratory system, remain stable in time. The concepts of coupling and steady state stemmed from and are part of the wider concept of a stable milieu intérieur, which Claude Bernard (1813–1878) created amidst the nineteenth century (Bernard 1859, 1865). This concept implies that the normal physiological state of a living body is ensured by a strict chemico-physical equilibrium, which is maintained by fine regulatory mechanisms throughout the entire life span. Of course, the quantitative relationships at steady state ensuring respiratory–metabolic coupling must respect this equilibrium.

The structure of this review will thus be as follows. After a short account of the main findings up to the end of the nineteenth century, we discuss the revolution represented by Hill and Meyerhof’s theory of the energetics of muscular exercise and its refutation. Then, we introduce the concept of steady state and we analyse and discuss the quantitative relationships at steady state, which define the laws by which the coupling of respiration and metabolism occur. Finally, a few examples of rupture of these equilibria in unsteady state conditions are analysed, namely the exercise transient, including severe exercise, and breath-holding.

## Antiquity to the nineteenth century

Classical Greek medicine recognized the muscles as the site of movement, but did not connect them to other functions of the body. A major step forward occurred when dissection was permitted in Alexandria. Therefore, in the early third century BC, Herophilus (≈ 330–250 BC) not only recognized the importance of muscles for movement, but also distinguished between motor nerves and sensitive nerves, reported the connection of the former to muscles, proposed a role for motor nerves in muscle contraction, and eventually established what is very likely the first theory of contraction. Galen (129–201 AD) summarized Herophilus’ theory as follows: “during contraction the muscles are filled with pneuma, increase in breadth, but diminish in length”. Here, skeletal muscles are clearly defined as the organs of voluntary movement; if muscles are filled with *pneuma*, perhaps through the nerves, which according to Herophilus contain *pneuma*, they are contracted, id est active, they shorten and generate movement; at the end of movement, they lose *pneuma* and relax in a passive manner (von Staden 1989).

These ideas remained substantially unchanged until the Renaissance. The great natural philosophers of that era (sixteenth–seventeenth century) viewed muscles pretty much as the Alexandrian School, although they downgraded the metaphysical concept of *pneuma* as the origin of contraction. René Descartes (1596–1650) replaced it with the “vital spirits”, the behaviour of which should be subjected to physical laws.

The cultural climate underwent drastic changes in that time. Focus moved from a metaphysical to a physical vision of nature, which led to the birth of modern experimental science. This was a real cultural revolution, inasmuch as scientific theory was enslaved to experimental validation, or falsification. The revolutionary spirit of those days, equalled only, although on a smaller scale, by the cultural climate in early Alexandria, gave origin to classical Galilean and Newtonian physics, classical chemistry, new mathematical tools—analytical geometry and calculus were then created—together with new specific measurement tools. Among these, the invention of the microscope allowed substantial advances also in the field of muscle physiology: Giovanni Alfonso Borelli (1608–1679), William Croone (1633–1684), Anton van Leeuwenhoek (1632–1723), Niels Steensen (1638–1686), and Jan Swammerdam (1637–1680) established by observation and experiments the fibrous structure of muscle, its cross-striation, and demonstrated that muscle contraction may occur without changes in volume (Needham 1971).

In that time, it became clear that air did not consist of a single gas but was a mixture of different gaseous constituents. Michal Sedziwój (1566–1636) had already proposed that one constituent of air may be a “life-giving” substance. In fact, when kalium nitrate was heated, a gas was liberated that could be collected in flasks and used. According to the Dutch engineer Cornelis Drebbel (1566–1625), that gas could sustain up to 12 men in a submarine rowing longer than 1 h from Westminster to Greenwich down the river Thames (Poole et al. 2015). That substance may well correspond to the “nitro-aerial” particles of air, which John Mayow (1643–1679) suggested to be used during contraction, with the simultaneous elimination of a body constituent. This was a totally new concept, which, for the first time in history, suggested a relation of muscle contraction to respiration and a yet vague idea of coupling (Mayow 1681). This suggestion is perhaps the first seed that later grew into the current idea of oxygen utilisation in metabolism associated with carbon dioxide elimination, as long as that gas was probably oxygen.

The ideas of Mayow gained a prominent place in the history of physiology, thanks to the subsequent developments in the chemistry of gases, one of the most exciting scientific adventures of seventeenth and eighteenth centuries. The concept of gas, as that of air, preceded the scientific revolution of seventeenth century. In fact, already Anaximenes of Miletus (≈ 586–526 BC) regarded air, which he called *pneuma*, as the initial substance of the physical world, although he thought air to be a single, pure gas. The modern physics of air started with Robert Boyle (1627–1691), who described the inverse relationship between pressure and volume in a given quantity of gas. Since the product of pressure times volume is an amount of energy, Boyle’s relationship indicated that the amount of energy in a given quantity of gas was a constant.

It took more than one century, with the creation of the concept of temperature and the invention of the thermometer and of the temperature scales, to demonstrate that Boyle’s constant varied linearly with temperature. At the end of a long path, Emile Clapeyron (1799–1864) defined, in 1834, what is usually known as the equation of state of ideal gases, and expressed it in this ﻿form

1a $$E = PV = nR\left( {T - T_{0} } \right),$$

where *E*, *P*, *V*, and *T* stand for energy, pressure, volume, and temperature (expressed in °C), *n* represents the amount of gas, currently expressed in moles, and *R*, which was called the universal constant of ideal gases, indicates the amount of energy introduced into 1 mol of gas by a unit increase in temperature. In fact, *R* is the angular coefficient of the linear relationship between the energy in a given gas quantity and the temperature of the same gas, for *n* = 1. Constant *T*_0_ is the intercept on the *x*-axis of the same relationship and corresponds to the temperature at which *E* = 0 J. It is equal to − 273.14 °C and was called absolute zero. When William Thomson, first Baron Kelvin (1824–1907) set *T*_0_ equal to zero—in fact, he shifted the *x*-axis intercept of the *E* versus *T* relationship rightward to have it coinciding with the origin of the axes—, he created the absolute temperature scale, named in his honour. Therefore, if we express temperature in absolute scale (Kelvin degrees, °K), Eq. (﻿1a) becomes

1b $$E = PV = nRT,$$

thereby setting a relation of direct proportionality between *E* and *T*. Equation (﻿1b) had numerous remarkable consequences, among which we underline (i) the definition of heat as a form of energy, (ii) the opening of the path that led to the creation of the new concept of entropy, and, in the present niche context, (iii) the formulation of theories of the energetics of muscular exercise. Concerning respiration, Eq. (﻿1b) defined the criteria of quantitative standardization for gas volumes and flows, which were established by the American Physiological Society (Pappenheimer 1950). The conventional expression of air volume and flow in BTPS (body temperature and pressure, saturated with water vapour) and of single gas volume and flow in STPD (standard temperature and pressure, dry) dates back to that time.

A fundamental step forward, providing a chemical basis to the aforementioned assertion of John Mayow, occurred in the second half of the eighteenth century. Joseph Black (1728–1799) discovered carbon dioxide in 1764, which he called “fixed air”, and which he reported to be exhaled during respiration. Joseph Priestley (1733–1804) and Carl Wilhelm Scheele (1742–1786) discovered oxygen independently in the 1770s. Priestley, who first published his experiment in 1774, called it “dephlogisticated air”, which means air without phlogiston, which was then thought to be the (metaphysical) fire-like element liberated during combustion processes. In fact, the theory of phlogiston was deeply rooted in eighteenth century chemistry. Georg Ernst Stahl (1660–1734) was a strong advocate of the phlogiston theory. According to him, inflammation (combustion) liberated phlogiston, so that the burned substance was transformed into ashes. Shortly after the identification of oxygen and carbon dioxide, Antoine-Laurent Lavoisier (1743–1794) refuted the phlogiston theory, demonstrating that combustion implies the combination of a fuel with oxygen (theory of oxidation). He also realised that animals, and *inter eos* humans, consume Priestley’s gas, which he called oxygen, and demonstrated that the rate of oxygen consumption ($\dot{V}{O}_{2}$) increased when a man exercised. Moreover, together with Armand Séguin (1767–1835), he showed that exercise was associated also with an increased elimination of carbon dioxide and an increased production of heat (Séguin and Lavoisier 1789). Combination of these observations led to the notion that the production of mechanical energy in muscle contraction is a chemical process of combustion, in which oxygen reacts with a fuel, yielding carbon dioxide as the end-product, and heat is generated in the process. This meant that exercise was possible thanks to chemical energy transformations in mechanical work and heat, although heat was not yet clearly seen as a form of energy. This was a revolutionary concept indeed, so fraught with consequences for future physiological developments, that we can easily recognize it as the seminal starting point of exercise physiology, without which Hill and Meyerhof’s theories, which we celebrate, would not have been possible. In May 1794, during the most radical period of the French Revolution, Lavoisier was condemned to death, allegedly for his previous support to the aristocratic regime, and the guillotine put an end, together with his life, to his revolutionary scientific work.

A necessary consequence of the work by Séguin and Lavoisier was that the process of chemical energy transformation requires oxidation of an organic fuel, which was then unknown. Moreover, a tight match between oxygen consumption and fuel oxidation must be in place, as well as between respiration and muscle energy transformations, since oxygen must be taken from ambient air. Lavoisier believed that combustion occurred in the lungs, and that the generated heat was removed by blood circulation. Adair Crawford (1748–1795) proposed a different theory of animal heat, which we summarize as follows: the oxygen contained in ambient air is converted into carbon dioxide in the lungs, thereby liberating heat; this heat, however, does not increase lung temperature, because of differences in the specific heat of arterial and venous blood. As a consequence, the blood in the pulmonary vein undergoes an increase in specific heat due to heat delivery from the lung. Hence, Crawford resurrected the phlogistic theory, stating that combustibles consisted of ash combined with a fire principle, the “phlogiston”, which was liberated during burning. Crawford believed that phlogiston prompted the alleged heat delivery to blood, despite that Lavoisier had already demonstrated the incorrectness of the phlogiston theory. This is a nice example indeed of how difficult it is to dismiss a theory that was proven untrue, when it has become a dogma.

For a more detailed report of the history of classical gas chemistry, we are pleased to direct the readers to the magnificent chapter of the first edition of the Handbook of Physiology, on the history of respiration (Perkins 1964). That chapter discusses, inter alia, the discovery of nitrogen, the definition of the composition of ambient air, the creation of Dalton’s law and of the concept of partial pressure of pure gases in a gas mixture, and the formulation of Henry’s law, describing the principles that govern the solution of gases in a liquid (plasma, as far as we are concerned). Although all these concepts pertain to the present article, we refrain from discussing them here for reasons of space.

The identification of the fuel supporting the combustion process generating the mechanical energy for organismal movement progressed from the chemical experiments on aliments of the first half of the nineteenth century. Justus von Liebig (1803–1873) and Michel Eugène Chevreul (1786–1889) described carbohydrates and fatty acids, respectively. This was the starting point of a dramatic scientific process that, within a century, led to the definition of the main biochemical pathways of intermediate metabolism. These include glycolysis (Meyerhof 1921, 1924), the Krebs’ cycle (Krebs and Kornberg 1957), the beta-oxidation of fatty acids (Beinert 1963), and the oxidative phosphorylation in the electron transport chain (Mitchell 1979). Incidentally, Otto Fritz Meyerhof (glycolysis), Hans Krebs (1900–1981) (Krebs’ cycle), and Peter Mitchell (1920–1992) (oxidative phosphorylation) were awarded the Nobel Prize for Physiology or Medicine in 1922, 1953, and 1978, respectively.

We also note that there was no discovery of metabolic pathways made by an isolated genius who unveiled the unknown. Meyerhof, Krebs, Beinert, and Mitchell synthesized in comprehensive theories complex metabolic processes, the development of which lasted decades and integrated the work of many scientists from several laboratories, who defined the single steps and described the various chemical components of each pathway. The ensemble of these pathways set the biochemical basis of oxidative metabolism, and provided an exhaustive explanation of the links between substrates (fuel) and oxygen, and thus between $\dot{V}{O}_{2}$ and rate of carbon dioxide production ($\dot{V}{CO}_{2}$). Those who are interested in the historical details of that epopee can refer to a bunch of reviews on these topics (see e.g. Ghisla 2004; Kornberg 2000; Krebs 1970; Mitchell 2004, 2011; Racker 1983).

By the second half of the nineteenth century, the physiological community was strongly convinced that the behaviour of all living things, muscles included, was amenable to physical and chemical laws. In this cultural climate, Hermann von Helmholtz (1821–1894) was the first who showed that the law of conservation of energy can be applied to living organisms (Helmoltz 1847), a hypothesis later supported more in detail by Danilewski (1880) and by Rubner (1894) in animals, and then by Atwater (1904) in man. The hypothesis that muscle is tantamount to a heat engine was supported by Mayer (1845), and later by Engelmann (1895). In contrast, Adolf Fick (1829–1901) rejected it, since the observed efficiency of human and horse muscles (20–25%), as he rightly pointed out, would require temperature gradients physiologically unacceptable, on a heat engine hypothesis (Fick 1893).

Meanwhile, structural studies on muscles were carried out in what we can now define as the golden age of optical microscopy. Ranvier (1873) recognized the existence of red and white muscles with different morphological and physiological characteristics. Engelmann (1878) carefully described striation of muscle. Kölliker (1888) provided convincing evidence of the existence of myofibrils. At the dawn of the twentieth century, numerous studies on the energetics of muscular exercise in man had been carried out, along the path opened by the pioneering work of Lavoisier. The resulting conclusions by several authors (Chauveau and Kaufmann 1887; Chauveau and Tissot 1896; Heidenhain 1864; Heinemann 1901; Kölliker 1888; Pettenkofer and Voigt 1866; Zuntz 1901) on the fuel of choice, the energy expenditure, and the structure of muscle are closer to the currently accepted ones than we could have imagined, and represent the solid foundation for the revolution engendered by Meyerhof and Hill.

## A turning point: Hill and Meyerhof’s theory

After Fletcher and Hopkins (1907) had demonstrated that lactic acid, an end-product of glycolysis, accumulates in contracting muscles, Hill and Meyerhof, who shared the 1922 Nobel Prize for Physiology or Medicine, condensed the remarkable evidence collected by Fletcher and Hopkins (1907, 1917), and by the two of them separately (Hill 1913, 1916, 1922; Meyerhof 1920, 1921, 1922), in the first modern theory of the energetics of muscular contraction, currently incorporated under the term “Hill and Meyerhof’s theory”.

According to this theory, the primary energy source for muscle contraction is the oxidation of glycogen to lactic acid through glycolysis, regardless of the availability of oxygen. $\dot{V}{O}_{2}$ intervenes only during recovery, when glycogen is resynthesized. Meyerhof (1922, 1924) assumed that during recovery, id est in aerobic conditions, about a quarter of the lactate produced during contraction is oxidized, thus yielding the energy for the resynthesis of the remaining three-quarters to glycogen. An analysis of the time course of the $\dot{V}{O}_{2}$ at the onset of, during, and in the recovery after moderate exercise, prompted Hill et al. (1924) to introduce the term “oxygen debt”. By this, they defined the amount of oxygen utilised in the recovery after exercise for the resynthesis to glycogen of part of the lactic acid accumulated in the contracting muscles during the preceding exercise period.

When Hill and Meyerhof formulated their theory of the energetics of muscle contraction, the existence of lactic acid had been known for more than a century. Carl Wilhelm Scheele isolated it from sour milk as impure brown syrup and it was recognized as an important chemical constituent of living organisms. Johannes Wislicenus (1835–1902) established its structure in 1873 (Wislicenus 1873). Then, Andersson (1933) described the structure of the enzyme catalysing the reduction of pyruvate to lactate, which he called lactate dehydrogenase.

Hill and Meyerhof’s theory did not establish a clear distinction between aerobic and anaerobic metabolism during exercise. They considered the energy balance during muscle contraction an essentially anaerobic process, whereas the energy balance during recovery appeared as an aerobic process, for $\dot{V}{O}_{2}$ was necessary for glycogen synthesis. The cycle was closed by the combination of both processes.

However, as time went by, the continuous evolution of biochemical knowledge provided numerous data that did not fit in the above picture:

- Embden and Lawaczeck (1922) and Stella (1928) showed that the concentration of inorganic phosphate (Pi) in muscles increases during a series of contractions.
- Eggleton and Eggleton (1927a, b), Fiske and Subbarow (1927, 1928), and Nachmanson (1928) identified phosphocreatine (PCr) and showed that its concentration in muscle decreases during contraction.
- Lohmann (1928) isolated a new type of organic phosphate, adenosine-tri-phosphate (ATP), and showed that it is also present in muscle.
- Lundsgaard (1930a, b) demonstrated that anoxic muscles poisoned with iodoacetic acid, which prevents lactate formation, can contract repeatedly, albeit for a limited time, without lactic acid accumulation.

Hill realised that the discovery of phosphates could undermine his theory and, as a scientist, accepted the risk of refutation. Therefore, he wrote an astonishing review (Hill 1932), wherein, from a “loser” perspective, he predicted a “revolution” in muscle physiology and tried to defend his theory by integrating the phosphates in it. However, contrary to the expectations of Hill’s review, a masterpiece of humility, and intellectual honesty, it was not phosphate that killed the Hill and Meyerhof’s theory. Phosphates could in principle be accommodated in it, by simply taking them as the link between the biochemical pathways and the contractile unit in muscle fibres. In fact, Rodolfo Margaria (1901–1983) actioned the guillotine and his concept of the alactic oxygen debt hammered the nail in the coffin of Hill and Meyerhof’s theory.

## Margaria’s refutation of Hill and Meyerhof’s theory

Margaria et al. (1933) showed that, over a large range of running speeds in humans, blood lactate concentration does not increase appreciably above resting, thus indicating that no lactate production, as they said, or, more correctly, no lactate accumulation, as we would say nowadays, has occurred during exercise. Hence, under those experimental conditions, the oxygen debt paid in the recovery period could not be attributed to lactate removal. Therefore, it was defined “alactic” oxygen debt and attributed to the resynthesis of the amount of “phosphagen” split at the onset of exercise. This finding effectively refuted Hill and Meyerhof’s theory.

The study by Margaria et al. (1933) is the cornerstone of the theory that, after 90 years, is still considered to provide a substantially correct view of the energetics of muscle contraction and muscular exercise: the hydrolysis of phosphagen as the primary mechanism yielding the necessary amount of energy supporting muscle contraction and mechanical power generation. Optimally, and this is indeed the case during the exercise steady state, the hydrolysis of phosphagen and its resynthesis at the expense of oxygen consumption must proceed at the same rate. Conversely, at the beginning of exercise, because of the inertia of metabolic processes activation, a fraction of the phosphagen split during muscle contraction cannot be resynthesized; it will be only during recovery after exercise, at the expense of the “alactic” oxygen debt.

It is noteworthy that, when Margaria et al. (1933) published their study, ATP and PCr were not yet functionally separated, but were still lumped together under the term “phosphagen”. The functional separation of ATP and PCr occurred one year later, when Lohmann (1934) proposed ATP to be the key molecule, necessary for muscular contraction. His view was better focused, and thus reinforced, in 1939, when Engelhardt and Lyubimova (1939) discovered the ATPase activity of myosin. Their discovery bridged the gap between structure and function, so that the energy liberation from ATP splitting could be accommodated in the subsequent sliding filament theory of muscle contraction (see e.g. Huxley 1957, 1974).

The refutation of Hill and Meyerhof’s theory by Margaria et al. (1933) and the subsequent creation of the concept of the alactic oxygen debt gave origin to a huge amount of work on the energetics of muscular exercise in humans. To briefly summarize the most fundamental concepts, pertinent to this review, Margaria first wrote of different, yet concomitant metabolic pathways for ATP resynthesis during exercise, namely aerobic metabolism, sustaining moderate intensity exercise for long periods of time, the epiphenomenon of which is $\dot{V}{O}_{2}$, and anaerobic metabolism, sustaining explosive exercise for short periods of time. Later, to include the resynthesis of ATP from PCr hydrolysis in the picture, he splitted the concept of anaerobic metabolism in two: anaerobic lactic metabolism, represented by the rate of lactate accumulation in blood, and anaerobic alactic metabolism, implying ATP resynthesis from PCr without lactate accumulation. The latter accounts for explosive efforts of few seconds duration. In his view (Margaria 1968), each metabolism was characterised by a maximal capacity—maximal amount of energy that it can provide—and by a maximal power. Maximal oxygen consumption was identified as the maximal power attained by aerobic metabolism. He established the concept of an energy equivalent of blood lactate accumulation (Margaria et al. 1963), and used it to define the power at which the maximal rate of blood lactate accumulation (in fact, he wrote production…) is attained as the maximal lactic power (Margaria et al. 1964).

Margaria was convinced, and rightly enough in our view, that there must be a relationship of direct proportionality between the epiphenomenal measurable variables characterising each metabolism [$\dot{V}{O}_{2}$ for aerobic metabolism, rate of increase in blood lactate concentration ($\dot{La}$) for anaerobic lactic metabolism, and rate of decrease in muscle PCr concentration ($\dot{PCr}$) for anaerobic alactic metabolism], and the rate of energy delivery (metabolic power, $\dot{E}$). Thus, each metabolism can indeed be defined by its maximal power and capacity and by its energy equivalent (di Prampero 1981; di Prampero and Ferretti 1999; Ferretti 2015).

Margaria’s energetic vision of exercise physiology led to the analysis of the exercise transient in terms of oxygen deficit, which we now consider as consisting of an obligatory (former Margaria’s alactic oxygen debt) and a facultative (“early lactate” accumulation and changes in oxygen stores) component (di Prampero 1981). The latter intervenes only when the former is unable, for any reason, to ensure the energy balance of the oxygen deficit. The alactic oxygen deficit is a physiological necessity, as long as a reduction of muscle PCr concentration determines the activation of the glycolytic pathway, and thus the acceleration of the entire oxidative pathways leading to the increase of $\dot{V}{O}_{2}$. Thus, PCr, and its counterpart, free creatine, are key molecules for coupling ATP resynthesis to ATP consumption during muscular contraction (di Prampero and Margaria 1968; di Prampero et al. 2003; Mader 2003; Mahler 1985). These concepts will be better detailed in Sect. “The Energetics of the Oxygen Deficit”.

All developments subsequent to the work of Margaria et al. (1933), especially within the physiological school that he initiated, had the remarkable property of being fully compatible with the general principles of thermodynamics. The successors of Margaria in Milano kept this concept very clear in their mind, and the experiments that they conceived to test Margaria’s energetic theory were driven by a thermodynamic vision. The energetics of human locomotion has also been treated within the boundaries of thermodynamic compatibility. The energetics of muscular exercise and of human locomotion has been analysed and discussed in numerous reviews and books (Åstrand et al. 2003; Brooks 2000, 2012; di Prampero 1981, 1986, 2000, 2015; di Prampero and Ferretti 1999; Ferguson et al. 2018; Ferretti 2014, 2015; Grassi 2000, 2003; Jones and Poole 2005; Lacour and Bourdin 2015; Poole and Jones 2012; Poole et al. 2016, 2021; Taylor and Heglund 1982; Whipp and Ward 1982, 1990; Zamparo et al. 2020), which the readers are referred to for more detailed analysis.

**Fig. 1:**
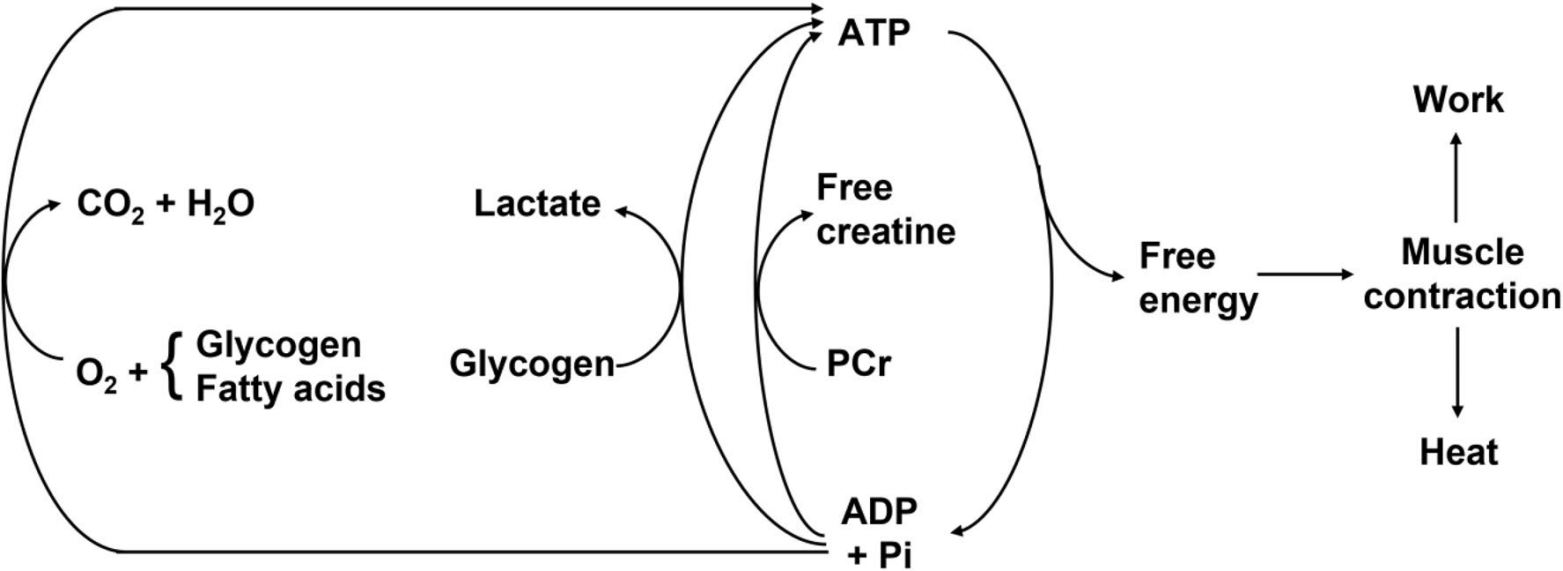
Schematic representation of the energetics of muscular exercise. Adenosine triphosphate (ATP) hydrolysis provides the free energy that contracting muscles use to produce work and heat. ATP resynthesis is ensured by the indicated pathways. ADP, adenosine-di-phosphate, Pi, inorganic phosphate, PCr, phosphocreatine [Modified after di Prampero (1981)]

The large volume of data collected and of theoretical analyses formulated after the paper by Margaria et al. (1933) have led to the creation of a general theory of the energetics of muscular exercise, which we mostly owe to the physiological school that Rodolfo Margaria established in Milano after World War II. The first formulation of this theory dates back to 1968 (di Prampero and Margaria 1968); its first comprehensive summary was published in 1981 (di Prampero 1981). This general theory, summarized in Fig. 1, can be condensed in algebraic form as follows (di Prampero 1981; Ferretti 2015):

2 $$\dot{E}\propto \overleftarrow{\dot{ATP}}=\overrightarrow{\dot{ATP}}=c\dot{V}{O}_{2}+b\dot{La}+a\dot{PCr},$$

where the metabolic power $\dot{E}$ represents the overall rate of metabolic energy liberation, $\overleftarrow{\dot{ATP}}$ and $\overrightarrow{\dot{ATP}}$ are the rates of ATP hydrolysis and resynthesis, respectively, $\dot{La}$ is the rate of lactate accumulation in blood, and $\dot{PCr}$ is the rate of PCr drop in the working muscle fibres. The three constants *a*, *b*, and *c* are proportionality constants indicating the moles of ATP resynthesized, respectively, by a mole of hydrolysed PCr, a mole of lactate accumulated, and a mole of oxygen consumed. The Lohmann’s reaction tells that *a* is equal to 1; *c* corresponds to the *P*/*O*_*2*_ ratio and is equal to 6.17 for complete glycogen oxidation into glycolysis, Krebs cycle, and oxidative phosphorylation. For constant *b*, corresponding to the *P*/*La* ratio (moles of phosphate released per mole of lactate formed by glycolysis), the situation is a bit more complex (see di Prampero 1981; di Prampero and Ferretti 1999; Ferretti 2015). Notwithstanding, the data collected so far indicate a mean value for *b* ranging between 1.25 and 1.05 mol of phosphate per mole of lactate accumulated, which is slightly less than the value of 1.5 predicted from stoichiometry.

As shown in Fig. 1, the general theory of the energetics of muscular exercise considers muscle as a biological engine, in which chemical energy is transformed into mechanical work and heat. The ultimate step of this energy transformation is provided by ATP hydrolysis in the cross-bridge cycle. The low ATP concentration in muscle (on average 5 mmol per kg of wet muscle) requires continuous ATP resynthesis, which we owe to the ensemble of reactions comprised in the concept of intermediate metabolism and synthetized in Eq. (﻿2). Three basic metabolic pathways for ATP resynthesis appear, each corresponding to a precise physiological concept: aerobic metabolism, anaerobic lactic metabolism, and anaerobic alactic metabolism. Aerobic metabolism includes the oxidation of glycogen and fatty acids to pyruvate and acetate, respectively, and their introduction into the Krebs’ cycle, which feeds oxidative phosphorylation. Anaerobic lactic metabolism includes the degradation of glycogen to pyruvate, with subsequent reduction of pyruvate to lactate by the action of lactate dehydrogenase. It occurs when the rate of energy transformation in glycolysis exceeds that of oxidation of pyruvate in the Krebs’ cycle. Anaerobic alactic metabolism includes resynthesis of ATP through the Lohmann reaction and hydrolysis of immediately available ATP.

$\dot{La}$ and $\dot{PCr}$ are intrinsic muscular phenomena, which are fully separated from the respiratory system. Of course $\dot{La}$ = 0 mmol s^−1^ if lactate production equals lactate removal by muscle fibres and by other organs. The same is the case for $\dot{PCr}$, if PCr remains constant, as occurs when aerobic metabolism can resynthesize ATP at a rate sufficient to cope with ATP hydrolysis during muscle contraction. This condition is certainly attained in humans at rest and during moderate exercise, when $\dot{V}{O}_{2}$ reaches a steady level, which is maintained for a long time. Under these steady-state conditions, there is neither accumulation of lactate in blood nor changes in muscle PCr concentration, so that the terms $b \dot{La}$ and $a \dot{PC}$ of Eq. (﻿2) are nil, the metabolism is purely aerobic, and Eq. (﻿2) reduces to

3 $$\dot{E}\propto \overleftarrow{\dot{ATP}}=\overrightarrow{\dot{ATP}}=c \dot{V}{O}_{2}.$$

When Eq. (﻿3) applies, all the metabolic power sustaining body functions at rest, plus the mechanical work for exercise, derives from aerobic energy sources, $\dot{V}{O}_{2}$ is proportional to $\dot{E}$, and ATP resynthesis through the overall biochemical pathway of aerobic metabolism equals ATP consumption. In a steady-state condition, $\dot{E}$ and thus $\dot{V}{O}_{2}$ stay invariant in time.

The tight matching of $\dot{V}{O}_{2}$ with $\dot{E}$, however, can be maintained only if there is a tight matching between $\dot{V}{O}_{2}$ and the oxygen flow along the respiratory system (${\dot{V}}_{R}{O}_{2}$), inasmuch as the $\dot{V}{O}_{2}$ can be sustained only by taking oxygen from ambient air and conveying it to cells (mostly muscle fibres at exercise) along the respiratory system. In other terms, cellular respiration must be strictly coupled to ${\dot{V}}_{R}{O}_{2}$, which occurs if, and only if

4 $$\dot{V}{O}_{2}={\dot{V}}_{R}{O}_{2}.$$

In the next paragraphs, we discuss how the conditions summarized by Eq. (4﻿) are attained and maintained in the body. We first introduce the steady state concept and its relation to the respiratory system, and describe the quantitative relationships that characterise it. Then, we discuss how the equilibrium is maintained when $\dot{V}{O}_{2}$ increases, as at exercise, we analyse the cardiopulmonary responses to exercise at steady state, and synthetize the interrelated phenomena along the respiratory system by which a steady $\dot{V}{O}_{2}$ is maintained during exercise.

## The steady-state concept

Bock et al. (1928) defined the steady-state concept for the first time. According to them, a human is in steady-state condition when: $\dot{V}{O}_{2}$ and $\dot{V}{CO}_{2}$ are invariant, the carbon dioxide eliminated through the mouth is produced only by metabolism, there is a steady heart rate (${f}_{H}$), and the milieu intérieur is essentially stable. This definition of steady state implies that

1. Equation (﻿4) holds at steady state.
2. ${\dot{V}}_{R}{O}_{2}$ takes the same value at any level along the respiratory system, i.e., in the alveoli, across the systemic circulation and across the alveolar–capillary barrier.
3. The outflow of carbon dioxide (${\dot{V}}_{R}{CO}_{2}$) is the same at any level along the respiratory system, i.e., across the systemic circulation, across the alveolar–capillary barrier and in the alveoli, and is equal to $\dot{V}{CO}_{2}$.

If the preceding points are correct, it necessarily follows that the respiratory quotient determined at the lungs (*RQ*_*L*_)—or gas exchange ratio, defined as the ratio between ${\dot{V}}_{R}{CO}_{2}$ and ${\dot{V}}_{R}{O}_{2}$—is equal to the respiratory quotient determined at any other level along the respiratory system. Consequently, *RQ*_*L*_ is also equal to the metabolic respiratory quotient (*RQ*_*M*_), id est the ratio between the moles of carbon dioxide produced and the moles of oxygen consumed at the cellular level.

Taking the above three points as axioms, several developments in the analysis of the quantitative relationships describing gas flows in the respiratory system at rest and exercise have been pursued. Before entering into these details, however, it is necessary to point out that the steady-state concept is a mental creation of some brilliant physiologists, yet represents an oversimplification of real life. This type of steady state has been reasonably approximated in the laboratory, for instance at rest and during moderate exercise at constant power. Notwithstanding, even at steady state, the respiratory system shows oxygen flow discontinuities, heterogeneities and spontaneous variations, depending on the level of organisation, macroscopic or microscopic, at which we operate.

At the macroscopic level, pulmonary ventilation occurs in a *cul de sac*, so that, contrary to what happens in birds’ parabronchi, air must enter and quit the lungs through a common path, consisting of the airways. Such a structural arrangement implies that (i) air inhalation (inspiration) and exhalation (expiration) occur necessarily in alternate manner: no continuous steady air flow is possible in such a system, and (ii) a fraction of the inhaled air cannot attain the alveoli, but remains trapped in the airways. This fraction cannot participate in alveolar gas exchange, and thus, its volume is called the dead space volume (Bohr 1891; Krogh and Lindhard 1913a, 1917). Moreover, the heart alternates systoles and diastoles, so that pressure varies continuously inside the ventricles and in the aorta. The opening and closing of heart valves occur synchronous to pressure variations. Therefore, blood flow in the heart and in the aorta is necessarily discontinuous. Discontinuities in ventilation and in total blood flow carry along oscillations both in oxygen and in carbon dioxide flows. We have then to consider that there is a spontaneous variability of respiratory and cardiac rhythms, related to mechanical and neural control mechanisms (Cottin et al. 2008; Perini and Veicsteinas 2003). This being the case, ${\dot{V}}_{R}{O}_{2}$ and ${\dot{V}}_{R}{CO}_{2}$ at steady state are not continuous invariant flows: the steady-state ${\dot{V}}_{R}{O}_{2}$ and ${\dot{V}}_{R}{CO}_{2}$ are invariant integral means of flows that are highly variable in time, at several levels even discontinuous.

At the microscopic level, blood flow is pulsatile in lung capillaries, because of their heterogeneous recruitment in space and time, and because of the effects of the rhythmic activity of the heart and the lungs on lung capillary pressure (Baumgartner et al. 2003; Clark et al. 2011; Heinonen et al. 2013; Roy and Secomb 2019; Tanabe et al. 1998). Heterogeneity of lung capillary blood flow may be less at exercise, due to simultaneous recruitment of a larger number of lung capillaries to sustain the higher pulmonary blood flow.

Similar heterogeneities in capillary blood flow were demonstrated also in contracting skeletal muscles, both in space and in time (Armstrong et al. 1987; Ellis et al. 1994; Heinonen et al. 2007; Marconi et al. 1988; Piiper et al. 1985). Heterogeneous distribution of muscle blood flow was found also in non-contracting muscles of exercising humans (Heinonen et al. 2012). Contracting muscle fibres are likely unperfused, because they generate pressure, which compresses and closes muscle capillaries from outside as a Starling resistor. If this is so, only relaxed muscle fibres would be perfused, so that muscle fibre oxygenation takes place during relaxation, not during contraction. In this case, the alternate recruitment of neighbouring motor units is a physiological necessity, entailing heterogeneity of muscle blood flow distribution during exercise (Cano et al. 2015; Goulding et al. 2021). Nevertheless, capillaries themselves are tethered to adjacent myocytes by collagenous struts, which, when pulled, exert a force acting in the opposite direction (Abovsky et al. 1996; Borg and Caulfield 1980; Caulfield et al. 1985). In both skeletal and cardiac muscle capillaries, this force helps keep capillaries open. However, during intense contractions, capillary flow may cease when arterioles and venules collapse. This means that, at least during short contractions of the surrounding fibres, red blood cells, which are retained in the closed capillaries, may keep exchanging gases.

Finally, we note that, on a longer time scale, also *RQ*_*M*_ can change, if substrate utilisation shifts between glycogen and fatty acids as energy sources. This occurs, for instance, during very long light exercise, when glycogen stores are close to exhaustion, so that fatty acid oxidation represents a progressively larger fraction of the overall aerobic metabolism (Fernandez-Verdejo et al. 2018; Galgani et al. 2008, 2012).

## Quantitative relationships at steady state

### Of two mass balance equations

Keeping the three axioms listed in the previous section in mind, let us start from a definition of ${\dot{V}}_{R}{O}_{2}$ and ${\dot{V}}_{R}{CO}_{2}$ as a difference between a flow in and a flow out for the gas at stake, providing the gas flow exchanged in the lung alveoli and in peripheral capillaries.^1^ This definition implies that ${\dot{V}}_{R}{O}_{2}$ and ${\dot{V}}_{R}{CO}_{2}$ can be obtained by mass balance equations. Fick (1870) described the first equations of this type, which summarize what we call in his honour the Fick principle, and which are expressed in the following form, respectively for oxygen and carbon dioxide:

5a $${\dot{V}}_{R}{O}_{2}=\dot{Q}\; {C}_{a}{O}_{2}-\dot{Q}\; {C}_{\overline{v}}{O}_{2}=\dot{Q} \left({C}_{a}{O}_{2}- {C}_{\overline{v}}{O}_{2}\right),$$

5b $${\dot{V}}_{R}{CO}_{2}=\dot{Q}\; {C}_{a}{CO}_{2}-\dot{Q} \; {C}_{\overline{v}}{CO}_{2}=\dot{Q} \left({C}_{a}{CO}_{2}-{C}_{\overline{v}}{CO}_{2}\right),$$

where $\dot{Q}$ is total blood flow, or more commonly cardiac output, ${C}_{a}{O}_{2}$ and ${C}_{\stackrel{-}{v}}{O}_{2}$, and ${C}_{a}{CO}_{2}$ and ${C}_{\stackrel{-}{v}}{CO}_{2}$ are the oxygen and carbon dioxide concentrations in arterial and mixed venous blood, respectively. In fact, Eqs. (﻿5a)^2^ and (﻿5b) define, respectively, the volume of oxygen that leaves the blood in peripheral capillaries to be consumed in cells and the volume of carbon dioxide produced by metabolism that leaves the cells to be removed through the respiratory system, per unit of time.

Along the same line, Geppert and Zuntz (1888) defined ${\dot{V}}_{R}{O}_{2}$ as the difference between inspired and expired oxygen flows, as follows:

6a $${\dot{V}}_{R}{O}_{2}{=\dot{V}}_{I}\;{F}_{I}{O}_{2}-{\dot{V}}_{E}\;{F}_{E}{O}_{2},$$

6b $${\dot{V}}_{R}{CO}_{2}={\dot{V}}_{I}\;{F}_{I}{CO}_{2}-{\dot{V}_{E}\;{F}_{E}{CO}}_{2},$$

where ${\dot{V}}_{I}$ is the total inspired air flow, or inspiratory ventilation, ${\dot{V}}_{E}$ is the total expired air flow, or expiratory ventilation, ${F}_{I}{O}_{2}$ and ${F}_{E}{O}_{2}$ are the oxygen fractions in inspired and expired air, respectively, and ${F}_{I}{CO}_{2}$ and ${F}_{E}{CO}_{2}$ are the carbon dioxide fractions in inspired and expired air, respectively. Further developments around Eq. (﻿6a), accounting for the fact that *RQ*_*L*_ = *RQ*_*M*_ is less than 1, and thus, ${\dot{V}}_{E}<{\dot{V}}_{I}$, in a variety of steady-state conditions, can be found in Otis (1964) and led to the following equation for the calculation of ${\dot{V}}_{R}{O}_{2}$, when ${\dot{V}}_{I}$ cannot be measured:

7 $${\dot{V}}_{R}{O}_{2}{=\dot{V}}_{E} \left[\frac{(1-{F}_{E}{O}_{2}-{F}_{E}{CO}_{2}\text{)}}{{F}_{I}{N}_{2}} {F}_{I}{O}_{2}-{F}_{E}{O}_{2}\right],$$

where ${F}_{I}{N}_{2}$ is the nitrogen fraction in inspired air.

### Maintaining the equilibrium in the lungs

Since in respiration physiology, ${F}_{I}{CO}_{2}$ is generally considered to be negligible (in fact, average ${F}_{I}{CO}_{2}$ = 0.000415 in dry air), the first term of the right-hand branch of Eq. (﻿6b) can be omitted. Moreover, at steady state, ${\dot{V}}_{R}{CO}_{2}$ is equal in expired as in alveolar air. Thus, in steady-state condition, Eq. (6b) can be rewritten as follows:

8 $${\dot{V}}_{R}{CO}_{2}={-\dot{V}}_{E}\; {F}_{E}{CO}_{2} \, ={-\dot{V}}_{A}\; {F}_{A}{CO}_{2}{=-\dot{V}}_{A} \frac{{P}_{A}{CO}_{2}}{{P}_{B}-47},$$

where ${\dot{V}}_{A}$ is alveolar ventilation, ${P}_{A}{CO}_{2}$ is the partial pressure of carbon dioxide in alveolar air, *P*_*B*_ is the barometric pressure, and 47 mmHg is the water pressure in the alveoli, assuming alveolar air to be at 37 °C (310 °K) and saturated with water. Equation (﻿8) tells that at any given *P*_*B*_ and ${\dot{V}}_{R}{CO}_{2}\text{,}$ ${\dot{V}}_{A}$ is inversely proportional to ${P}_{A}{CO}_{2}$, whereas at any given ${P}_{A}{CO}_{2}$, it is directly proportional to $-{\dot{V}}_{R}{CO}_{2}\text{.}$ The linear relationship between ${\dot{V}}_{A}$ and ${-\dot{V}}_{R}{CO}_{2}$ in “standard” steady-state acid–base conditions (pH = 7.40, arterial carbon dioxide partial pressure, ${P}_{a}{CO}_{2}=40 \, \mathrm{mmHg}$; bicarbonate concentration = 25 mEq L^−1^) lies on the ${P}_{A}{CO}_{2}$ isopleth for 40 mmHg (${P}_{A}{CO}_{2}={P}_{a}{CO}_{2}$), which implies a ${\dot{V}}_{A}$/${\dot{V}}_{R}{CO}_{2}$ equal to − 21.6.^3^ At exercise, the increase in ${\dot{V}}_{A}$ is proportional to that in $-{\dot{V}}_{R}{CO}_{2}$, a condition that has been called hyperpnoea: the acid base conditions are kept invariant and equal to the resting ones.

This is so as long as there is no continuous blood lactate accumulation ($b \dot{La}=$ 0 W), and thus, Eq. (﻿3) applies. This is the case at rest, as well as during moderate aerobic exercise (Ferretti 2015; Poole et al. 2016). A continuous blood lactate accumulation occurs and affects pH regulation at powers above the critical power (Jones et al. 2008). In top-level marathon runners, this occurs at powers higher than 90% of the maximal aerobic power (Tam et al. 2012).

Above these intensities, although the exercise is still submaximal, lactate keeps increasing in blood, the term $b \dot{La}$ of Eq. (﻿2) becomes positive and pH is not sufficiently buffered. Hyperventilation occurs, and is superimposed to hyperpnoea, and the ${\dot{V}}_{A}$ versus $-{\dot{V}}_{R}{CO}_{2}$ relationship bends upward, toward isopleths for lower ${P}_{A}{CO}_{2}$ and more negative ${\dot{V}}_{A}/{\dot{V}}_{R}{CO}_{2}$. Moreover, the so-called slow component of the ${\dot{V}}_{R}{O}_{2}$ kinetics appears, so that ${\dot{V}}_{R}{O}_{2}$ keeps increasing with time (Ferretti 2015; Poole and Jones 2012; Poole et al. 1988, 1994). Steady-state conditions are disrupted, as is the tight matching between $\dot{V}{{O}}_{2}$ and ${\dot{V}}_{R}{O}_{2}$. Nevertheless, even in the severe exercise domain (above the critical power), type I muscle fibres can still consume the oxygen that the respiratory system is able to deliver to them. Concerning type II fibres, neither nuclear magnetic resonance (Richardson et al. 2015) nor cryomicrospectroscopy (Honig et al. 1984) provided any evidence that oxygen delivery is limited in the severe exercise domain: thus, the metabolic limit of these fibres may be set by their ability to consume oxygen.

If Eq. (﻿6a) is rewritten in terms of ${\dot{V}}_{A}$, by subtracting dead space ventilation, and of partial pressures, by applying Dalton’s law, and we divide the result by Eq. (﻿8), we can compute *RQ*_*L*_ as follows:

9 $${RQ}_{L} = \frac{{ - P_{A} {{CO}}_{2} }}{{\left( {P_{I} {{O}}_{2} - P_{A} {{O}}_{2} } \right)}},$$

where ${P}_{I}{O}_{2}$ and ${P}_{A}{O}_{2}$ are the oxygen partial pressures in inspired and alveolar air, respectively. The solution of Eq. (﻿9) for ${P}_{A}{O}_{2}$ and ${P}_{A}{CO}_{2}$ yields

10 $${P}_{A}{O}_{2} = {P}_{I}{O}_{2} - \frac{-{P}_{A}{CO}_{2}}{{RQ}_{L}},$$

11 $${P}_{A}{CO}_{2}={RQ}_{L} \left( {P}_{A}{O}_{2}-{P}_{I}{O}_{2}\right).$$

**Fig. 2:**
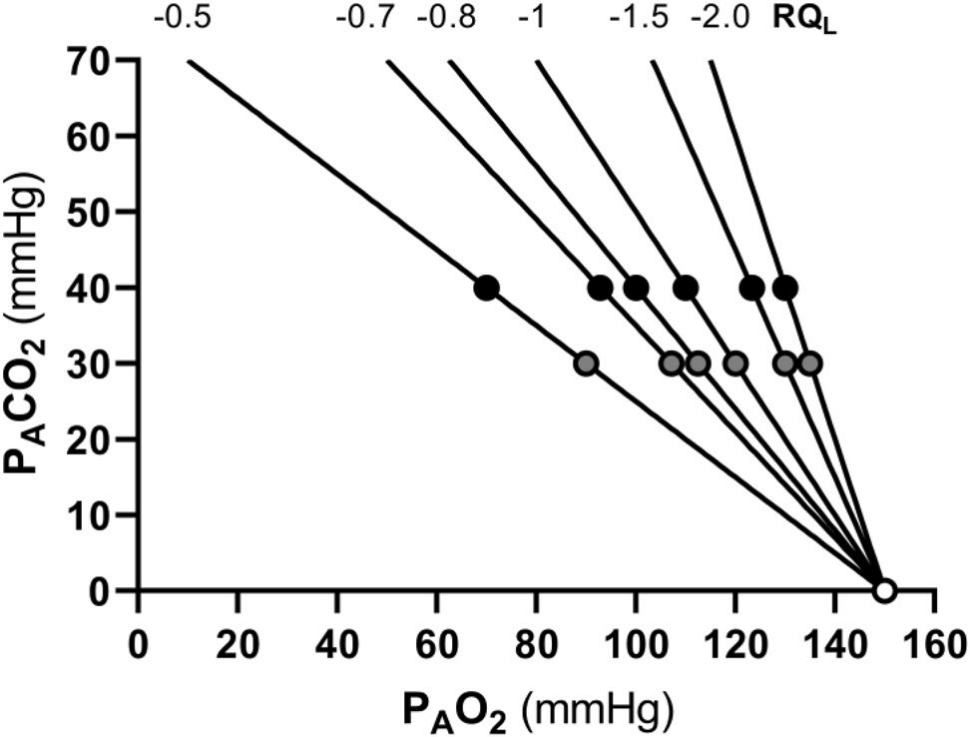
O_2_–CO_2_ diagram for alveolar air, wherein alveolar partial pressure of carbon dioxide (${P}_{A}{CO}_{2}$) is plotted as a function of the alveolar partial pressure of oxygen (${P}_{A}{O}_{2}$). Six isopleths of pulmonary respiratory quotient (*RQ*_*L*_) are shown, converging on the *x*-axis at a value corresponding to the inspired partial pressure of oxygen at sea level (white dot), thus yielding the gas composition of ambient air. On each line, the points indicating alveolar air composition (black dots) and expired air composition (grey dots) at each *RQ*_*L*_ are reported. Note that, for alveolar air, ${P}_{A}{CO}_{2}$ is always at 40 mmHg (regulated variable), independent of *RQ*_*L*_. This necessarily implies that, the closer to zero is the *RQ*_*L*_, the lower is the ${P}_{A}{O}_{2}$ [Modified after Ferretti et al. (2017)]

Equations (﻿10) and (﻿11) represent two expressions of the so-called alveolar air equation (Fenn et al. 1946). Equation (﻿11) tells that, if in steady-state condition, we plot ${P}_{A}{CO}_{2}$ as a function of ${P}_{A}{O}_{2}$ on a *x*–*y* diagram, we obtain a linear relationship, the slope of which is equal to *RQ*_*L*_ (thus, it is negative), and which has an intercept on the *x*-axis equal to ${P}_{I}{O}_{2}$, thus providing the composition of ambient inspired air (${P}_{I}{CO}_{2}=$ 0 mmHg). This graphical representation is the oxygen–carbon dioxide diagram (O_2_–CO_2_ diagram) for alveolar gases (Rahn and Fenn 1955) (Fig. 2), A family of isopleths for *RQ*_*L*_, all converging on a given inspired air composition (e.g., ${P}_{I}{O}_{2}$ = 150 mmHg at sea level), are reported on the same figure.

It is a corollary of the steady-state axioms that, in fact, the only possible alveolar air compositions lie on the *RQ*_*L*_ isopleth corresponding to the actual *RQ*_*M*_. *RQ*_*M*_ ranges between − 0.8 at rest and − 1.0 around the lactate threshold, the difference depending on the increasing fraction of energy derived from carbohydrate oxidation, as the exercise intensity, and thus the $\dot{V}{O}_{2}$, go up. Within this range of metabolic powers, the maintenance of the homeostatic equilibrium described above ensures tight coupling of respiration and metabolism. The invariance of ${P}_{A}{CO}_{2}$ around the value of 40 mmHg is ensured at the expense of ${P}_{A}{O}_{2}$, the value of which at steady state necessarily varies depending on *RQ*_*L*_. These ${P}_{A}{O}_{2}$ variations are predictable from Eq. (﻿10).

**Fig. 3:**
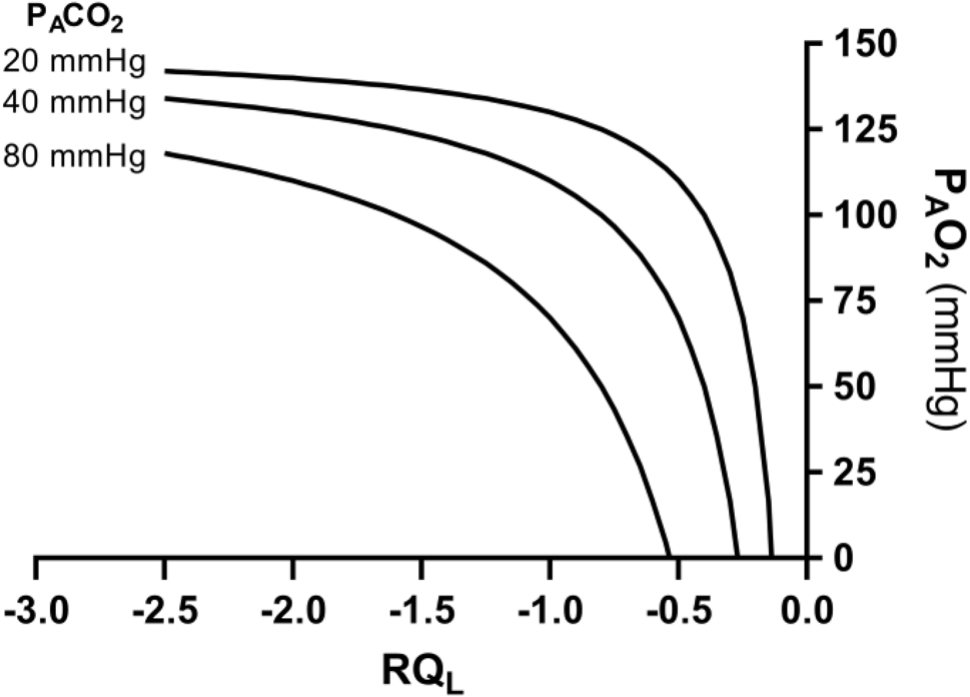
A representation of three hyperbolic functions describing the relationship between the alveolar partial pressure of oxygen (${P}_{A}{O}_{2}$) and the pulmonary respiratory quotient (*RQ*_*L*_). The reported curves hold for an inspired partial pressure of oxygen of 150 mmHg (horizontal asymptote) and for the indicated alveolar partial pressures of carbon dioxide, expressed in mmHg (curvature)

Equation (﻿11) tells also that, if we plot ${P}_{A}{O}_{2}$ as a function of *RQ*_*L*_, the relationship is described by a translated hyperbola, with a negative curvature (downward convexity) equal to $-{P}_{A}{CO}_{2}$, and a horizontal asymptote equal to ${P}_{I}{O}_{2}$. The relationship between ${P}_{A}{O}_{2}$ and *RQ*_*L*_ for a ${\dot{V}}_{A} /{\dot{V}}_{R}{CO}_{2}$ of − 21.6 (${P}_{A}{CO}_{2}=$ 40 mmHg) at sea level (${P}_{I}{O}_{2}=$ 150 mmHg) is shown in Fig. 3. Obviously enough, a family of isopleths for ${\dot{V}}_{A} /{\dot{V}}_{R}{CO}_{2}=$ − 21.6 can be constructed, displaced downward if ${P}_{I}{O}_{2}$ decreases (hypoxia), upward if ${P}_{I}{O}_{2}$ increases (hyperoxia). Otherwise, for any given ${P}_{I}{O}_{2}$, when the ${\dot{V}}_{A} /{\dot{V}}_{R}{CO}_{2}$ decreases (becomes more negative) and the ${P}_{A}{CO}_{2}$ decreases (hyperventilation), the curve becomes more convex; conversely, when the ${\dot{V}}_{A} /{\dot{V}}_{R}{CO}_{2}$ goes up and the ${P}_{A}{CO}_{2}$ increases (hypoventilation), the curve becomes less convex.

Variations of ${P}_{I}{O}_{2}$ in hypoxia and in hyperoxia have an impact also on the O_2_–CO_2_ diagram, in so far as the inspired air point is shifted leftward and rightward, respectively. Since the *RQ*_*L*_ isopleths converge on the inspired air point, the entire family of these isopleths is displaced accordingly. Notwithstanding, as long as there is no hyperventilation due to hypoxaemic stimulation of peripheral chemoreceptors, the ${\dot{V}}_{A} /{\dot{V}}_{R}{CO}_{2}$ remains invariant at − 21.6, and thus, ${P}_{A}{CO}_{2}$ stays equal to 40 mmHg.^4^ Conversely, when the hyperventilation induced by hypoxaemia intervenes, which is accentuated at a ${P}_{a}{O}_{2}$ lower than some 60 mmHg, the ${\dot{V}}_{A} /{\dot{V}}_{R}{CO}_{2}$ and ${P}_{A}{CO}_{2}$ decrease. For any given *RQ*_*L*_, the alveolar gas composition is displaced downward and rightward along the corresponding *RQ*_*L*_ isopleth, in the direction of the inspired air point. The ${P}_{A}{O}_{2}$ increases by an amount that is predictable from the alveolar air equation, based on the incurring ${P}_{A}{CO}_{2}$ and *RQ*_*L*_.

**Fig. 4:**
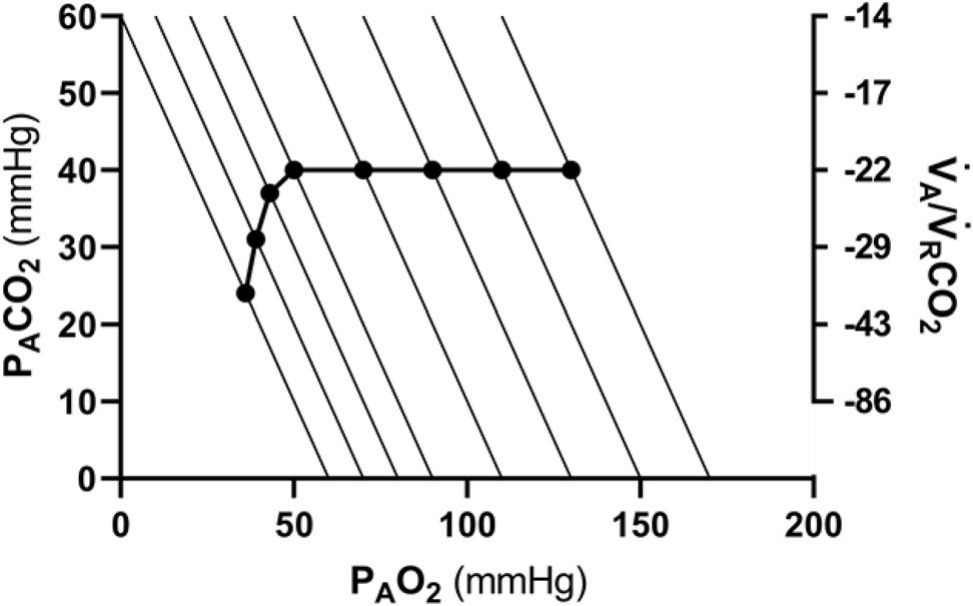
On the O_2_–CO_2_ diagram for alveolar air, the thin parallel lines are isopleths for a lung respiratory quotient of − 1.0, which intercept the *x*-axis at different inspired air points. As the inspired partial pressure of oxygen (${P}_{I}{O}_{2}$) decreases, the isopleth is displaced to the left. The black dots correspond to the incurring alveolar gas composition at each ${P}_{I}{O}_{2}$. The line connecting all these points (thick black curve) is the alveolar air curve. ${P}_{A}{CO}_{2}$ and ${P}_{A}{O}_{2}$ are the alveolar partial pressures of carbon dioxide and oxygen, respectively. ${\dot{V}}_{A} /{\dot{V}}_{R}{CO}_{2}$ alveolar ventilation to lung carbon dioxide flow ratio [Modified after Ferretti (2015)]

If for each ${P}_{I}{O}_{2}$ value, and thus each *RQ*_*L*_ isopleth, we connect all the points representing the steady-state alveolar air composition, we obtain a curve like that reported in Fig. 4, which Rahn and Fenn (1955) called the normal mean alveolar air curve. If no hypoxaemia occurs, this curve is parallel to the *x*-axis, indicating that ${P}_{A}{CO}_{2}$ is independent of ${P}_{I}{O}_{2}$ in that segment of the curve, as long as ${\dot{V}}_{A} /{\dot{V}}_{R}{CO}_{2}$ = − 21.6, and thus, the “standard” steady-state acid–base conditions are preserved. As the ${P}_{I}{O}_{2}$ becomes sufficiently low to determine a stimulation of the peripheral chemoreceptors, and thus hyperventilation, the ${\dot{V}}_{A} /{\dot{V}}_{R}{CO}_{2}$ decreases and the curve bends downward, so that ${P}_{A}{CO}_{2}$ becomes lower, the stronger is hyperventilation, and the lower becomes the ${\dot{V}}_{A} /{\dot{V}}_{R}{{CO}}_{2}$.

### Maintaining the equilibrium in blood: the effect of ventilation–perfusion heterogeneity

The O_2_–CO_2_ diagram was established for alveolar air. Yet, a similar diagram can be constructed for blood, making use of the Fick principle. In fact, we can obtain the respiratory quotient for blood (${RQ}_{B}$) by dividing Eqs. (﻿5a) and (﻿5b)

12 $${RQ}_{B}=\frac{{\dot{V}}_{R}{CO}_{2}}{{\dot{V}}_{R}{O}_{2}}=\frac{\dot{Q} ({C}_{a}{CO}_{2}-{C}_{\overline{v}}{CO}_{2})}{\dot{Q} ({C}_{a}{O}_{2}-{C}_{\overline{v}}{O}_{2})}= \frac{{C}_{a}{CO}_{2}-{C}_{\overline{v}}{CO}_{2}}{{C}_{a}{O}_{2}-{C}_{\overline{v}}{O}_{2}}.$$

Of course, at steady state, *RQ*_*B*_ = *RQ*_*L*_ = *RQ*_*M*_. Solving Eq. (﻿12) for ${C}_{a}{CO}_{2}$, we obtain

13 $${C}_{a}{CO}_{2}= {C}_{\overline{v}}{CO}_{2}+{RQ}_{B}\left({C}_{a}{O}_{2}-{C}_{\overline{v}}{O}_{2}\right).$$

**Fig. 5:**
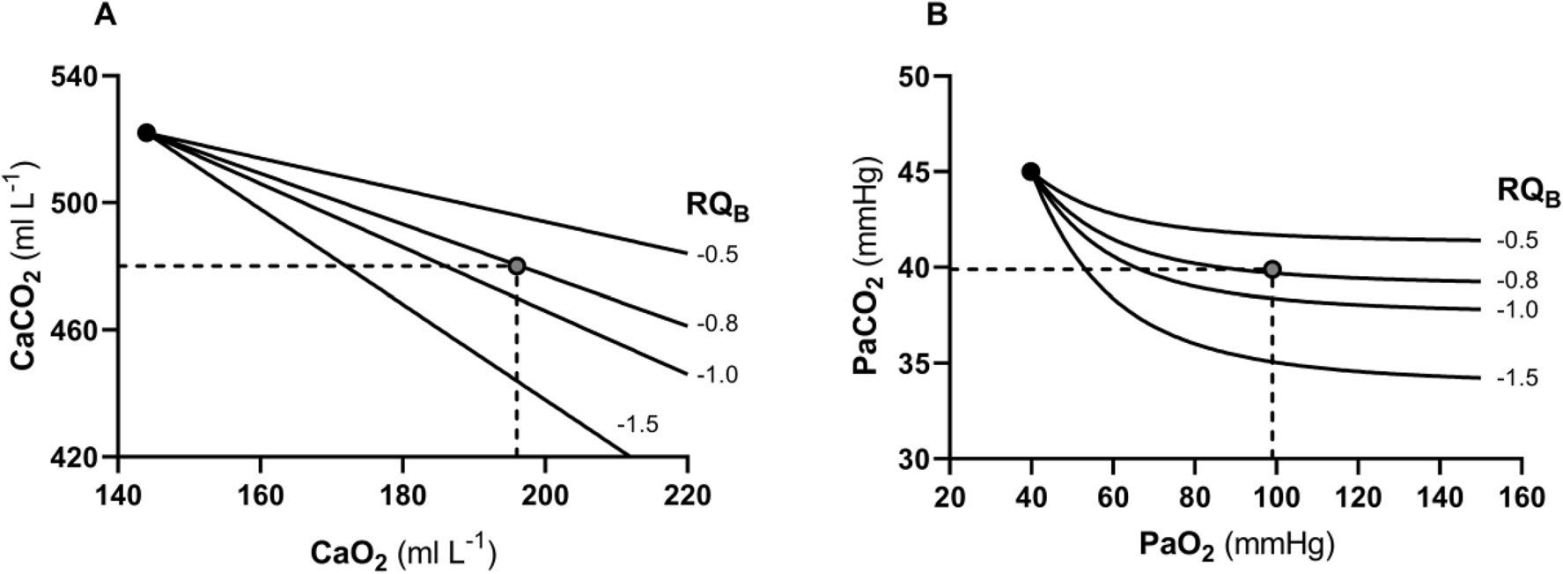
**A** An O_2_–CO_2_ diagram for blood at rest. The black dot represents the mixed venous blood gas composition. Lines representing isopleths of blood respiratory quotient (${RQ}_{B}$) converge on this point. Four of these ${RQ}_{B}$ isopleths are reproduced (black lines). **B** Same as panel A, after transformation of concentrations into partial pressures. The same four isopleths of ${RQ}_{B}$ are reported. The black dot, on which the ${RQ}_{B}$ isopleths converge, represents the mixed venous blood gas composition. The arterial blood gas composition of a resting human is also shown (grey dot). ${C}_{a}{CO}_{2}$ carbon dioxide concentration in arterial blood, ${C}_{a}{O}_{2}$ oxygen concentration in arterial blood, ${P}_{a}{CO}_{2}$ carbon dioxide partial pressure in arterial blood, ${P}_{a}{O}_{2}$ oxygen partial pressure in arterial blood [After Ferretti et al. (2017)]

Equation (﻿13), which, in analogy to the alveolar air equation, we may call the arterial blood equation, tells that, if we plot ${C}_{a}{CO}_{2}$ as a function of ${C}_{a}{O}_{2}$, we obtain a family of straight lines, with negative slopes equal to *RQ*_*B*_, converging on a point, the coordinates of which correspond to the gas composition of mixed venous blood (Fig. 5A). This occurs in case of shunt, *id est* when mixed venous blood is not exposed to gas exchange in the alveolar capillaries, so that ${C}_{a}{O}_{2}={C}_{\overline{v}}{O}_{2}$ and ${C}_{a}{CO}_{2}={C}_{\overline{v}}{CO}_{2}$: in this case, arterial and mixed venous bloods have the same respiratory gas composition.

Respiratory gases diffuse across the alveolar–capillary barrier, driven by differences in partial pressure. Therefore, it may be useful to express Eq. (﻿13) in terms of partial pressures instead of concentration (Ferretti et al. 2017)

14 $${{\beta c}\; P}_{a}{CO}_{2}= {\beta c}\; {P}_{\overline{v}}{CO}_{2} \, +{{RQ}_{B}}\; \beta o \left({P}_{a}{O}_{2}-{P}_{\overline{v}}{O} _{2}\right),$$

where constants *βc* and *βo* are the blood transport coefficients of carbon dioxide and oxygen, respectively. On one side, *βc* can be considered, on first approximation, invariant, because most of blood carbon dioxide is dissolved in plasma, either in pure form or as bicarbonate, whereas the carbon dioxide bound to haemoglobin stays on a segment of the carbon dioxide equilibrium curve that is practically linear. On the other side, contrary to *βc*, *βo* cannot be taken as invariant: almost all oxygen in blood, at physiological ${PO}_{2}$ values at sea level, is bound to haemoglobin, and, due to the shape of the oxygen equilibrium curve, *βo* is lower the higher is the ${PO}_{2}$. Thus, an a-priori model of the oxygen equilibrium curve is a prerequisite for an analytical solution of Eq. (﻿14).

Many inductive empirical models, often polynomial, were proposed, providing detailed descriptions of the oxygen equilibrium curve (see e.g. Adair 1925; Kelman 1966; Margaria 1963; Pauling 1935; Severinghaus 1979; Tien and Gabel 1977). None of them have distinct merits over the others (Myers et al. 1990; O’Riordan et al. 1985).

Conversely, Hill (1910) adopted a theoretical approach, whose quantitative outcomes, however, were confuted when Hill’s prediction of a stoichiometric oxygen/haemoglobin ratio of 2.8 was demonstrated not to correspond to the observation that indeed four molecules of oxygen can bind to each molecule of haemoglobin (Perutz 1970). Notwithstanding, although a predicted value was demonstrated to be incorrect (*n* = 2.8 instead of 4), the deep meaning of Hill’s constant was not undermined: *n* > 1 indicates cooperativity; *n* = 1 absence of cooperativity.

That said, Hill’s model, which provides an accurate and precise description of the oxygen equilibrium curve in the saturation range 0.20–0.98 (Severinghaus 1979), is still a fascinating tool for the analysis of the oxygen equilibrium curve, inasmuch as it is simple—two constants suffice to describe the behaviour of the curve: the slope (*n*) and the *x*-axis intercept of Hill’s plot, and because the latter constant, not yet refuted, is the basis for calculating the oxygen partial pressure sustaining a saturation of 0.50 (*P*_50_). Hill’s *P*_50_ has become a universally used parameter for the analysis of the Bohr effect, also by those who reject Hill’s model.

Application of Hill’s model to an analytical solution of Eq. (﻿14) leads to the construction of the curves reported in Fig. 5B (Rahn and Fenn 1955). In this figure, four isopleths of ${RQ}_{B}$ are shown. Their shape is dictated by the analytical solution given to the oxygen equilibrium curve, in particular by the value taken by *βo* at any ${PO}_{2}$. As for Fig. 5A, all these isopleths converge on a gas composition corresponding to that of mixed venous blood.

**Fig. 6:**
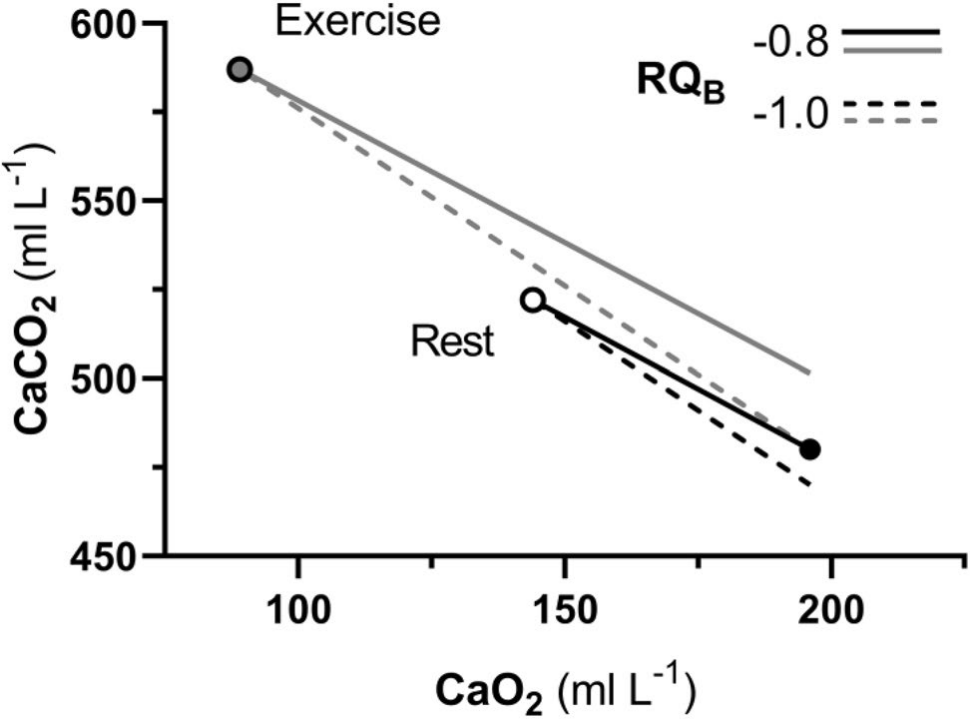
An oxygen–carbon dioxide diagram for blood, on which two steady-state conditions are represented: rest and moderate exercise. The resting values are the same as in Fig. 5A: the arterial blood composition is described by the black dot, the mixed venous blood composition by the white dot. Both dots are on the 0.8 ${RQ}_{B}$ isopleth. The black dot represents the mixed venous blood gas composition. At rest (oxygen consumption, $\dot{V}{O}_{2}$ = 0.3 L min^−1^; blood respiratory quotient, ${RQ}_{B}=$ − 0.8; and thus carbon dioxide production ($\dot{V}{CO}_{2}$) is equal to − 0.24 L min^−1^), for arterial blood, we assumed: a blood haemoglobin concentration of 150 g L^−1^; an arterial oxygen saturation (${S}_{a}{O}_{2}$) of 0.97; pH = 7.4; a bicarbonate concentration of 25 mmol L^−1^ (standard steady-state acid base condition, see above). The isopleth for ${RQ}_{B}=$ 1.0 is also reported. At moderate exercise, we assumed that no lactate accumulation takes place, so that arterial blood bicarbonate concentration and pH remain the same as at rest. We also assumed $\dot{V}{O}_{2}$ = 1.0 L min^−1^, ${RQ}_{B}=$ 1.0 and, being at steady state, equal to the metabolic respiratory quotient (glucose only energy source), and cardiac output as from Cerretelli and di Prampero (1987): 9 L min^−1^. The mixed venous oxygen concentration was then calculated using the Fick equation (Eq. ﻿5b); the mixed venous carbon dioxide concentration was finally obtained by means of Eq. (﻿13) (of course, since ${RQ}_{B}=$ − 1.0, $\dot{V}{CO}_{2}$ = − $\dot{V}{O}_{2}=$ − 1.0 L min^−1^)

At exercise, the ${RQ}_{M}$ increases as a function of the applied power, because the fraction of energy derived from glucose oxidation through the glycolytic pathway becomes progressively larger. At about 60% of the maximal aerobic power, corresponding approximately to the so-called lactate threshold, at steady state, almost all energy is derived from carbohydrates, so that ${RQ}_{M}$ = − 1.0, and so is also ${RQ}_{B}$. In this condition (upper limit of the moderate exercise domain), we can assume that no net lactate accumulation occurs, so that arterial blood pH and ${P}_{a}{CO}_{2}$ remain the same as at rest. If this is so, also ${C}_{a}{CO}_{2}$ would remain unchanged, and thus the arterial blood gas composition. This allows the construction of Fig. 6, which evidences, on the O_2_–CO_2_ diagram for blood, how the blood gas composition is modified during exercise. The most striking feature in Fig. 6 is the remarkable displacement of the mixed venous gas point, on which all the isopleths for ${RQ}_{B}$ converge. This is a direct consequence of the $\dot{V}{O}_{2}$ and $\dot{V}{CO}_{2}$ changes at exercise, which, since the arterial blood gas composition in the analysed condition is invariant, carry along a fall of ${C}_{\overline{v}}{O}_{2}$ and an increase of ${C}_{\overline{v}}{CO}_{2}$. This is the most notable difference between the O_2_–CO_2_ diagrams for blood and for alveolar air: in the latter case, the point on which an ${{RQ}}_{L}$ isopleth crosses the *x*-axis, corresponds to the inspired air composition, and this, at variance with the mixed venous gas composition, is independent of metabolism and of ventilation, being displaced only when we breathe a different gas mixture from air at sea level.

During steady-state rest and moderate exercise, an equilibrium is attained at the venous end of the pulmonary capillaries between end-capillary and alveolar partial pressures of gases. Therefore, if no other factors interfere with this process, arterial blood and alveolar air should have the same partial pressures of oxygen and carbon dioxide, those compatible with the same respiratory quotient in alveolar air as in blood (concepts of ideal air and ideal blood, as formulated by Rahn and Fenn 1955). At rest, when *RQ*_*B*_ = *RQ*_*L*_ is generally approximated to − 0.8, these conditions are met when ${P}_{a}{O}_{2}={P}_{A}{O}_{2}=$ 105 mmHg and at ${P}_{a}{\mathrm{CO}}_{2}={P}_{A}{\mathrm{CO}}_{2}=$ 40 mmHg. A century of blood gas and alveolar gas measurements shows that the latter is the case, the former is not: despite ${P}_{A}{O}_{2}=$ 105 mmHg indeed, ${P}_{a}{O}_{2} =$100 mmHg, so that a positive alveolar–arterial oxygen gradient appears.

**Fig. 7:**
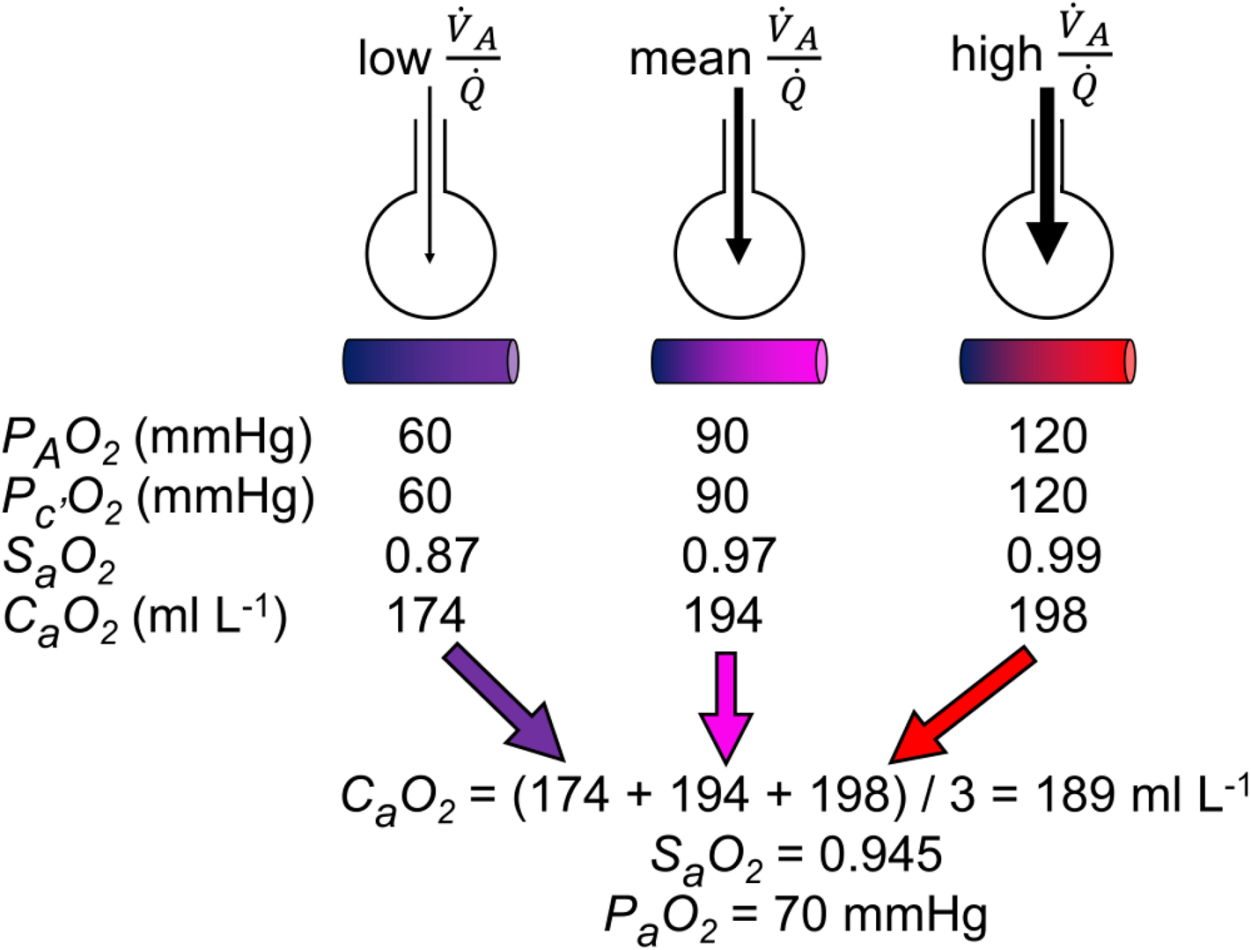
Imagine a hypothetical lung with three huge lung units, each receiving the same blood flow (say 1 L min^−1^ for simplicity), but differently ventilated, such that, from left to right, the first is hypoventilated, the second is normoventilated, and the third is hyperventilated, thus compensating for the first. For each lung unit, the resulting alveolar (${P}_{A}{O}_{2}$) and end-capillary (${P}_{c^{\prime}}{O}_{2}$) oxygen partial pressures are reported, together with the corresponding arterial oxygen saturation (${S}_{a}{O}_{2}$) and arterial oxygen concentration (${C}_{a}{O}_{2}$). When the three units converge to form arterial blood, each contributes 1 L of blood per minute, containing, respectively, 174, 194, and 198 mL of oxygen. Therefore, in 1 min, arterial blood receives 3 L of blood containing a total of 567 mL of oxygen, yielding a ${C}_{a}{O}_{2}$ of 189 mL L^−1^. Despite a mean ${P}_{A}{O}_{2}$ of 90 mmHg, the resulting ${P}_{a}{O}_{2}$ is 70 mmHg only. The hyperventilated lung unit cannot compensate for the hypoventilated lung unit, and an alveolar–arterial oxygen gradient of 20 mmHg is generated. ${\dot{V}}_{A}$ alveolar ventilation, $\dot{Q}$ lung capillary blood flow

Notwithstanding a small contribution from the addition of deoxygenated blood into the systemic arterial circulation from bronchial and Thebesian veins, the occurrence of a positive alveolar–arterial oxygen gradient is essentially the result of the interaction of two phenomena. The first is the heterogeneous distribution of the ventilation/perfusion ratio (${\dot{V}}_{A}/\dot{Q}$), which is not simply due to a gravitational effect (Prisk et al. 1995). ${\dot{V}}_{A}/\dot{Q}$ can vary between two extremes: the lowermost extreme is represented by ${\dot{V}}_{A}/\dot{Q} =$ 0 (perfused, but non ventilated lung units), in which case ${P}_{A}{O}_{2}=$ ${P}_{\overline{v}}{O}_{2}$; the uppermost extreme is represented by ${\dot{V}}_{A}/\dot{Q} =$ ∞ (ventilated, but non perfused lung units), in which case ${P}_{A}{O}_{2}={P}_{I}{O}_{2}$ (Farhi and Rahn 1955). The second phenomenon is related to the characteristics of the oxygen equilibrium curve. Lung units with low ${\dot{V}}_{A}/\dot{Q}$ are characterised by lower ${P}_{A}{O}_{2}$ and higher ${P}_{A}{CO}_{2}$ than lung units with high ${\dot{V}}_{A}/\dot{Q}$. The respiratory gas composition of arterial blood is a perfusion-weighted^5^ average of all gas compositions provided by all open lung capillaries, exposed to different values of ${\dot{V}}_{A}/\dot{Q}$, and all in equilibrium with the corresponding alveoli, at least at rest and moderate exercise. Since the relationship between ${C}_{a}{CO}_{2}$ and ${P}_{a}{CO}_{2}$ is linear in the physiological range, alveoli with high ${\dot{V}}_{A}/\dot{Q}$ can compensate for alveoli with low ${\dot{V}}_{A}/\dot{Q}$, thus ensuring ${P}_{a}{CO}_{2}={P}_{A}{CO}_{2}$. This compensation cannot occur for oxygen, as long as alveoli with low ${\dot{V}}_{A}/\dot{Q}$ attain an equilibrium with the corresponding capillaries on or close to the steep part of the oxygen equilibrium curve, so that arterial oxygen saturation (${S}_{a}{O}_{2}$) and ${C}_{a}{O}_{2}$ are lowered; in contrast, alveoli with elevated ${\dot{V}}_{A}/\dot{Q}$ attain an equilibrium with the corresponding capillaries on the flat part of the oxygen equilibrium curve, in a part on which any increase in ${P}_{A}{O}_{2}$ cannot provide an increase in ${S}_{a}{O}_{2}$, and thus in ${C}_{a}{O}_{2}$. These capillaries cannot increase their ${C}_{a}{O}_{2}$, and thus cannot compensate for the effect of alveoli with low ${\dot{V}}_{A}/\dot{Q}$: therefore, the resulting ${P}_{a}{O}_{2}$ turns out lower than the mean ${P}_{A}{O}_{2}$. This state of things is described with a theoretical simplified lung model in Fig. 7.

In real lungs, topographic heterogeneity of ${\dot{V}}_{A}/\dot{Q}$ distribution is more important when lung blood flow is low. This implies that during moderate exercise, when lung blood flow is increased, ${\dot{V}}_{A}/\dot{Q}$ is less heterogeneously distributed than at rest, as demonstrated using radioactive tracers (Bake et al. 1968; Bryan et al. 1964; Harf et al. 1978) or inert gases’ manipulation techniques (Beck et al. 2012). Recruitment of capillaries, which are closed at rest, to sustain the increase in blood flow, may contribute significantly to this phenomenon by reducing diffusion distances between alveoli and capillaries. However, when the multiple inert gas elimination technique is used, the opposite is observed (Domino et al. 1991, 1993; Gale et al. 1985; Gledhill et al. 1978; Hammond et al. 1986). The reasons of this discrepancy during moderate exercise are yet to be understood (Wagner 1992, 2015). At variance, during sustained heavy exercise, evidence suggests that pulmonary perfusion heterogeneity is increased in humans (Burnham et al. 2009), suggesting the possibility that interstitial pulmonary edema may develop in this condition, thus explaining both spatial perfusion heterogeneity and ${\dot{V}}_{A}/\dot{Q}$ heterogeneity.

## The ventilation–perfusion equation

Let us now return to Eq. (﻿8) and solve it for ${\dot{V}}_{A}$

15 $${\dot{V}}_{A}=-\frac{{\dot{V}}_{R}{CO}_{2}}{{F}_{A}{CO}_{2}}.$$

If we introduce a correction factor accounting for the fact that ${\dot{V}}_{A}$ is expressed in BTPS and $\dot{V}{CO}_{2}$ in STPD, and we transform ${F}_{A}{CO}_{2}$ in ${P}_{A}{CO}_{2}$ by means of Dalton’s law, we obtain

16 $${\dot{V}}_{A}=-\frac{{Cg }\; {\dot{V}}_{R}{CO}_{2}}{{P}_{A}{CO}_{2}},$$

where *Cg* is the ratio of the STPD to BTPS and the ${P}_{A}{CO}_{2}$ to ${F}_{A}{CO}_{2}$ conversion factors (Rahn and Fenn 1955; Otis 1964).^6^ Since

17 $${\dot{V}}_{R}{CO}_{2}={RQ}_{L}\; {\dot{V}}_{R}{O}_{2},$$

we can also write

18 $${\dot{V}}_{A}=-\frac{{Cg}\;{RQ}_{L}\; {\dot{V}}_{R}{O}_{2}}{ {P}_{A}{CO}_{2}},$$

whence

19 $${\dot{V}}_{R}{O}_{2}=-\frac{{\dot{V}}_{A}\; {P}_{A}{CO}_{2}}{{Cg}\;{RQ}_{L}}.$$

Since we are at steady state, we can also write

20 $$-\frac{{\dot{V}}_{A}\; {P}_{A}{CO}_{2}}{{Cg}\; {RQ}_{L}}=\dot{Q} \left({C}_{a}{O}_{2}-{C}_{\overline{v}}{O}_{2}\right),$$

whence

21 $$-\frac{{\dot{V}}_{A}\;{P}_{A}{CO}_{2}}{{Cg}\; {RQ}_{L} \; \dot{Q} \left({C}_{a}{O}_{2}-{C}_{\overline{v}}{O}_{2}\right)}=1.$$

Rearranging, we obtain

22 $$\frac{{\dot{V}}_{A} }{\dot{Q}}=-\frac{{Cg}\; {RQ}_{L} \left({C}_{a}{O}_{2}-{C}_{\overline{v}}{O}_{2}\right) }{{P}_{A}{CO}_{2}}.$$

Equation (22) is a formulation of the ventilation–perfusion equation (Rahn and Fenn 1955; Otis 1964), and sets the homeostatic equilibrium between alveolar air and blood. It states that, *ceteris paribus*, ${\dot{V}}_{A}/\dot{Q}$, which is heterogeneously distributed throughout the lungs, is directly proportional to − ${RQ}_{L}$ and to $({C}_{a}{O}_{2}-{C}_{\overline{v}}{O}_{2})$ and inversely proportional to ${P}_{A}{CO}_{2}$. Therefore, each ${P}_{A}{O}_{2}$ value in Fig. 3 corresponds not only to one ${RQ}_{L}$ value, but also to a unique value of ${\dot{V}}_{A}/\dot{Q}$. This value is lower the closer ${RQ}_{L}$ is to zero.

Equation (﻿22) applies to specific values of ${\dot{V}}_{A}$ and $\dot{Q}$, and thus to given metabolic levels. Since, within the aerobic exercise domain at sea level, as long as ${P}_{A}{CO}_{2}$ stays invariant and equal to 40 mmHg, also ${\dot{V}}_{A} /{\dot{V}}_{R}{CO}_{2}$ stays invariant and equal to − 21.6, we can derive a more general equation by combining Eqs.  (﻿5b) and (﻿22) and rearranging, as follows:

23 $$\frac{{\dot{V}}_{A}}{{\dot{V}}_{R}{CO}_{2}}=\frac{{\dot{V}}_{A}}{\dot{Q} ({C}_{a}{CO}_{2}-{C}_{\overline{v}}{CO}_{2}) }=\frac{{Cg}\; {RQ}_{L}\, \left({C}_{a}{O}_{2}-{C}_{\overline{v}}{O}_{2}\right)}{ {P}_{A}{CO}_{2} \, \left({C}_{\overline{v}}{CO}_{2}{-C}_{a}{CO}_{2}\right)}=-21.6.$$

During exercise, as compared to rest, ${\dot{V}}_{A}$ increases in direct proportion to $-\dot{V}{CO}_{2}$, whereas the increase of $\dot{Q}$ is less. Therefore, if ${\dot{V}}_{A} /{\dot{V}}_{R}{CO}_{2}$ stays invariant, also ${C}_{\overline{v}}{CO}_{2}{-C}_{a}{CO}_{2}$ must increase, because of higher ${C}_{\overline{v}}{CO}_{2}$. Moreover, the $\dot{V}_{A}/{\dot{Q}}$ grows, being higher the higher is the metabolic rate, inasmuch as, not only ${C}_{\overline{v}}{CO}_{2}-{C}_{a}{CO}_{2}$ goes up, but ${RQ}_{L}$ = ${RQ}_{M}$ tends to approach − 1, due to a progressive shift toward carbohydrate oxidation. It is noteworthy, however, that, as long as we stay at sea level, and thus, we operate on the flat part of the oxygen equilibrium curve, ${C}_{a}{O}_{2}$ does not vary, at least at rest and during moderate exercise, so that an increase of $({C}_{a}{O}_{2}-{C}_{\overline{v}}{O}_{2})$ can be sustained solely by a fall of ${C}_{\overline{v}}{O}_{2}$. By analogy, as long as ${P}_{a}{CO}_{2}$ stays invariant, and so does ${C}_{a}{CO}_{2}$, a decrease of $({C}_{a}{CO}_{2}-{C}_{\overline{v}}{CO}_{2}$) can be sustained solely by an increase in ${C}_{\overline{v}}{CO}_{2}$.

The tight matching between ${\dot{V}}_{A}$ and $-\dot{V}{CO}_{2}$ reflects a fine regulation, mostly centred on the modulation of ventilation by the activity of central chemoreceptors. This equilibrium is broken in case of hyperventilation: the ${\dot{V}}_{A} /{\dot{V}}_{R}{CO}_{2}$ goes down (becomes more negative), a new steady state is attained at a lower ${P}_{a}{CO}_{2}$, and at a higher ${P}_{a}{O}_{2}$, than those incurring during normoventilation (${P}_{a}{CO}_{2}=$ 40 mmHg) at any given ${P}_{I}{O}_{2}$. This occurs, e.g., at high altitude, because of hypoxic stimulation of peripheral chemoreceptors, or in case of a larger ${\dot{V}}_{A}/\dot{Q}$ heterogeneity in the lungs, due to the presence of either non ventilated lung regions or increased physiological dead space (unperfused lung regions). The equilibrium is broken in the opposite direction in case of hypoventilation, as in respiratory failure or paralysis of the respiratory centres.

## Diffusion–perfusion interaction in alveolar–capillary gas transfer

The homeostatic equilibria discussed so far, leading to a tight coupling of respiration to metabolism, rely on the implicit assumption of complete gas equilibration between alveolar air and capillary blood. In normoxic humans at rest and at steady-state exercise up to the critical power, provided the alveolar–capillary barrier be intact, such equilibration may occur indeed in each open pulmonary capillary in contact with an alveolus, independent of its ${\dot{V}}_{A}/\dot{Q}$, inasmuch as the contact time between alveolar air and capillary blood is long enough. According to Wagner and West (1972), at rest, full equilibration between the two sides of the alveolar–capillary barrier is attained when the blood has completed about one-third of the capillary length, a distance that Heller and Schuster (2007) have reduced to one-seventh. This provides a remarkable reserve, to be exploited during exercise, for alveolar–capillary gas equilibration. The same authors have suggested, based on the data of Borland et al. (2001), that this is the case also in the heavy exercise domain, thanks to this reserve. The report by Hakim et al. (1994) that a four-time increase in pulmonary blood flow is accompanied by a reduction by one half of the capillary transit time suggests alveolar–capillary equilibration over a large spectrum of exercise intensities. In non-athletic subjects, the extreme limits of this functional equilibrium may be attained at maximal exercise (Heller and Schuster 2007).

In athletic subjects, with elevated maximal $\dot{V}{O}_{2}$ and maximal $\dot{Q}$, no alveolar–capillary gas equilibration is attained at maximal exercise, incidentally an unsteady-state condition. Thus, the phenomenon of exercise-induced arterial hypoxaemia, which was reported for the first time by Harrop (1919), appears around maximal exercise (Dempsey et al. 1984; Dempsey and Wagner 1999), and eventually even in submaximal exercise, especially in women (Dominelli et al. 2013; Harms et al. 1998). Besides diffusion limitation in alveolar–capillary oxygen transfer, which we discuss here below, insufficient hyperventilation due to expiratory flow limitation, and increased ${\dot{V}}_{A}/\dot{Q}$ heterogeneity at maximal exercise have also been called upon as possible mechanisms behind exercise-induced arterial hypoxaemia (Dempsey and Wagner 1999; Nielsen 2003; Prefaut et al. 2000). Some functional consequences of exercise-induced arterial hypoxaemia on oxygen flow during maximal exercise have been discussed elsewhere (Ferretti 2014). Incidentally, it is noteworthy that full equilibration in alveolar–capillary gas exchange is still compatible with the occurrence of a positive alveolar–arterial oxygen gradient, the nature of which, as discussed above, is of different origin.

**Fig. 8:**
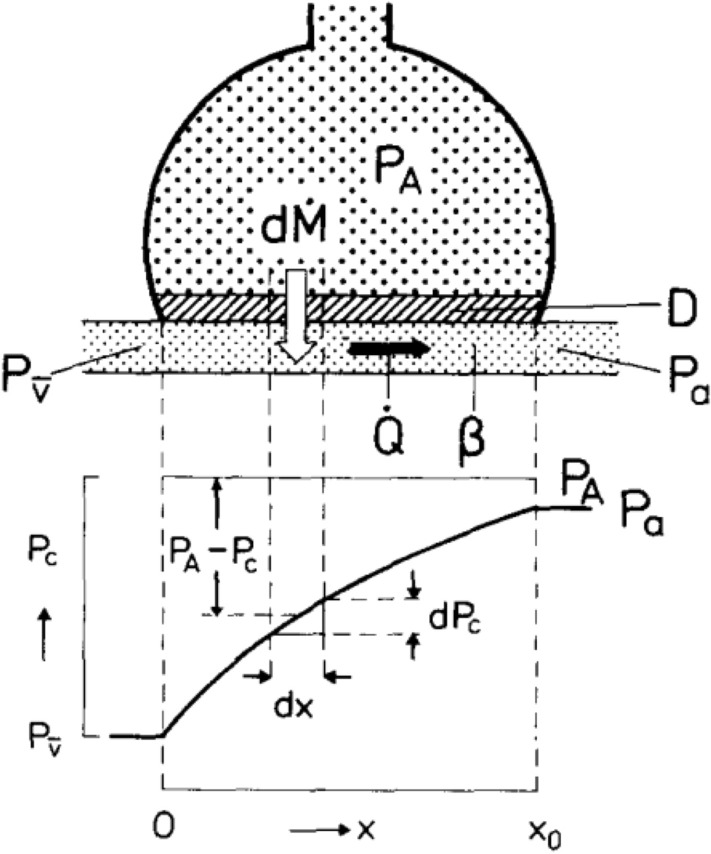
A scheme of the simplified model of the lung. The model implies numerous simplifying assumptions: steady state, diffusion occurring across a flat homogeneous alveolar–capillary barrier, and along a direction *D* that is perpendicular to the barrier; linear blood dissociation curve (constant gas transport coefficient for blood, *β*); homogeneous membrane and blood flow ($\dot{Q}$); absence of shunt. The graph describes the exponential increase of gas partial pressure inside the capillary, as blood proceeds along it. The flow across the barrier follows the pressure gradient. $d\dot{M}$ infinitesimal molar flow of the gas, *P*_*A*_ alveolar gas pressure, $P\overline{v }$ mixed venous gas pressure, *P*_*a*_ arterial gas pressure, *P*_*c*_ capillary gas pressure, *x* distance along the capillary, *0* beginning of pulmonary capillary, *X*_*0*_ end of capillary, where ${P}_{c^{\prime}}{O}_{2}$ is attained [From Piiper and Scheid (1981)]

The hypothesis of diffusion limitation to explain exercise-induced arterial hypoxaemia stems from Piiper’s model of alveolar gas exchange (Piiper and Scheid 1981), characterised by the diffusion–perfusion interaction equations. The model’s axiom is that alveolar–capillary gas transfer results from two interacting mechanisms: diffusion across the alveolar–capillary barrier, and lung capillary perfusion. At steady state, the flow of any gas removed from or added to the lungs through the airways is equal to the flow of gas crossing the alveolar–capillary barrier. A simple model of alveolar–capillary gas exchange can be constructed by imagining the mean pulmonary blood flow in contact with the mean alveolar air, with oxygen diffusing across a thin membrane separating capillary blood from alveolar air (see Fig. 8). In such a system, the gas flow $\dot{V}$ across the membrane of given surface area *A* and thickness *L* is directly proportional to the pressure gradient across that membrane

24 $$\dot{V}= \Delta P \left(\frac{{d}\;{s}\;{A}}{L}\right)=\Delta P \;{D}_{L},$$

where *d* and *s* are the diffusion and solubility constants in the barrier of the gas at stake, and ${D}_{L}$ is a lumped constant, which we call lung diffusing capacity (Bates et al. 1955; Wagner 1977). ∆*P* is the effective pressure gradient, id est the difference between ${P}_{A}{O}_{2}$ and the pressure existing in the capillary at a given distance *δ* from the venous entrance [${P}_{c}$(*δ*)].

Restricting the analysis to oxygen, the diffusive oxygen flow (${\dot{V}}_{L}{O}_{2})$ across the barrier at any distance *δ* turns out equal to

25 $$d{\dot{V}}_{L}{O}_{2}=\left[{P}_{A}{O}_{2}-{P}_{c}{O}_{2}\left(\delta \right)\right]{D}_{L},$$

where ${P}_{c}{O}_{2}$ (*δ*) is the oxygen partial pressure in a lung capillary at distance *δ* from the arterial pulmonary entrance (venous blood). The infinitesimal $d\dot{V}{O}_{2}$ across the barrier raises ${P}_{c}{O}_{2}$(*δ*) as we proceed along the capillary from the venous to the arterial side.

The rate of increase of ${P}_{c}{O}_{2}$(*δ*) is inversely proportional to the lung capillary blood flow (set equal to $\dot{Q}$ in absence of shunt) and to the blood transport coefficient *β* of the gas at stake (for oxygen, *βo*)

26 $$d{\dot{V}}_{L}{O}_{2}=\dot{Q}\;\beta o\; d{P}_{c}{O}_{2}.$$

For inert gases, *β* is a constant that is independent of the gas pressure. For oxygen, *βo* depends on ${P}_{c}{O}_{2}$ because of the characteristics of the oxygen equilibrium curve. If for simplification, we assume *βo* invariant (this assumption is discussed below), the ${P}_{c}{O}_{2}$ along the capillary tends to an asymptote corresponding to ${P}_{A}{O}_{2}$ (Krogh 1922). On this basis, at steady state, combination of Eqs. (﻿25) and (﻿26), which provide equal values of $d{\dot{V}}_{L}{O}_{2}$, and subsequent integration along the capillary length, yields (Piiper and Scheid 1981)

27 $$\frac{{P}_{A}{O}_{2}-{P}_{c^{\prime}}{O}_{2}}{{P}_{A}{O}_{2}-{P}_{\overline{v}}{O}_{2}}={e}^{-\frac{{D}_{L}}{\dot{Q}\; \beta o}},$$

where ${P}_{c^{\prime}}{O}_{2}$ is end-capillary ${PO}_{2}$. This equation is called the lung diffusion–perfusion interaction equation. Piiper and Scheid (1981) named the left-hand side of Eq. (﻿27) equilibration deficit. Its value depends only on the equilibration coefficient (${K}_{e}$), defined as the module of the exponent of the right-hand side of Eq. (27). In fact, ${K}_{e}$ is the ratio of two conductances in series, namely the diffusive conductance across the barrier (${D}_{L}$) and the perfusive conductance ($\dot{Q}\; \beta o$) (Piiper and Scheid 1981), and is therefore dimensionless.

The larger is ${K}_{e}$, the closer to zero is the equilibration deficit. Therefore, when ${K}_{e}$ is large (> 3), the equilibration deficit approaches 0, so that ${P}_{c^{\prime}}$ is practically equal to ${P}_{A}$, alveolus and capillary have attained equilibrium and the gas flow is limited by perfusion. On the other side, when ${K}_{e}$ is sufficiently small (< 0.1), the equilibration deficit gets close to 1, ${P}_{c^{\prime}}$ approaches ${P}_{\overline{v} }$, and the gas flow is limited by diffusion.

For oxygen, most of which is carried by haemoglobin, diffusion limitation occurs when we operate on the steep part of the oxygen equilibrium curve (high $\beta o$), as in deep hypoxia at altitude or in case of enlarged $\frac{{\dot{V}}_{A}}{\dot{Q}}$ heterogeneity. This also occurs in endurance athletes, who undergo exercise-induced arterial hypoxaemia. On the contrary, perfusion limitation occurs when we operate on the flat part of the oxygen equilibrium curve (low $\beta o$), as in normoxia or in hyperoxia. The flow of carbon monoxide, which has an extremely high affinity for haemoglobin (very high *β*), is limited by diffusion. For all inert gases, which do not bind to haemoglobin, and thus are subject only to the law of Henry, gas flow is limited by perfusion. In this case, ${K}_{e}$ is roughly inversely proportional to the gas molecular weight (Kawashiro et al. 1975). Generally speaking, there is perfusion limitation if *β* is high; there is diffusion limitation when *β* tends to 0. Differences in ${K}_{e}$ among gases depend only on their diffusion and solubility constants (thus on ${D}_{L}$), and on *β* as long as ${K}_{e}$ is proportional to the ratio *d* *s/β*. This general principle is crucial in determining the size of the alveolar–arterial oxygen gradient.

We define contact time (*t*_*c*_) the time taken by blood to proceed along a capillary from one side to the other. It is equal to the ratio of the effective lung capillary blood volume (${q}_{c}$) to $\dot{Q}$. So ${K}_{e}$ can be expressed as follows:

28 $${K}_{e}=\frac{{D}_{L}\; {t}_{c}}{ {q}_{c}\; \beta o}.$$

At rest, ${q}_{c}$ is assumed invariant and independent of $\dot{Q}$. At exercise, the increase of $\dot{Q}$ carries along not only an increase in *q*_*c*_, due to the recruitment of previously closed capillaries, but also a fall of ${t}_{c}$, because the increase in $\dot{Q}$ overrides that in ${q}_{c}$. Therefore, ${K}_{e}$ is lowered at exercise, and the equilibration deficit is higher. This tendency enhances the probability of observing diffusion limitation at exercise with respect to rest. Diffusion limitation is a major determinant of the increase in the alveolar–arterial oxygen difference during heavy exercise in individuals characterised by very high levels of maximal $\dot{V}{O}_{2}$ in normoxia, or in normal people in hypoxia (Wagner 2015).

This model of the alveolar–capillary gas transfer assumes not only steady state, but also constancy of $\beta$ and homogeneity of the alveolar–capillary barrier. The former assumption is ensured for inert gases which do not bind to transport proteins: they are merely dissolved in plasma and the Henry’s law applies to them. It can also be assumed for carbon dioxide, for its dissociation curve can be approximated to a straight line in the physiological partial pressure range. It cannot be assumed for oxygen, due to the allosteric characteristics of the oxygen equilibrium curve. A simplification making this assumption plausible is to take the mean slope of the oxygen equilibrium curve ($\overline{\beta o }$), which is equal to

29 $$\overline{\beta o }=\frac{{C}_{a}{O}_{2}-{C}_{\overline{v}}{O}_{2}}{{P}_{a}{O}_{2}-{P}_{\overline{v}}{O}_{2}}.$$

This implies that $\overline{\beta o }$ is higher the closer the ${P}_{a}{O}_{2}$ is to the steep part of the oxygen equilibrium curve, thus being higher in hypoxia than in normoxia, coherently with the notion, expressed above, that ${K}_{e}$ is low in hypoxia (diffusion limitation) and high in normoxia (perfusion limitation).

The second assumption, that of a homogeneous alveolar–capillary barrier, is false both structurally, as demonstrated by morphometric analysis of the lungs (Constantinopol et al. 1989; Weibel 1972, 1999), and functionally, as long as lung capillary perfusion is heterogeneously distributed both in space and in time (Glenny and Robertson 1990, 2011; Hlastala et al. 1996). The simplified model of the lung used by Piiper and Scheid (1981) circumvents this problem by admitting an average alveolar–capillary barrier. The effects of a heterogeneous distribution of $D_{L}$ through the lungs on the alveolar–capillary gas transfer were analysed by Piiper (1992).

Similar models were used to analyse gas transfer in peripheral systemic capillaries (Piiper et al. 1984; Piiper and Scheid 1999), after accounting for the fact that oxygen is consumed in the cells, which has an impact on intracellular oxygen gradients. These models are variants of the classical Krogh’s cylinder model (Krogh 1919), in which the entire diffusion resistance is confined into the boundary between tissue and blood (Piiper et al. 1984). For a deep and thorough discussion of the subject of peripheral gas exchange, we refer the readers to the beautiful review by David Poole and coworkers in this series (Poole et al. 2022﻿).

## On the cardiovascular responses to exercise

**Fig. 9:**
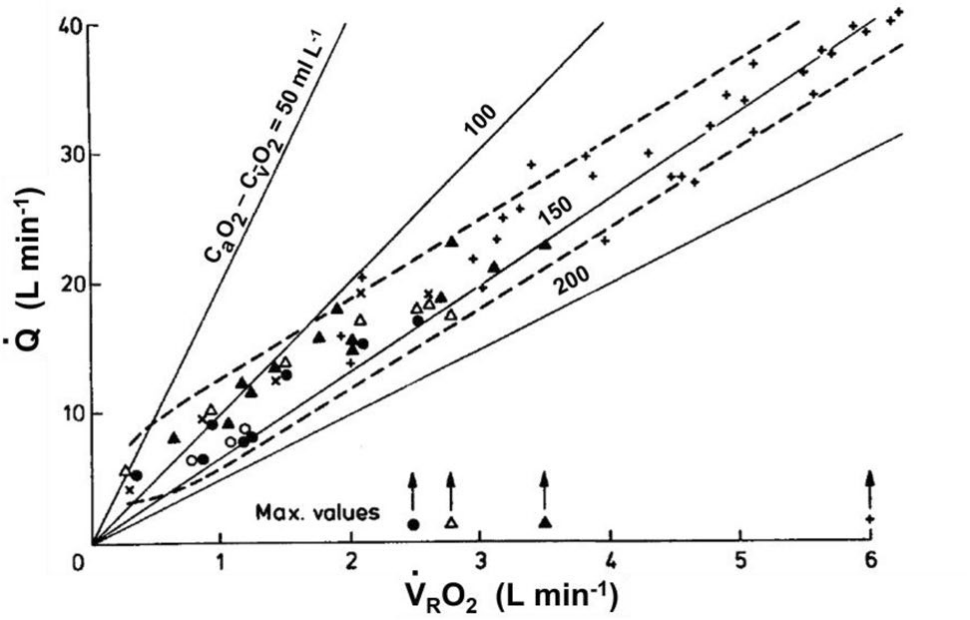
Cardiac output ($\dot{Q}$) as a function of oxygen flow ($\dot{V}{O}_{2}$ in the figure, ${\dot{V}}_{R}{O}_{2}$ in the text) for various groups of subjects of different maximal oxygen consumption. Each symbol designates a group of subjects. Isopleths showing arterial-mixed-venous oxygen difference in ml of oxygen per L of blood (${C}_{a}{O}_{2}-{C}_{\overline{v}}{O}_{2}$ in the text) are also drawn [Modified after Cerretelli and di Prampero (1987)]

Coupling of respiration to metabolism in blood during exercise is largely ensured by the increase in $\dot{Q}$ (Asmussen and Nielsen 1952; Åstrand et al. 1964; Hermansen et al. 1970; Stenberg et al. 1967). At steady state, $\dot{Q}$ has been treated as a linear function of ${\dot{V}}_{R}{O}_{2}$^7^ (Åstrand et al. 1964; Cerretelli and di Prampero 1987), but it is not directly proportional to ${\dot{V}}_{R}{O}_{2}$. The relation has a positive *y*-intercept, implying that $\dot{Q}$ at exercise is not proportional to ${\dot{V}}_{R}{O}_{2}$: in fact at maximal exercise, in young non-athletic subjects, $\dot{Q}$ is only four times higher than at rest, whereas ${\dot{V}}_{R}{O}_{2}$ is about ten times higher than at rest. Therefore, according to Eq. (﻿5a), the increase of ${\dot{V}}_{R}{O}_{2}$ at exercise and its equality with $\dot{V}{O}_{2}$ is made possible only by the simultaneous increase of ${C}_{a}{O}_{2}-{C}_{\overline{v}}{O}_{2}$. This is clearly shown in Fig. 9. Therefore, two variables determine the tight matching of ${\dot{V}}_{R}{O}_{2}$ with $\dot{V}{O}_{2}$ at exercise: $\dot{Q}$ and ${C}_{a}{O}_{2}-{C}_{\overline{v}}{O}_{2}$.

The increase in $\dot{Q}$ results from the simultaneous increase of its two determinants, *f*_*H*_ and the stroke volume (${Q}_{s}$). This increase is classically attributed to a re-modulation of autonomic control of heart activity at exercise (Fagraeus and Linnarsson 1976; Robinson et al. 1966). The increase of *Q*_*s*_ is also attributed to the effect of the Frank–Starling mechanism (Rowell et al. 1996). During steady-state dynamic exercise, *f*_*H*_ increases linearly with ${\dot{V}}_{R}{O}_{2}$. The increase of *Q*_*s*_ as a function of ${\dot{V}}_{R}{O}_{2}$ was not treated mathematically, but Åstrand et al. (1964) considered it as non-linear. If the latter were the case, it would not be compatible with a linear $\dot{Q}$ versus ${\dot{V}}_{R}{O}_{2}$ relationship.

At rest, the largest fraction of $\dot{Q}$ goes to the kidneys. At exercise, most of the increase in $\dot{Q}$ goes into the active muscle mass. Muscle blood flow increases dramatically, due to the stimulation of *β*_2_-adrenergic receptors, which are present in muscle arterioles instead of α_1_-adrenergic receptors, and to the action of local vasodilating mediators that are liberated as muscles contract (Casey and Joyner 2011; Delp and Laughlin 1998; Delp and O’Leary 2004; Hearon and Dinenno 2016; Mortensen and Saltin 2014; Saltin et al. 1998; Seals and Victor 1991). The activation of ATP-sensitive potassium channels by nitric monoxide plays also an important role in muscle vasodilation (Dora 2016; Schrage et al. 2006). Muscle vasodilation is almost immediate at the beginning of dynamic exercise and leads to a sudden increase in muscle blood flow (Chin et al. 2010; Clifford 2007; DeLorey et al. 2003; Ferretti et al. 1995) and to a dramatic fall of peripheral resistance (*R*_*p*_) (Elstad et al. 2009; Faisal et al. 2010; Lador et al. 2006, 2008; Wieling et al. 1996). After a transient initial decrease, systolic arterial pressure increases, whereas diastolic blood pressure, if anything, tends to go down (Rowell et al. 1968).

The reported linear $\dot{Q}$ versus ${\dot{V}}_{R}{O}_{2}$ relationship is remarkably stable in a variety of conditions, including aerobic exercise training, following which a decrease of ${f}_{H}$ compensates for an increase of ${Q}_{s}$, so that at any given ${\dot{V}}_{R}{O}_{2}$, $\dot{Q}$ remains unchanged (Ekblom et al. 1968; Saltin et al. 1968). The same is the case for athletes, who have the same $\dot{Q}$ versus ${\dot{V}}_{R}{O}_{2}$ relationship as non-athletes, with lower ${f}_{H}$ and higher ${Q}_{s}$ (Ekblom and Hermansen 1968). The superposition of arm exercise to leg exercise (Bevegård et al. 1966; Secher et al. 1977) and the exercise mode (Hermansen et al. 1970) do not alter the $\dot{Q}$ versus ${\dot{V}}_{R}{O}_{2}$ relationship. Water immersion and supine posture, despite their acute effects on central blood volume with consequent decrease in ${f}_{H}$ and increase in ${Q}_{s}$, leave the $\dot{Q}$ versus ${\dot{V}}_{R}{O}_{2}$ relationship unchanged (Bevegård et al. 1966; Leyk et al. 1994; Rennie et al. 1971; Sheldahl et al. 1987). Analogous effects are obtained with exposure to lower body negative pressure, although in some cases, heart rate compensation was incomplete (Chang et al. 1994; Eiken and Bjurstedt 1985; Fagoni et al. 2020). Exercise in the heat, whose effects on ${f}_{H}$ and ${Q}_{s}$ are opposite to those of water immersion, does not affect the $\dot{Q}$ versus ${\dot{V}}_{R}{O}_{2}$ relationship, as well. Only in extreme heat $\dot{Q}$ becomes lower at any given ${\dot{V}}_{R}{O}_{2}$, due to the extreme fall of *R*_*p*_ occurring when simultaneous strong vasodilation of both the muscular and cutaneous districts intervenes in association with the reduction of plasma volume due to massive sweating (Nadel et al. 1979; Rowell 1974; Périard et al. 2021). Splanchnic and renal vasoconstriction tend to mitigate these effects (Rowell 1974). The classical view was that $\dot{Q}$ is maintained by opposite compensatory changes on ${f}_{H}$ and ${Q}_{s}$, thanks to a short-term modulation by the autonomic nervous system (Cerretelli and di Prampero 1987).

Notwithstanding, there are two conditions in which the $\dot{Q}$ versus ${\dot{V}}_{R}{O}_{2}$ relationship appears to be shifted upwards with respect to that observed at sea level: acute hypoxia (Hartley et al. 1973; Hughes et al. 1968; Roca et al. 1989; Stenberg et al. 1966) and moderate carbon monoxide poisoning (Ekblom et al. 1975; Vogel and Gleser 1972). Both these conditions lead to a reduction of ${C}_{a}{O}_{2}$. Conversely, when ${C}_{a}{O}_{2}$ is increased, such as upon return to sea level after altitude acclimatization, the $\dot{Q}$ versus ${\dot{V}}_{R}{O}_{2}$ relationship is shifted downward (Ferretti et al. 1990; Steinacker et al. 1996). Ferretti et al. (1990) hypothesized that $\dot{Q}$ is inversely proportional to ${C}_{a}{O}_{2}$, such that their product, equal to the systemic oxygen delivery (${\dot{Q}}_{a}{O}_{2}$), is kept constant at any given ${\dot{V}}_{R}{O}_{2}$. This suggests that ${\dot{Q}}_{a}{O}_{2}$ rather than $\dot{Q}$ may be the regulated variable at exercise steady state, varying at exercise as a function of ${\dot{V}}_{R}{O}_{2}$ in such a way as to ensure tight coupling between $\dot{V}{O}_{2}$ and ${\dot{V}}_{R}{O}_{2}$. Ferretti et al. (1992) showed that this was the case indeed, demonstrating the postulated relationship of inverse proportionality between $\dot{Q}$ and ${C}_{a}{O}_{2}$; this finding was later confirmed by Calbet et al. (2006) on a wider basis.

Ferretti et al. (1992) investigated also the relationship between ${\dot{Q}}_{a}{O}_{2}$ and power in the moderate exercise power range. They found it to be linear and parallel to that between $\dot{V}{O}_{2}$ and power. Therefore, the vertical difference between the two lines, corresponding to the oxygen flow in mixed venous blood (${\dot{Q}}_{\overline{v}}{O}_{2}= \dot{Q}\; {C}_{\overline{v}}{O}_{2}$), is a constant, independent of the exercise intensity. The constant ${\dot{Q}}_{\overline{v}}{O}_{2}$ depends on ${S}_{a}{O}_{2}$ (Anchisi et al. 2001) and thus on ${C}_{a}{O}_{2}$, being lower in hypoxia than in acute normoxia.

## The $\dot{{{Q}}} - {\dot{{{V}}}}_{{{R}}}{{O}}_{2}$ diagram

Assuming constant ${\dot{Q}}_{\overline{v}}{O}_{2}$, Adami et al. (2014) analysed the $\dot{Q}$ versus ${\dot{V}}_{R}{O}_{2}$ relationship from a different perspective. In fact, ${\dot{Q}}_{\overline{v}}{O}_{2}$ is equal to

30 $${\dot{Q}}_{\overline{v}}{O}_{2}=K= {\dot{Q}}_{a}{O}_{2}-{\dot{V}}_{R}{O}_{2}=\dot{Q}\;{C}_{a}{O}_{2}-{\dot{V}}_{R}{O}_{2},$$

whose solution for $\dot{Q}$ is

31 $$\dot{Q}= \frac{{\dot{V}}_{R}{O}_{2}+ {\dot{Q}}_{\overline{v}}{O}_{2}}{{C}_{a}{O}_{2}}.$$

**Fig. 10:**
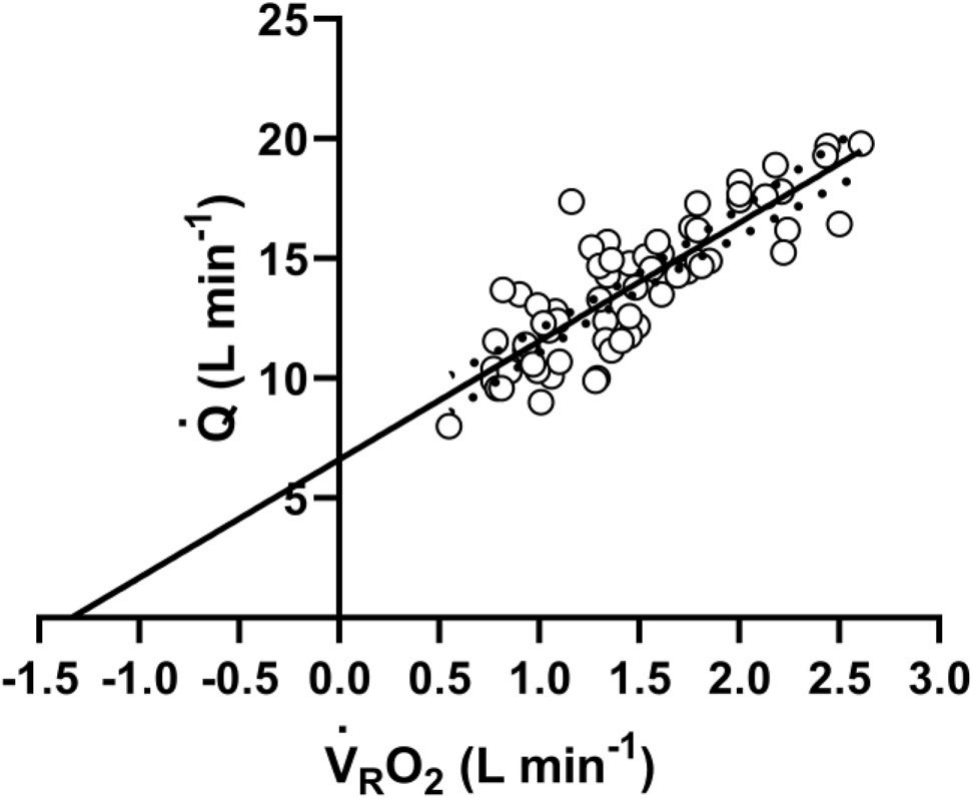
Cardiac output ($\dot{Q}$) as a function of oxygen flow (${\dot{V}}_{R}{O}_{2}$). All reported data, from various sources in the literature, were obtained during steady-state exercise in normoxia. The regression equation yields an *x*-axis intercept of − 1.35 L min^−1^, and a slope equal to 4.93 L of blood per L of oxygen, whence an oxygen flow in mixed venous blood of 1.35 L min^−1^ and an arterial oxygen concentration of 203 mL L^−1^. The continuous thick line corresponds to the regression equation; the dotted lines describe the confidence intervals [Modified after Adami et al. (2014)]

Equation (﻿31) implies that, if we plot $\dot{Q}$ as a function of ${\dot{V}}_{R}{O}_{2}$, we obtain a linear relationship (Fig. 10), with slope equal to ${{C}_{a}{O}_{2}}^{-1}$ and *x*-axis intercept equal to $-{\dot{Q}}_{\overline{v}}{O}_{2}$. Regression through the experimental data reported in Fig. 10 yielded a mean ${\dot{Q}}_{\overline{v}}{O}_{2}$ of 1.35 L min^−1^, whereas ${{C}_{a}{O}_{2}}^{-1}$ resulted equal to 4.93 L of blood per L of oxygen, yielding a theoretical ${C}_{a}{O}_{2}$ of 203 mL L^−1^. The slope of such a relationship should be higher in women than in men, due to the lower blood haemoglobin concentration in the former (Garry et al. 1954). Similarly, it should be increased in acute anaemia and decreased in acute polycythaemia.

**Fig. 11:**
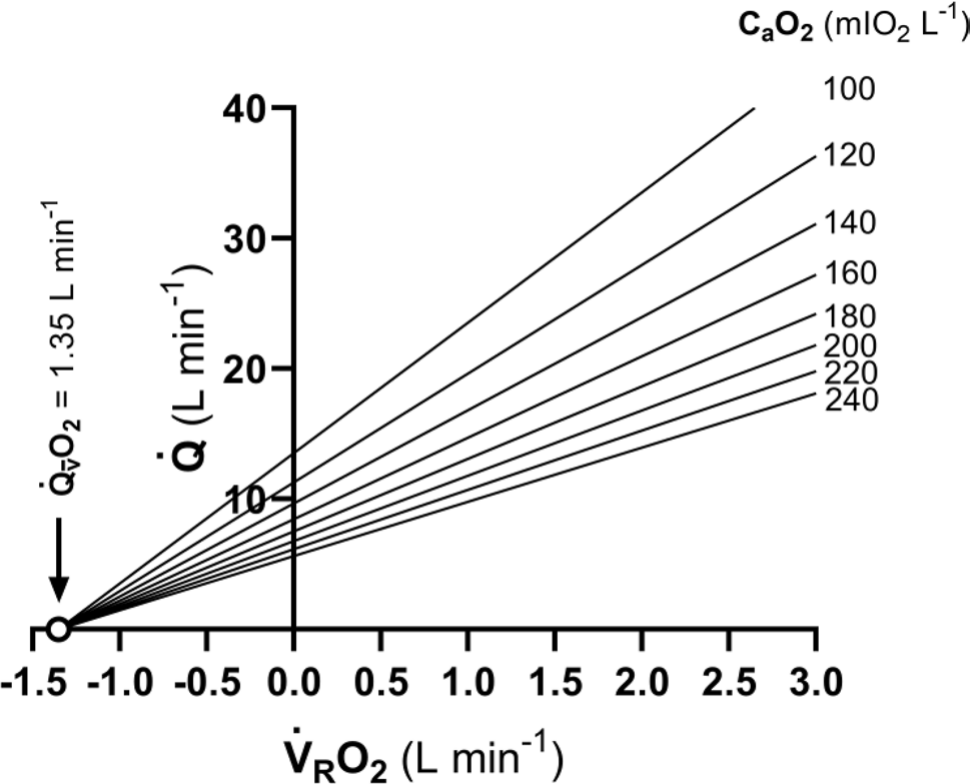
Theoretical representation of the relationship between cardiac output ($\dot{Q}$) and oxygen flow (${\dot{V}}_{R}{O}_{2}$) in exercising humans. Continuous lines are isopleths for the arterial oxygen concentration values (${C}_{a}{O}_{2}$, in mLO_2_ L^−1^) indicated on top right of each line. These lines converge on the same *x*-axis intercept (open dot) that permits to compute the value of the oxygen flow in mixed venous blood (${\dot{Q}}_{\overline{v}}{O}_{2}$, 1.35 L min^−1^)

Based on the data shown in Fig. 10, Adami et al. (2014) constructed a novel theoretical view of the steady-state cardiovascular responses to exercise as a function of the steady-state $\dot{V}{O}_{2}$ and ${C}_{a}{O}_{2}$, which they called the $\dot{Q}-{\dot{V}}_{R}{O}_{2}$ diagram. This view, expressed in graphical form in Fig. 11, is conceptually different from the classical $\dot{Q}$ versus ${\dot{V}}_{R}{O}_{2}$ relationship shown in Fig. 9. The latter figure, in fact, shows a progressive shift of the $\dot{Q}$ values toward isopleths for higher ${C}_{a}{O}_{2}-{C}_{\overline{v}}{O}_{2}$, as ${\dot{V}}_{R}{O}_{2}$ is increased: these isopleths converge on the origin of the axes (Cerretelli and di Prampero 1987). Conversely, Fig. 11 shows ${C}_{a}{O}_{2}$ isopleths converging on a negative *x*-axis intercept, corresponding to ${\dot{Q}}_{\overline{v}}{O}_{2}$. During moderate exercise in normoxia, the $\dot{Q}$ versus ${\dot{V}}_{R}{O}_{2}$ relationship coincides with one ${C}_{a}{O}_{2}$ isopleth, that for the incurring ${C}_{a}{O}_{2}$, because ${C}_{a}{O}_{2}$ is essentially invariant.

The data shown in Fig. 10 are exercise data, a condition where the activity of the parasympathetic branch of the autonomic nervous system is blunted, and thus remarkably lower than that of the sympathetic branch (Ekblom et al. 1972; Fagraeus and Linnarsson 1976; Robinson et al. 1966). At rest, in which there is predominant vagal control of heart rate (Malliani et al. 1991; Perini and Veicsteinas 2003), the data lie below those obtained at exercise, whence a lower ${\dot{Q}}_{\overline{v}}{O}_{2}$ value. This agrees with the previous observations, both during exercise on the cycle ergometer (Anchisi et al. 2001; Ferretti et al. 2005) and during two-legged knee extension exercise (Koskolou et al. 1997a; Roach et al. 1999). A lower ${\dot{Q}}_{\overline{v}}{O}_{2}$ implies a right shift of the ${C}_{a}{O}_{2}$ isopleths in Fig. 11 at rest (see Adami et al. 2014, Fig. 2 of that article).

As long as we operate on the flat part of the oxygen equilibrium curve, these relationships still endure. One example is anaemic individuals (Koskolou et al. 1997b; Roach et al. 1999; Woodson et al. 1978) who are characterised by a reduced ${C}_{a}{O}_{2}$, because they have a low blood haemoglobin concentration in absence of ${S}_{a}{O}_{2}$ changes. These subjects would have a $\dot{Q}$ versus ${\dot{V}}_{R}{O}_{2}$ relationship coinciding with a steeper ${C}_{a}{O}_{2}$ isopleth on the $\dot{Q}-{\dot{V}}_{R}{O}_{2}$ diagram. The same would occur with carbon monoxide poisoning (Gonzalez-Alonso et al. 2001). Another, opposite, example is polycythaemia, whatever its cause (Celsing et al. 1986; Ekblom et al. 1976; Ferretti et al. 1990, 1992), during which the $\dot{Q}$ versus ${\dot{V}}_{R}{O}_{2}$ relationship would coincide with a flatter ${C}_{a}{O}_{2}$ isopleth. We expect this to be the case also following erythropoietin administration, but the experiment is yet to be done. In the only study we are aware of, on the effects of erythropoietin administration on oxygen transport during moderate exercise (Thomsen et al. 2007), the authors did not measure $\dot{Q}$. All these changes are compatible with the inverse relationship between $\dot{Q}$ and ${C}_{a}{O}_{2}$ (Calbet et al. 2006).

Conversely, when we operate on the steep part of the oxygen equilibrium curve, as in acute hypoxia, diffusion limitation appears in alveolar–capillary oxygen transfer, depending on exercise intensity. In fact, the shortening of *t*_*c*_ induced by the increase in $\dot{Q}$ results in progressively lower ${S}_{a}{O}_{2}$ values at the arterial end of lung capillaries. Therefore, ${C}_{a}{O}_{2}$ in hypoxia becomes lower, the higher the ${\dot{V}}_{R}{O}_{2}$ and $\dot{Q}$. The progressive ${C}_{a}{O}_{2}$ reduction shifts the $\dot{Q}$ versus ${\dot{V}}_{R}{O}_{2}$ relationship toward an isopleth for lower ${C}_{a}{O}_{2}$ values. This implies that (i) the apparent $\dot{Q}$ versus ${\dot{V}}_{R}{O}_{2}$ line has a higher slope than that for normoxia; and (ii) the same line points toward a higher *x*-axis intercept, indicative of an apparently lower ${\dot{Q}}_{\overline{v}}{O}_{2}$ value (Adami et al. 2014). Anchisi et al. (2001) found lower ${\dot{Q}}_{\overline{v}}{O}_{2}$ the lower was the ${S}_{a}{O}_{2}$. They also found a positive linear relationship between these two parameters at exercise. This means that there is more than a mere displacement across ${C}_{a}{O}_{2}$ isopleths of the $\dot{Q}$ versus ${\dot{V}}_{R}{O}_{2}$ relationship in hypoxia. There is also a right displacement of the *x*-axis intercept, implying a decrease of ${\dot{Q}}_{\overline{v}}{O}_{2}$.

Various mechanisms may explain the characteristics of the $\dot{Q}-{\dot{V}}_{R}{O}_{2}$ diagram. Oxygen sensing mechanisms may be important in this context. Thus, it would be easy to think, above all, of the peripheral chemoreceptor: it is an oxygen sensor, inhibited by oxygen binding, with a clear transduction mechanism and afferent pathway, it has connections to the integrated cardio-respiratory control centres in the brainstem, and a dense autonomic innervation (Brognara et al. 2021; Lahiri et al. 2001; Patel and Honoré 2001; Spyer and Gourine 2009; Wilson and Teppema 2016). The carotid body would be an almost ideal oxygen sensor for a systemic response like that proposed by Ferretti et al. (1992). However, the observation that also local skeletal muscle blood flow responds to changes in ${C}_{a}{O}_{2}$ calls for mechanisms that may operate also in the periphery, not only on central circulation.

The haemoglobin molecule was then proposed as an oxygen sensor (Calbet 2000; Singel and Stamler 2005), with a mechanism potentially mediated by its S-nitrosylation, with subsequent rise of nitric oxide in blood, leading to peripheral vasodilation (Stamler et al. 1997). As stated by Premont et al. (2020), *hypoxic vasodilation is recapitulated by native S-nitrosothiol (SNO)-replete red blood cells and by SNO-haemoglobin itself, whereby SNO is released from haemoglobin and red blood cell during deoxygenation, in proportion to the degree of haemoglobin deoxygenation, to regulate vessels directly*. Crecelius et al. (2011a, b) proposed a synergistic effect of prostaglandins and nitric oxide in the regulation of peripheral blood flow during exercise in hypoxia. Alternatively, others have suggested that peripheral blood flow may be increased by ATP-mediated vasodilation in subjects with low ${C}_{a}{O}_{2}$ (Ellsworth et al. 1995; Gonzalez-Alonso et al. 2002; Mortensen et al. 2011). The mechanism proposed by Premont et al. (2020) is tempting, in so far as it transfers the sensing process to the level of local regulation of peripheral blood flow. However, if we admit such a mechanism, the inverse relationship between $\dot{Q}$ and ${C}_{a}{O}_{2}$ would be the non-deterministic result of a series of local actions on blood flow. It would make sense, rather, that a combination of central (carotid body stimulation) and peripheral (nitrosylation) mechanisms synergistically act to set the inverse relationship between $\dot{Q}$ and ${C}_{a}{O}_{2}$, and the constancy of ${\dot{Q}}_{\overline{v}}{O}_{2}$.

## On short-term pressure regulation

The physical laws governing the flow of a liquid in a pipe system, wherein flow is sustained by a pressure gradient that is necessary to overcome resistance to flow, apply to the cardiovascular system, as well. Therefore, at a first approximation, and neglecting the right atrial pressure, which is close to zero, $\dot{Q}$ is also equal to the ratio of the mean arterial pressure ($\overline{P }$) to ${R}_{p}$. Thus, Eq. (﻿5b) can be rewritten as follows:

32 $${\dot{V}}_{R}{O}_{2}=\frac{\overline{P}}{{R }_{p}} \left({C}_{a}{O}_{2}-{C}_{\overline{v}}{O}_{2}\right).$$

This means that, *ceteris paribus*, ${\dot{V}}_{R}{O}_{2}$ is directly proportional to $\overline{P }$ and inversely proportional to ${R}_{p}$. Therefore, keeping a tight coupling of respiration and metabolism is also a matter of arterial blood pressure. Without considering medium-term and long-term mechanisms of pressure regulation, in view of their longer time scale than that of the phenomena we are dealing with, short-term regulation of blood pressure may have an important role in maintaining adequate oxygen flow and blood flow to match metabolic demand.

The baroreflex system is the main short-term control system of arterial blood pressure. It includes low-pressure (atrial receptors) and high-pressure (arterial receptors) afferent inputs. The former have to do mainly with volume regulation. The latter consist essentially of the arterial baroreflexes. They can be conceptually represented as follows: the stretching of mechanical receptors, recently identified by some authors as piezo ion channels^8^ (Coste et al. 2012; Murthy et al. 2017; Retailleau et al. 2015; Wang et al. 2016), located in the arterial wall at the common carotid bifurcation and in the aortic arch, generates action potentials that are conveyed by the glossopharyngeal and the vagus nerve, respectively, to the cardiac and vasomotor centres, where they determine an integrated inhibitory response. Two components of this response have been identified: a cardiac component, acting through a reduction of ${f}_{H}$ mediated by vagal stimulation of the heart; and a vascular component, acting through inhibition of vascular sympathetic efferents. Both components lead to a reduction of $\overline{P }$ (Eckberg and Sleight 1992).

The concept of arterial baroreflexes was first proposed by Marey (1863), who described a possible reflex regulating arterial blood pressure on a short-time basis. However, the observation that both ${f}_{H}$ and arterial blood pressure increase at exercise, appeared in contrast with the reflex nature of pressure control (Krogh and Lindhard 1913b). Their interpretation was that the reflex was switched off at exercise by a neural mechanism. Two hypotheses were put forward to this aim: the first was the so-called exercise pressor reflex (Zuntz and Geppert 1886) and the second was the so-called central command hypothesis (Johansson 1895). Krogh and Lindhard (1913b) retained the latter, inasmuch as the activation of the cardiovascular system occurred simultaneously with the exercise onset.

**Fig. 12:**
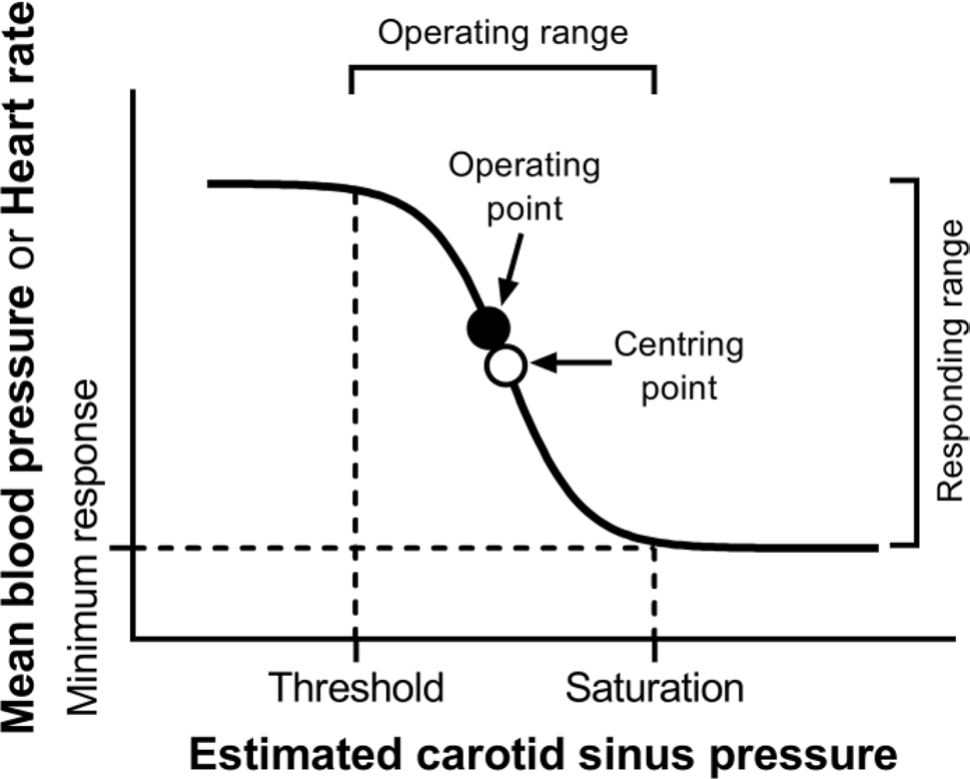
Schematic representation of the carotid baroreflex function curve and its operational parameters, based on the logistic model of Kent et al. (1972). The responding range corresponds to the overall change in the dependent variable. The operating range is the pressure difference between the carotid sinus pressures at the threshold (carotid sinus pressure below which no further changes in heart rate or in arterial pressure take place) and saturation (carotid sinus pressure above which no further drop in heart or in arterial pressure occurs) of the reflex. The operating point (prevailing pressure and heart rate at steady state before the stimulus) and the centring point (the point midway between the threshold and saturation pressures) are also shown. At rest, they are very close to each other. The maximal gain is the gain value at the centring point [Modified after Raven et al. (2006)]

The most significant development in human baroreflex representation, with respect to Krogh’s times, has been the introduction of the new concept of baroreflex resetting at exercise, which has replaced the concept of reflex switch-off. This change has introduced a new paradigm in our vision of baroreflexes at exercise. It was prompted by Bevegård and Shepherd (1966), who had been the first to use the neck suction–neck pressure technique on exercising humans. They showed that arterial baroreflexes were not switched off, but persisted during exercise, although in a different pressure range from rest. Moreover, that study set the basis for further investigations (Bristow et al. 1969, 1971; Cunningham et al. 1972; Pickering et al. 1971, 1972), which led Kent et al. (1972) to construct the logistic model of arterial baroreflexes. This model and its parameters are described in Fig. 12. The operating point corresponds to the prevailing $\overline{P }$ and ${f}_{H}$, around which the system operates at steady state. The maximal baroreflex gain is defined as the gain value at the centring point of a baroreflex curve (see Raven et al. 2006, for details).

**Fig. 13:**
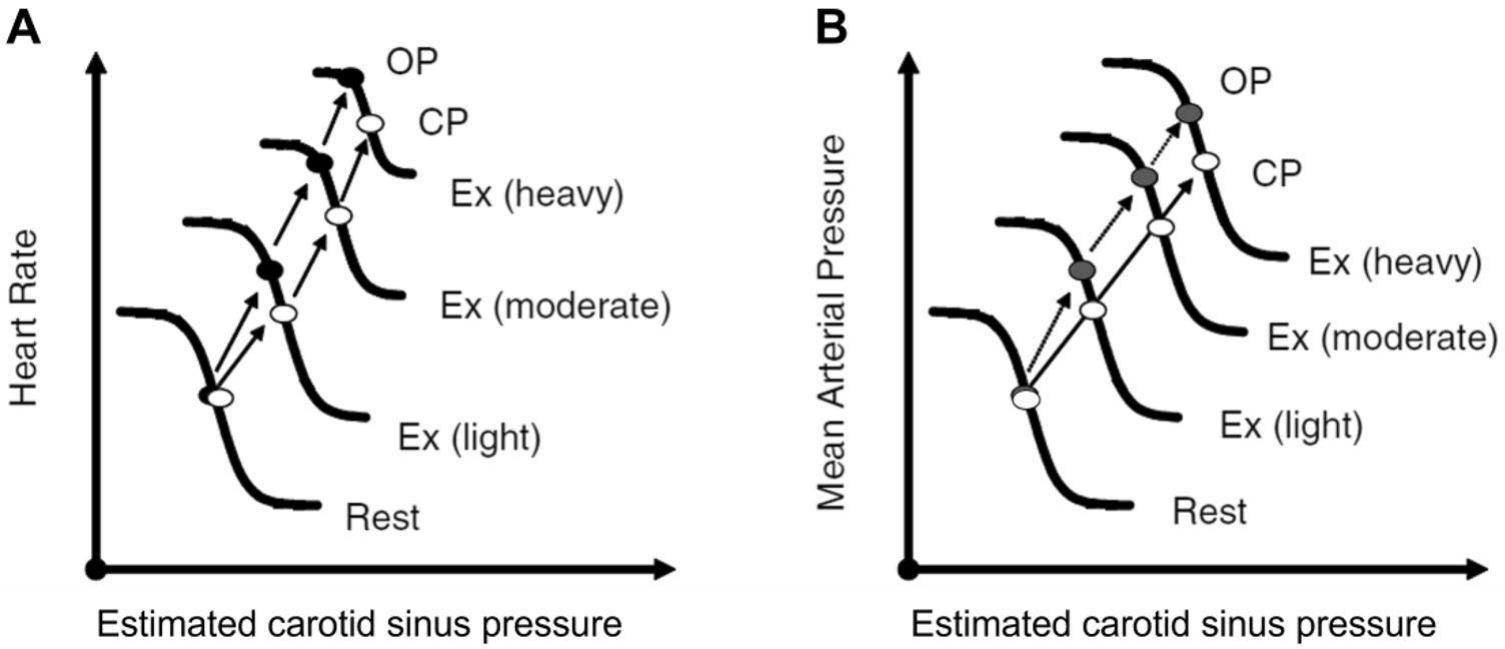
Representation of the carotid–cardiac (panel A) and carotid–vasomotor (panel B) baroreflex resetting, that occurs from rest to heavy exercise. It appears that (i) the entire baroreflex response curve at exercise is progressively displaced upward and rightward without changes in slope, and (ii) the operating point progressively shifts far from the centring point, toward the threshold pressure. *Ex* exercise in the indicated intensity domain, *CP* centring point, *OP* operating point [From Raven et al. (2006)]

The baroreflex curves at steady-state exercise were constructed in several studies, using the neck suction–neck pressure technique (Di Carlo and Bishop 1992; Norton et al. 1999a, b; Papelier et al. 1994; Potts et al. 1993). All those studies demonstrate that a displacement upward and rightward of the entire baroreflex curve occurs during exercise indeed: this displacement was defined as baroreflex resetting at exercise. Normally, baroreflex resetting occurs without changes in maximal gain, but with a shift of the operating point far from the centring point, toward the threshold pressure (Fadel and Raven 2012; Raven et al. 2006). A representation of baroreflex resetting during exercise is reported in Fig. 13. Baroreflex resetting was observed also in conditions, such as heat exposure (Crandall 2000), postural changes (Linnarsson et al. 2006; Ogoh et al. 2007), and simulated microgravity (Convertino et al. 1994; Linnarsson et al. 2006).

Despite the huge amount of work carried out since Krogh and Lindhard (1913b), the two hypotheses of a central command and of an exercise pressor reflex are still at the core of the debate on arterial baroreflexes as possible mechanisms behind baroreflex resetting at exercise (Fadel and Raven 2012; Fisher et al. 2015; Gallagher et al. 2006; McIlveen et al. 2001; Michelini et al. 2015; Raven et al. 2006, 2019). In fact these hypotheses are not mutually exclusive, and both were included in a comprehensive theory of baroreflex resetting at exercise (Degtyarenko and Kaufman 2006; Tsuchimochi et al. 2009).

The neck suction–neck pressure technique provides a static analysis of arterial baroreflexes, which requires a steady state in the cardiovascular system, upon which an external perturbation (open-loop approach) of varying intensity is introduced to modify the carotid distending pressure (independent variable); the ensuing ${f}_{H}$ or $\overline{P }$ responses are then recorded as dependent variables. This approach has led to thorough advancement in our baroreflex understanding, yet it has a limitation as to the understanding of the mechanisms behind resetting. Since it requires steady state, no dynamics of resetting can be studied with open-loop methods, despite awareness that resetting occurs during a dynamic condition, namely the transition between rest and exercise, or between two different exercise intensities. Nevertheless, baroreflex resetting in response to an external perturbation (exercise, for example) implies a transient phase, which occurs between the previous and the new steady state, and this was not accounted for in the open-loop approach. This is one of the most important reasons why the respective roles of central command and the exercise pressor reflex remain to be precisely defined. At present, the only chance of a deeper understanding of the mechanisms determining baroreflex resetting between two different steady states may come from an analysis of the temporal relationships during the dynamic phase of resetting. This analysis requires comprehensive studies of the kinetics of displacement of the baroreflex curve, or at least of its operating point, and therefore cannot be carried out with an open-loop approach.

Arterial baroreflexes can be investigated also by a different, less comprehensive way than the open-loop approach: the closed-loop analysis, wherein continuous beat-by-beat follow-up of the ${f}_{H}$ (or its reciprocal, the RR-interval, *RRi*) and blood pressure changes, assuming a continuous effect of blood pressure on ${f}_{H}$ with immediate counter-effect of ${f}_{H}$ on pressure. The most classical closed-loop method in the time domain is the sequence method (Bertinieri et al. 1988). This method is applied on sequences of at least three consecutive beats characterised by consensual variations of *RRi* and of arterial pressure. Within each sequence, Bertinieri et al. (1988) treated the *RRi* versus blood pressure relationship as linear, and assumed that the slope of this relationship represents the spontaneous baroreflex sensitivity around the operating point. It is noteworthy that, in conditions in which central blood volume, and thus mean arterial pressure, are increased, such as the supine posture (O’Leary et al. 2003; Steinback et al. 2005), water immersion (Chouchou et al. 2020), and short-term microgravity exposure (Di Rienzo et al. 2008; Hirayanagi et al. 2004), baroreflex sensitivity is also increased.

Conversely, application of the sequence method to the study of baroreflex sensitivity at rest and during exercise steady state has shown that the baroreflex sequences at exercise do not lie on an *RRi* versus pressure plot in the same position as at rest, but appear displaced downward and rightward, in agreement with the baroreflex resetting hypothesis (Bringard et al. 2017; Iellamo et al. 1994, 1997, 2002; Vallais et al. 2009). Moreover, the baroreflex sensitivity at exercise is remarkably reduced. The steady-state baroreflex curves reported in Fig. 13 show that the baroreflex operating point is displaced, along the logistic baroreflex curve, away from the centring point, toward the threshold pressure. This explains the finding of lower baroreflex sensitivity at exercise, as long as the slope of the logistic curve of Kent et al. (1972) becomes flatter as the threshold is approached.

Bringard et al. (2017) investigated the dynamics of baroreflex resetting with a closed-loop approach. Their data show that no baroreflex resetting was evident at exercise onset, as long as a progressive consensual increase in ${f}_{H}$ and in $\overline{P }$ up to the steady-state values appeared only after a minimum level of $\overline{P }$ had been attained, which occurred after some 10 s of exercise. In the first seconds of exercise, they found a linear negative segment of the ${f}_{H}$ and in $\overline{P }$ relationship, which they treated a sequence of heart beats, as defined by Bertinieri et al. (1988). The slope of this segment was equal to the baroreflex sensitivity during exercise, but significantly lower than the baroreflex sensitivity at rest. The initial linear segment appears as a baroreflex response to the sudden fall of $\overline{P }$ induced by muscle vasodilation at exercise onset, which occurs simultaneously with the first muscle contraction (Saltin et al. 1998). Conversely, the delayed onset of baroreflex resetting seems incompatible with the two hypotheses of central command and exercise pressor reflexes as its possible determinants. Notwithstanding, we cannot dismiss central command as a potential mechanism behind the reduction of baroreflex gain at exercise onset, in view of its immediacy. Similar conclusions were more recently attained by applying the same approach to the analysis of baroreflex changes during breath-holding (Taboni et al. 2021a) and during tilting (Taboni et al. 2021b).

## Matching things at steady state

After having analysed the main relationships that at steady state represent the cardio-respiratory responses to exercise, which are tightly coupled with the metabolic responses to exercise, in this section, we propose a synthesis of the various relationships that we have discussed in previous paragraphs. To do so, let us return to constant ${\dot{Q}}_{\overline{v}}{O}_{2}$, corresponding the *x*-axis intercept of a $\dot{Q}$ versus ${\dot{V}}_{R}{O}_{2}$ line, as outlined by Eq. (﻿31) and in Fig. 11. At the same time, the solution for $\dot{Q}$ of the ventilation–perfusion equation (Eq. ﻿22) is

33 $$\dot{Q}= -\frac{{\dot{V}}_{A}\; {P}_{A}{CO}_{2}} {{Cg}\;{RQ}_{L} \, \left({C}_{a}{O}_{2}-{C}_{\overline{v}}{O}_{2}\right)}.$$

Since Eqs. (﻿31) and (﻿33) must have equal solutions at steady state, we can also write

34 $$\frac{{\dot{V}}_{R}{O}_{2}+{\dot{Q}}_{\overline{v}}{O}_{2}}{{C}_{a}{O}_{2}}=- \frac{{\dot{V}}_{A}\; {P}_{A}{CO}_{2} }{{Cg}\; {RQ}_{L} \, \left({C}_{a}{O}_{2}-{C}_{\overline{v}}{O }_{2}\right)}.$$

Since

35 $${C}_{a}{O}_{2}=\left[{Hb}\right]\;\sigma\;{ S}_{a}{O}_{2},$$

where *σ* is the oxygen binding capacity of haemoglobin and $\left[{Hb}\right]$ is blood haemoglobin concentration^9^. Equation (﻿34) can thus be rewritten as follows:

36 $${\dot{V}}_{R}{O}_{2}+{\dot{Q}}_{\overline{v}}{O}_{2}=-\frac{\left[{Hb}\right]\;\sigma\; { S}_{a}{O}_{2}\; {\dot{V}}_{A}\; {P}_{A}{CO}_{2} }{{Cg}\;{RQ}_{L} \, \left({C}_{a}{O}_{2}-{C}_{\overline{v}}{O}_{2}\right)}.$$

For humans at sea level, since *Cg* = 0.863 mmHg and *σ* = 1.34 mL of oxygen per gram of haemoglobin, solving Eq. (﻿36) for ${\dot{V}}_{R}{O}_{2}$ would provide

37 $$\dot{V}{O}_{2}={\dot{V}}_{R}{O}_{2}=-\frac{1.552 \;\left[{Hb}\right]\; { S}_{a}{O}_{2}\; {\dot{V}}_{A}\; {P}_{A}{CO}_{2} }{{RQ}_{L} \, \left({C}_{a}{O}_{2}-{C}_{\overline{v}}{O}_{2}\right)}-{\dot{Q}}_{\overline{v}}{O}_{2}.$$

The most complete set of data reporting resting respiratory and cardiovascular values at sea level is the Operation Everest II, a huge study carried out in the 1980s wherein an ascension to Mount Everest was simulated in a hypobaric chamber and, among others, a systematic survey of the overall oxygen transport system was performed (Houston et al. 1987). At rest in normoxia, the subjects of Operation Everest II (Sutton et al. 1988) had ${P}_{A}{\mathrm{CO}}_{2}$ = 33.9 mmHg (arterial pH 7.43) and $\dot{V}{O}_{2}{=\dot{V}}_{R}{O}_{2}=$ 0.35 L min^−1^. For the other variables pertaining to Eq. (﻿37),^10^ we estimated: $\left[{Hb}\right]$ = 135 g L^−1^, ${S}_{a}{O}_{2}$ = 0.976, ${\dot{V}}_{A}$ = 5.97 L min^−1^ (calculated from ${\dot{V}}_{R}{CO}_{2}$, and ${P}_{A}{CO}_{2}$), ${RQ}_{L}$ = 0.771, ${C}_{a}{O}_{2}$ = 179 mL L^−1^, and ${C}_{\overline{v}}{O}_{2}$ = 122 mL L^−1^. This would have implied for them a ${\dot{Q}}_{\overline{v}}{O}_{2}$ of 0.50 L min^−1^, to be compared with a measured ${\dot{Q}}_{\overline{v}}{O}_{2}$ of 0.76 L min^−1^. Considering that the subjects of Operation Everest II had a low resting $\left[{Hb}\right]$ at sea level, which lowers the ${\dot{Q}}_{\overline{v}}{O}_{2}$ estimated after Eq. (﻿37), and a high resting $\dot{Q}$ (6.3 L min^−1^), which keeps their measured ${\dot{Q}}_{\overline{v}}{O}_{2}$ up, this is a remarkable correspondence; indeed, the discrepancy being essentially explained by interindividual data variability and by computation assumptions.

More generally speaking, Eq. (﻿36) defines how, in a steady-state condition, the characteristic variables describing the functional status of the oxygen transport system may adjust to variations of metabolic oxygen requirements, sustaining body homeostasis. Except for *σ* and for *Cg*, which are predefined constants independent of metabolism, many combinations of these variables are possible; only a few, in contrast, actually occur, depending on the settings of the various control mechanisms that operate in maintaining homeostasis. These settings are such as to result in a fairly stable ${\dot{Q}}_{\overline{v}}{O}_{2}$.

In normoxia, in which ${P}_{a}{CO}_{2}$(40 mmHg) and ${C}_{a}{O}_{2}$ (200 mL L^−1^ for normal $\left[{Hb}\right]$) may be considered essentially invariant during rest and steady-state submaximal exercise, any increase in $\dot{V}{O}_{2}$, requires an increase in both ${\dot{V}}_{A}$ and $\dot{Q}$. In fact, ${\dot{V}}_{A}$ is proportional to ${\dot{V}}_{R}{O}_{2}$ only if ${RQ}_{L}$ does not change, whereas $\dot{Q}$ is not proportional to $\dot{V}{O}_{2}$. In hypoxia, when diffusion limitation appears, both in lungs and in muscles (Piiper and Scheid 1981; Piiper 2000), ${C}_{a}{O}_{2}$ drops, $\dot{Q}$ only partially corrects this drop, the constant ${\dot{Q}}_{\overline{v}}{O}_{2}$ takes a lower value, and ${\dot{V}}_{A}$ goes up due to stimulation of peripheral chemoreceptors. All relations to ${\dot{V}}_{R}{O}_{2}$ are modified, and thus, the entire homeostasis of the respiratory system at exercise is modified: the new equilibrium is attained at ${P}_{a}{CO}_{2}$ values lower than 40 mmHg. We would expect a new equilibrium around lower ${P}_{a}{CO}_{2}$ values also when $b\dot{La}$ becomes positive and hyperventilation superimposes due to stimulation also of central chemoreceptors. This new steady state, however, is never attained, as we discuss below.

The next paragraphs will be devoted to analyse some major cases of rupture of steady-state homeostatic equilibria, implying potential disruption of the tight coupling of respiration and metabolism.

## Breaking the equilibrium: the exercise transient

When a human starts a constant-power exercise of moderate intensity, the resting equilibrium is broken and a transition between the resting and the exercise steady states takes place. Although the application of mechanical power is immediate, $\dot{V}{O}_{2}$, which at the steady state of aerobic exercise is equal to $\dot{E}$, increases at a remarkably slower rate. The steady-state $\dot{V}{O}_{2}$ is attained in approximately 3 min. During this transient phase, $\dot{V}{O}_{2}$ is insufficient to sustain the overall ATP resynthesis at the necessary rate to cope with the rate of ATP hydrolysis during muscular contraction. Therefore, metabolic power must be generated also by anaerobic metabolisms.

An energetic view of the $\dot{V}{O}_{2}$ kinetics upon exercise onset is discussed elsewhere (di Prampero 1981; Ferretti 2015). Here, we merely underline that, coherently with the principles set by Margaria et al. (1933), the $\dot{V}{O}_{2}$ kinetics was described as a mono-exponential

38 $${\dot{V}{{O}}_{2}}^{s}{-\dot{V}{{O}}_{2}}^{t}={\dot{V}{{O}}_{2}}^{s}{ e}^{-t\;{\tau }^{-1}},$$

where ${\dot{V}{O}_{2}}^{t}$ is the $\dot{V}{O}_{2}$ at time *t* during the transient, ${\dot{V}{O}_{2}}^{s}$ is the $\dot{V}{O}_{2}$ at steady state, and *τ* is the time constant describing the exponential equation. Within such a model, the overall oxygen deficit (${DO}_{2}$) can be computed as the time integral of Eq. (﻿38)

39 $${DO}_{2}={\int }_{0}^{\infty }{\dot{V}{O}_{2}}^{s}{ e}^{-t\;{\tau }^{-1}} dt= \tau \;{\dot{V}{O}_{2}}^{s}.$$

In Margaria’s energetic view, the oxygen deficit is the amount of energy derived from energy sources different from aerobic metabolism during the exercise transient.

The $\dot{V}{O}_{2}$ kinetics was generally determined from ${\dot{V}}_{R}{O}_{2}$ measurements at the mouth. This procedure implies the assumption that the kinetics of ${\dot{V}}_{R}{O}_{2}$ corresponds to the kinetics of $\dot{V}{O}_{2}$taking place in the working muscles, once possible changes in blood oxygen stores have been accounted for. This assumption carries along the corollary that the $\dot{V}{O}_{2} \,$ and ${\dot{V}}_{R}{O}_{2}$ kinetics have equal time constants, so that they remain tightly coupled also during the exercise transient. A second implicit fundamental assumption is that the muscle metabolic response to exercise onset follows the laws of linear first-order systems. Although this may indeed be the case during moderate exercise, it is definitely not so during higher intensity exercise. The energy balance of the $\dot{V}{O}_{2}$ kinetics based on these principles was originally analysed by di Prampero and Margaria (1968).

Several authors, however, considered this view too simplistic. The gap between the contracting muscles and the mouth is so wide, that it was hard to believe in a system as simple and linear as that proposed by di Prampero and Margaria (1968). Gilbert et al. (1967) were the first to suggest a rapid component of the ${\dot{V}}_{R}{O}_{2}$ kinetics at exercise onset. This finding was soon confirmed and analysed in detail (Whipp and Wasserman 1972). Wasserman et al. (1974), with experiments on anesthetized resting dogs, in which lung blood flow was artificially modified, demonstrated the possibility that the rapid component of ${\dot{V}}_{R}{O}_{2}$ changes in the exercise transient be due to a sudden increase in $\dot{Q}$. These findings suggested that a mono-exponential model might be insufficient to describe the ${\dot{V}}_{R}{O}_{2}$ kinetics at the onset of a square-wave exercise. The increasing evidence led to the creation of the double-exponential model with time delay of the ${\dot{V}}_{R}{O}_{2}$ kinetics (Barstow and Molé 1987). The arrival of this model questioned the tight matching of $\dot{V}{O}_{2}$ to ${\dot{V}}_{R}{O}_{2}$ during the exercise transient. In fact, the domain of application of the metabolic vision of the oxygen deficit was restricted to the second exponential only, which some authors later defined as the primary component of the ${\dot{V}}_{R}{O}_{2}$ kinetics.

It is interesting to note that, when Whipp and Wasserman (1972) analysed the rapid phase of the ${\dot{V}}_{R}{O}_{2}$ kinetics at exercise onset, several authors had already recognized a fast component in the dynamics of pulmonary ventilation (Asmussen 1973; Asmussen and Nielsen 1948; Åstrand and Christensen 1963; Comroe and Schmidt 1943; D’Angelo and Torelli 1971; Dejours 1964; Krogh and Lindhard 1913b). This generated the notion of dissociation of ventilatory control from metabolic control, inasmuch as the increase in ventilation was to precede the increase in $\dot{V}{O}_{2}$ (or in ${\dot{V}}_{R}{O}_{2}$). On this basis, many authors agreed that neurogenic mechanisms were behind the rapid ventilatory response at exercise onset, independent of whether a central command or a peripheral reflex mechanism was involved (Comroe 1944; Dejours 1964; Eldridge et al. 1981; Favier et al. 1983; McCloskey and Mitchell 1972; Turner 1991; Whipp 1994; Whipp and Ward 1982).

The question as to whether the time course of ${\dot{V}}_{R}{O}_{2}$ upon exercise onset is conveniently described by a two-phase, rather than a single-phase, ${\dot{V}}_{R}{O}_{2}$ kinetics, implying that the first rapid phase is dissociated from metabolic control, has been one of the fundamental questions in exercise physiology in the last 40 years (for a summary of the issue, see Poole and Jones 2012; Ferretti 2015). This question carries along another, directly related question, of much more pertinence in the present context. We can formulate it as follows: taking for granted that a rupture of a steady equilibrium occurs whenever we move from a steady state to a different steady state, implying a readjustment of most of the key parameters of Eq. (﻿36), is this readjustment fast enough to cope with the increasing oxygen demand? If it is, a tight coupling of respiration to metabolism is maintained even during the exercise transient; if it is not, this tight coupling is transiently broken, to be re-established when the new steady state has been attained. These two questions are relatively new. Originally, the oxygen deficit was analysed only from an energetic viewpoint, and it may be useful to start from an analysis of the energetics of the oxygen deficit also to answer the above questions.

## The energetics of the oxygen deficit

The interest of Margaria and his pupils was in understanding the question “where does the energy during the exercise transient come from”. Their answer was: from alactic and lactic anaerobic metabolisms. The former was considered an obligatory component of the oxygen deficit and was the starting point of the analysis of di Prampero and Margaria (1968); the latter appears in the intense exercise domain, to cover a gap between speed of activation of glycolysis, on one side, and of aerobic metabolism on the other (early lactate, Cerretelli et al. 1979).

They were also interested in the mechanism of the oxygen deficit. The main regulatory enzyme of glycolysis is phospho-fructo-kinase (PFK). As exercise starts, muscle ATP is immediately consumed in muscle contraction, so its concentration in contracting muscles tends to diminish. The first, rapid source of ATP resynthesis is the Lohmann reaction, the equilibrium constant of which is so elevated (between 20 and 100, depending on hydrogen ion and magnesium concentrations, see Carlson and Siger 1959; Kuby et al. 1954), that it prevents in fact a measurable ATP fall. PCr decreases and free creatine and Pi go up. As a consequence, PFK activity is accelerated. The energy flux along the glycolytic pathway increases and prompts the aerobic metabolism overall.

If, based on the preceding information, we assume that the metabolic aerobic pathway, and thus muscle $\dot{V}{{O}_{2}}$, is in some way controlled by the muscle PCr concentration, or, rather, by its mirror image, free creatine, the fraction of the alactic oxygen deficit related to PCr hydrolysis (${D}{O}_{2AL}$) controls muscle metabolism in the exercise transient (di Prampero and Margaria 1968; di Prampero et al. 2003; Mader 2003; Mahler 1985). This implies that the fraction of the oxygen deficit represented by ${D}{O}_{2AL}$ is a physiological necessity to cope with the increase of ATP consumption at the onset of muscle contraction: in the absence of the alactic oxygen deficit, the muscle could not increase its metabolism. In this vision, the physiological significance of the Lohmann reaction is brought to a higher level: PCr is not only the molecule yielding energy for muscular contraction instantaneously, but is also the key controller of aerobic metabolism (di Prampero 1981; Francescato et al. 2008; Greenhaff 2001; Meyer et al. 1984; Meyer 1988; Rossiter et al. 2005; Walsh et al. 2001; Whipp and Mahler 1980). This view is also supported by the experiments of Grassi and coworkers on the isolated–perfused muscle preparation. Grassi et al. (2011) concluded: “*The bioenergetic mechanism, which is fast enough to meet sudden increases in metabolic demand, that is PCr breakdown, is functionally related, through the level of some of its metabolites, to the regulation of oxidative phosphorylation, the most important mechanism for ATP resynthesis.*” For detail and further references on this and related matters, the reader is referred to Grassi et al. (2002, 2005).

**Fig. 14:**
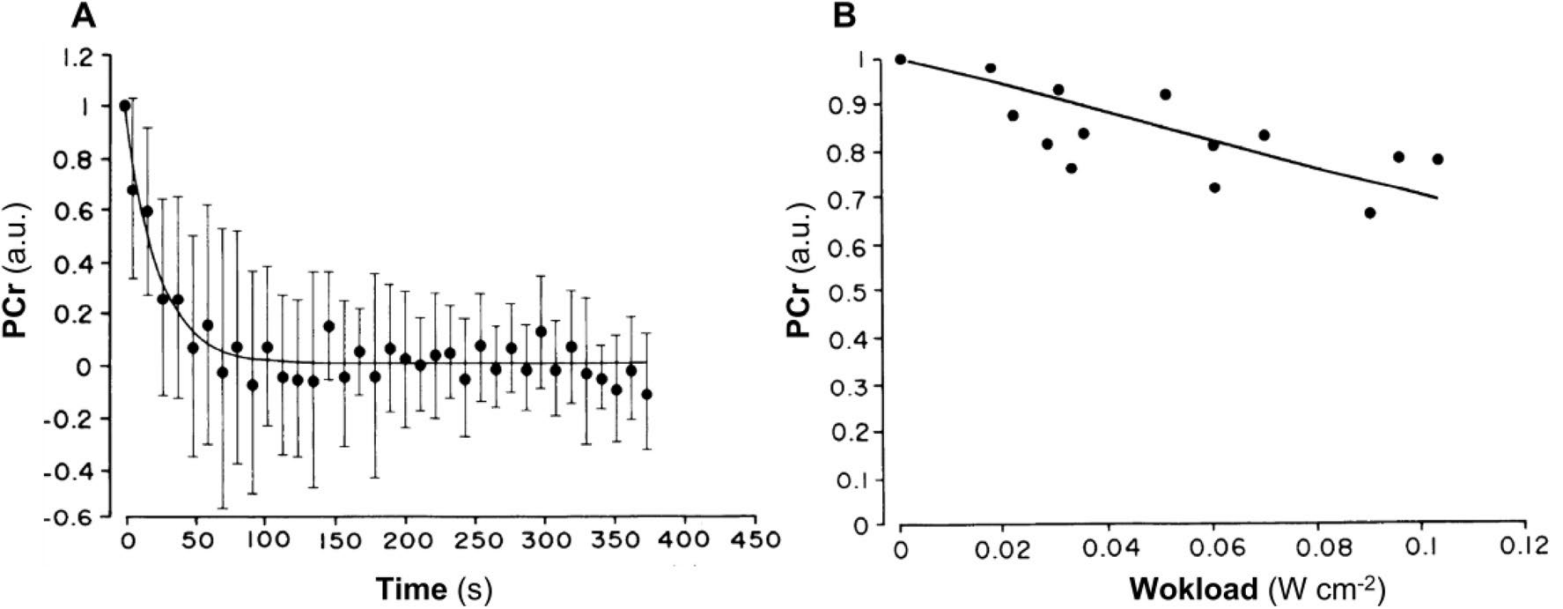
**A** time course of phosphocreatine concentration changes upon exercise onset, as determined by ^31^P nuclear magnetic resonance. The exponential function has a time constant of 23.4 s. Bars indicate standard deviation. **B** Linear relationship between steady-state phosphocreatine and power, from the same experiment [Modified after Binzoni et al. (1992)]

A necessary consequence of the vision of the oxygen deficit outlined above is that the muscle $\dot{V}{O}_{2}$ kinetics at the onset of contraction is a first-order kinetics, as proposed by di Prampero and Margaria (1968). This implies a linear relationship with negative slope between $\dot{V}{O}_{2}$ and PCr at steady state (Cerretelli et al. 1969; di Prampero and Margaria 1968). Such a relationship was originally demonstrated on the isolated–perfused muscle preparation during stimulation of various intensities (Piiper et al. 1968), and confirmed in humans by means of ^31^P-NMR (nuclear magnetic resonance) experiments (Binzoni et al. 1992). As a corollary of this linear relationship, note that the maximal anaerobic alactic power and capacity, since they depend on muscle PCr concentration, are lower the higher is the steady-state $\dot{V}{O}_{2}$ starting from which the test is performed (Margaria et al. 1971; Ferretti et al. 1987). Moreover, in the absence of early lactate accumulation in the contracting muscle mass, the kinetics of muscle PCr at exercise onset must have the same time constant as that of muscle $\dot{V}{O}_{2}$ (Binzoni and Cerretelli 1991). Several human studies using ^31^P-NMR spectroscopy, in which the latter time constant was calculated (Fig. 14), provided results coherent with both these predictions (Binzoni et al. 1992; di Prampero et al. 2003; Francescato et al. 2008, 2013; Rossiter et al. 1999, 2002; Whipp et al. 1999). Binzoni et al. (1992) reported a time constant (*τ*) for a mono-exponential PCr kinetics at exercise onset of 23.4 s. This value was substantially confirmed by the subsequent cited studies. Moreover, Behnke et al. (2002) obtained a *τ* of intramuscular $\dot{V}{O}_{2}$ kinetics of almost the exact same value, using phosphorescence quenching and intravital microscopy within contracting rat muscle.

The *τ* value of the PCr kinetics is also independent of the exercise intensity (Binzoni et al. 1997), so that the cumulative oxygen deficit of step-increasing powers up to a given level is equal to the oxygen deficit incurred if that very same level was attained in a single step. Moreover, the oxygen deficit is directly proportional to the steady-state $\dot{V}{O}_{2}$ with *τ* as proportionality constant, as shown by Eq. (﻿39). Finally, further experiments on the isolated-perfused muscle preparation showed that, when muscle blood flow is artificially increased at rest or rapidly accelerated at exercise onset, no reduction in the time constant of the kinetics of $\dot{V}{O}_{2}$ occurs (Grassi et al. 1998).

The muscle biochemical condition outlined here above typically corresponds to that of moderate aerobic exercise in normoxia, when no early lactate accumulation takes place and where body oxygen stores do not vary. The entire amount of energy released to build the oxygen deficit comes from PCr hydrolysis, as originally postulated by di Prampero and Margaria (1968). In this condition, the general equation of the energetics of muscular exercise (Eq. ﻿2) takes the following simplified form

40 $$\dot{E}\propto \overleftarrow{\dot{ATP}}= \overrightarrow{\dot{ATP}}={c}\dot{V}{O}_{2}+a \dot{PCr}.$$

During the exercise transient of a constant-power exercise, as long as $\dot{V}{O}_{2}$ increases, $\dot{PCr}$ decreases in such a way as to maintain the sum $c\dot{V}{O}_{2}$+ $a \dot{PCr}$ invariant. Within the context of a first-order kinetics system, and thus assuming a mono-exponential model of the $\dot{V}{O}_{2}$ increase inside the contracting muscles, at exercise time 0, all the $\dot{E}$ above the resting metabolic rate is from anaerobic alactic metabolism, so that Eq. (﻿2) becomes

41 $${\dot{E}}_{0}\propto \overleftarrow{\dot{ATP}}= \overrightarrow{\dot{ATP}}=a {\dot{PCr}}_{0},$$

where suffix 0 indicates the exercise time 0. This being so, then we have

42 $$a{\dot{PCr}}_{t}=a{\dot{PCr}}_{0} \; {e}^{-t\;{\tau }^{-1}},$$

where *t* is time and *τ* is equal to that of the $\dot{PCr}$ kinetics. The time integral of Eq. (﻿42) provides the overall amount of PCr hydrolysed during the exercise transient, constituting the oxygen deficit. This amount is directly proportional to the oxygen deficit, and thus to the applied mechanical power, whence the necessarily linear negative relationship between PCr concentration and $\dot{E}$.

A condition like that summarized in Eq. (﻿40) is met if, and only if, the *τ* of the primary component of the ${\dot{V}}_{R}{O}_{2}$ kinetics is equal to, or faster than, the *τ* of $\dot{V}{O}_{2}$ in the contracting muscle mass. If this condition is met, and here we come to the main point in the present context, the rupture of the equilibrium leading to the new steady state at a higher $\dot{V}{O}_{2}$ does not imply uncoupling of ${\dot{V}}_{R}{O}_{2}$ and $\dot{V}{O}_{2}$. And this is so, no matter whether the primary component of ${\dot{V}}_{R}{O}_{2}$ and the muscle $\dot{V}{O}_{2}$ are modulated by the same control system, or are under different yet parallel control systems. This means that, whenever ATP consumption is increased by contraction, and thus, PCr falls and Pi and creatine go up, and the aerobic metabolic pathway has accelerated its rate, thus requiring a higher $\dot{V}{O}_{2}$, the respiratory system has simultaneously responded in such a way as to adapt ${\dot{V}}_{R}{O}_{2}$ to the increased oxygen demand.

In fact, more recent studies have shown that the *τ* of the primary component of the ${\dot{V}}_{R}{O}_{2}$ kinetics, determined with Grønlund’s algorithm for breath-by-breath ${\dot{V}}_{R}{O}_{2}$ analysis (Capelli et al. 2001; Grønlund 1984), is faster (smaller) (Cautero et al. 2002, 2005; Lador et al. 2006, 2013) than that of the ${\dot{V}}_{R}{O}_{2}$ kinetics obtained in the classical studies (di Prampero 1981; Linnarsson 1974) carried out using the Auchincloss algorithm (Auchincloss et al. 1966), as a consequence of improved signal-to-noise ratio in breath-by-breath ${\dot{V}}_{R}{O}_{2}$ computation (Capelli et al. 2001). Therefore, it also turns out faster than that of muscle $\dot{V}{O}_{2}$ kinetics derived from muscle PCr kinetics (Binzoni et al. 1992; di Prampero et al. 2003; Rossiter et al. 1999), despite the fact that ${\dot{V}}_{R}{O}_{2}$ kinetics includes also the effects of changes in oxygen stores, which tend to increase *τ* and thus the corresponding oxygen deficit. Although the fast *τ* values obtained with Grønlund’s algorithm suggest that the ${\dot{V}}_{R}{O}_{2}$ kinetics may not correspond to the $\dot{V}{O}_{2}$ kinetics, this implies, neither that the energetic interpretation of the oxygen deficit is incorrect, nor that ${\dot{V}}_{R}{O}_{2}$ is unable to match peripheral oxygen demand. This means only that any quantitative analysis of the oxygen deficit performed on the basis of a ${\dot{V}}_{R}{O}_{2}$ kinetics analysed with Grønlund’s algorithm may provide an underestimate with respect to the oxygen deficit incurred in the contracting muscles, even accounting for the effects of oxygen store changes (Davies et al. 1972; di Prampero et al. 1970, 1983), which tend to increase the oxygen deficit. We maintain that, whenever the *τ* of the primary component of the ${\dot{V}}_{R}{O}_{2}$ kinetics is lower (faster) than, or at most equal to that of the $\dot{V}{O}_{2}$ kinetics, the tight coupling of ${\dot{V}}_{R}{O}_{2}$ ${\text{and}}$ $\dot{V}{O}_{2}$ subsists, and Eq. (﻿40) applies, even though the oxygen deficit computed from the former is actually smaller than that corresponding to the latter. Therefore, at least in the case of moderate aerobic exercise, breaking a steady-state equilibrium does not mean breaking the matching between respiratory oxygen flow and cell oxygen demand.

It is noteworthy that this line of reasoning certainly acknowledges that in the transient from rest to the steady state of moderate exercise, there is an increase in lactate production from glycolysis. Indeed, it is widely recognized that lactate production goes up whenever the glycolytic activity increases (see e.g. Ferguson et al. 2018; Poole et al. 2021). As Poole et al. (2021) clearly point out, during moderate exercise, the equilibrium between lactate production and lactate removal is maintained unaltered, since both occur at the same rate, even within the same muscle fibre.

**Fig. 15:**
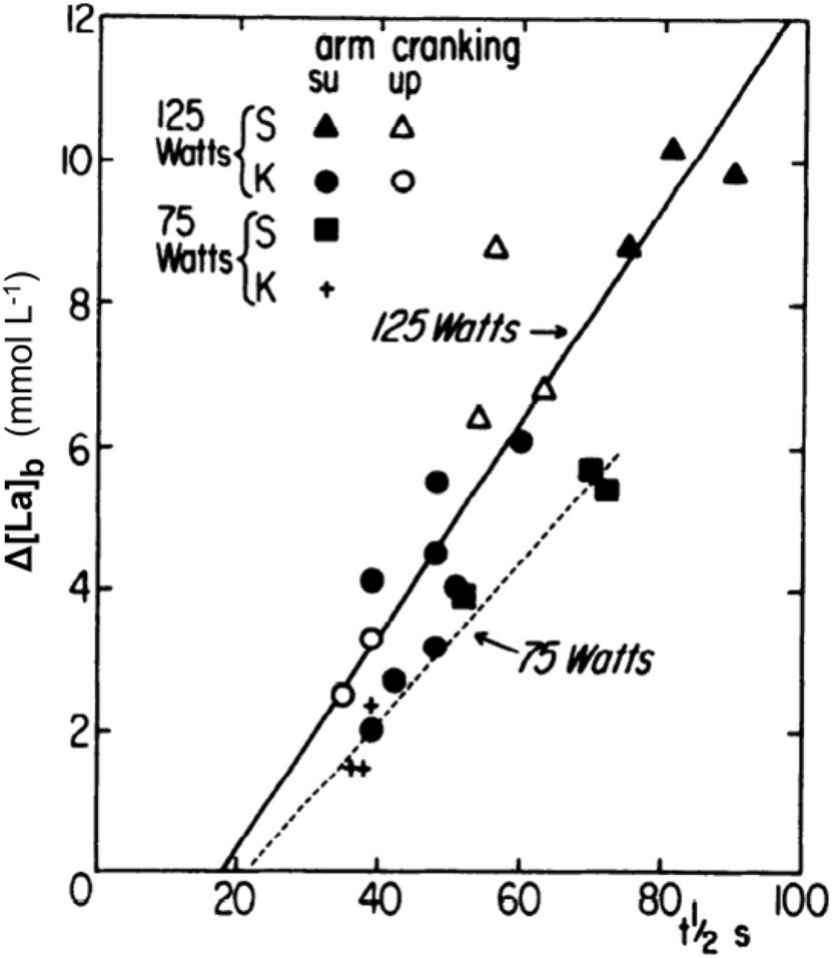
Net blood lactate accumulation (∆[*La*]_*b*_) at min 6 of square-wave exercises, as indicated on the graph, as a function of the half-time (*t*½) of the mono-exponential oxygen uptake increase upon exercise onset. Note that *t*½ = 0.69 *τ*. Note also that the representation proposed in this figure corresponds to the solution of Eq. (﻿43) for [*La*]_*e*_ (∆[*La*]_*b*_ in the figure). In this representation, the constant *τ*_0_ of Eq. (﻿43) corresponds to the *x*-axis intercept in the figure (yet expressed as *t*½ instead of *τ*). *S* sedentary, *K* kayakers [From Cerretelli et al. (1979)]

Notwithstanding, above a certain power, which may roughly correspond to the 50–60% of the maximal aerobic power, something different happens. According to Cerretelli et al. (1979), the *τ* of a mono-exponential $\dot{V}{O}_{2}$ increase at exercise onset becomes higher, the higher are the exercise intensity and $\dot{E}$. They showed that *τ* is linearly related to the amount of lactate that is accumulated in blood during the exercise transient, as described by

43 $$\tau =\lambda {\left[{La}\right]}_{e}+{\tau }_{0},$$

where [*La*]_*e*_ is early lactate (net blood lactate accumulation during the exercise transient), *λ* is a constant indicating how much *τ* increases per unit increase of lactate in blood during the exercise transient (di Prampero and Ferretti 1999), and *τ*_0_ is the time constant that would incur in the absence of early lactate accumulation. The constant *λ* is inversely proportional to the steady-state $\dot{V}{O}_{2}$. According to Cerretelli et al. (1979), at a steady-state $\dot{V}{O}_{2}$ of 1.5 and 1.0 L min^−1^ (arm cranking mechanical powers of 125 and 75 W), it is equal to 7.8 and 12.1 s mM^−1^, respectively (Fig. 15).

These values allow computation of the oxygen deficit increase (in mL O_2_ kg^−1^) per unit increase in [*La*]_*e*_ (in mM), which corresponds to the energy equivalent of blood lactate accumulation. This result is equal to 2.19 and 2.89 mL kg^−1^ mM^−1^ for the 125 and 75 W arm cranking power, respectively, the latter value agreeing particularly well with those reported in the literature (di Prampero 1981; di Prampero and Ferretti 1999). In turn, constant *τ*_0_ corresponds to the time constant describing Eq. (﻿42). Accounting for the error due to extrapolation of a regression equation, and to possible yet small changes in blood oxygen stores, the values of *τ*_0_ obtained by Cerretelli et al. (1979) (24.5 and 29.7 s for the two equations reported in Fig. 15) correspond well to those obtained for the PCr decrease from ^31^P-NMR studies (Binzoni et al. 1992; di Prampero et al. 2003; Francescato et al. 2008, 2013; Rossiter et al. 1999, 2002). When early lactate appears during an exercise transient, the overall oxygen deficit becomes larger than the net alactic oxygen deficit, so that *τ* becomes higher than that of PCr drop. An energy gap appears, bridged by early lactate. In the next paragraph, we discuss the hypothesis that this energy gap is created by a progressive slowing of the ${\dot{V}}_{R}{O}_{2}$ kinetics as the exercise intensity goes up. Discussing this hypothesis, however, requires a preliminary analysis of the $\dot{Q}$ kinetics.

## Kinetics of cardiac output and oxygen delivery at exercise onset

The first successful attempt at determining a kinetics of $\dot{Q}$ was not on a beat-by-beat basis (Cerretelli et al. 1966): this is perhaps why their kinetics of $\dot{Q}$ resulted slower than reported in subsequent studies. Even so, they realised that it was faster than that of ${\dot{V}}_{R}{O}_{2}$ and suggested that it may have a rapid initial increase, followed by a slower phase. They concluded that the discrepancy between the kinetics of $\dot{Q}$ and of ${\dot{V}}_{R}{O}_{2}$ demonstrates that the former is under neurogenic control. Davies et al. (1972) obtained similar results a few years later.

However, no kinetics of $\dot{Q}$ could be investigated on a beat-by-beat basis until the mid-1980s. Until then, only the kinetics of ${f}_{H}$ could be studied beat-by-beat in humans. The first ${f}_{H}$ data at exercise were obtained on rabbits in 1895, using pulse pressure recordings through a cannula placed in the carotid artery (Johansson 1895). Krogh and Lindhard (1913b) were the first to describe a ${f}_{H}$ kinetics at exercise onset in humans. Thanks to the continuous developments of ECG recording techniques, hundreds of studies can be found in the literature on the ${f}_{H}$ kinetics during exercise. Among these, a paper by Fagraeus and Linnarsson (1976) has gained great importance, for its impact on further thinking. These authors investigated the effects of parasympathetic blockade on the ${f}_{H}$ kinetics at exercise onset. The rapid response of ${f}_{H}$, which they identified in control condition, disappeared under atropine. This is the strongest evidence obtained so far supporting the hypothesis of vagal tone withdrawal at exercise onset.

As soon as beat-by-beat techniques for the determination of $\dot{Q}$ became available, it soon became clear that also the kinetics of $\dot{Q}$ at exercise onset is very fast, much faster than stated by Cerretelli et al. (1966) and much faster than that of $\dot{V}{O}_{2}$ (Adams et al. 1987; Cummin et al. 1986; De Cort et al. 1991; Eriksen et al. 1990; Magder et al. 1987; Yoshida and Whipp 1994). Moreover, Cummin et al. (1986) highlighted a close correspondence between the rapid increase of $\dot{Q}$ and of ventilation at exercise onset, suggesting the hypothesis of an overall integrated response of the respiratory system in the exercise transient. None of those authors, however, proposed a model of the $\dot{Q}$ kinetics at exercise onset.

Lador et al. (2006) were the first to model the kinetics of $\dot{Q}.$ In fact, they extended the domain of application of the dual exponential model of Barstow and Molé (1987) to describe also the kinetics of $\dot{Q}$. During moderate exercise, they obtained very low *τ* values not only for the initial rapid phase (phase I), but also for the second slower phase, or primary phase (phase II, *τ*_0_ equal to 2.1 s as compared to 15.4 s for $\dot{V}{O}_{2}$). Since ${C}_{a}{O}_{2}$ does not vary in normoxic exercise, the kinetics of ${\dot{Q}}_{a}{O}_{2}$ resulted practically superimposable to that of $\dot{Q}$. Application of a double-exponential model to the analysis of the kinetics of $\dot{Q}$, however, requires different interpretative hypotheses for each of the two phases of the $\dot{Q}$ response.

The rapid increase of $\dot{Q}$ in phase I is the consequence of the equally rapid increase of ${f}_{H}$ and of ${Q}_{s}$. For their interpretation of the rapid phase of the ${f}_{H}$ kinetics, Lador et al. (2006) relied on the vagal withdrawal hypothesis of Fagraeus and Linnarsson (1976). Coherently with this hypothesis, in hypoxia, when vagal activity is already reduced at rest, the amplitude of the ${f}_{H}$ increase in phase I was smaller than in normoxia (Lador et al. 2008). Further indirect support to this hypothesis was later provided by a study of heart rate variability at rest and exercise after parasympathetic blockade with atropine (Fontolliet et al. 2018). Recently, Fontolliet et al. (2021) demonstrated an almost complete suppression of phase I for ${f}_{H}$ under full vagal blockade with atropine.

Concerning the rapid increase in ${Q}_{s}$ (Faisal et al. 2009; Lador et al. 2006, 2008), a mechanical origin has been postulated. According to this hypothesis, a sudden increment in venous return may generate a rapid increase of ${Q}_{s}$ at the onset of exercise (Laughlin 1987; Leyk et al. 1994; Schneider et al. 2002; Sundblad et al. 2000). Similar mechanisms were proposed also to explain the $\dot{Q}$ increase in heart transplant recipients (Meyer et al. 1994) and patients carrying pacemakers for complete heart block (Nóbrega et al. 1997). Muscle pump action on venous blood flow by lower limb muscle contraction at exercise onset was identified as the possible mechanism enhancing venous return suddenly. Recently, the simultaneous determination of the kinetics of ${f}_{H}$, ${Q}_{s}$, and $\dot{Q}$ at exercise onset under various levels of exposure to lower body negative pressure provided strong support to this hypothesis (Fagoni et al. 2020). Yet, the mechanism whereby a phenomenon occurring in the right atrium (sudden increase in venous return) determines an immediate response by the left ventricle (sudden increase in ${Q}_{s}$) is still poorly understood. The interaction of several mechanisms has been evoked: the Frank–Starling mechanism, ventricular interdependence, with pulmonary circulation acting as a buffer mitigating the phenomenon (Chung et al. 1997; Naeije and Badagliacca 2017; Sundblad et al. 2000, 2014). A different track was taken by Elstad et al. (2009), who highlighted the role of muscle vasodilation and the subsequent *R*_*p*_ fall in determining the rapid ${Q}_{s}$ response. According to them, the ensuing decrease in $\overline{P }$ would be the main determinant in the ${Q}_{s}$ dynamics. Whatever mechanism is operant, the putative cause of the phase I response of ${Q}_{s}$ may well differ from the neural mechanism acting on ${f}_{H}$, both coexisting and acting simultaneously to determine the rapid $\dot{Q}$ response (phase I) in healthy humans.

The phase I $\dot{Q}$ response is associated with an extremely fast, functionally instantaneous, phase I of the ${\dot{V}}_{R}{O}_{2}$ kinetics (Lador et al. 2006, 2008; Faisal et al. 2009). This implies an immediate upward translation of ${\dot{V}}_{R}{O}_{2}$_,_ compatible with the observation (Cummin et al. 1986) of a close correspondence between the phase I increase of $\dot{Q}$ and ventilation at exercise onset. It provides also further support to the hypothesis (Wasserman et al. 1974; Whipp and Ward 1982) of the cardiac origin of the phase I ${\dot{V}}_{R}{O}_{2}$ increase at exercise onset. Because of a delay between $\dot{V}{O}_{2}$ and ${\dot{V}}_{R}{O}_{2}$, we can assume that in the first seconds of exercise, the gas composition of mixed venous blood remains unchanged, so that ${C}_{a}{O}_{2}-{C}_{\overline{v}}{O}_{2}$ is the same as at rest (Barstow and Molé 1987; Weissman et al. 1982). If this is so, and were the $\dot{V}{O}_{2}$ increase in phase I due only to an increase in $\dot{Q}$, especially in the pulmonary circulation, due to a sudden increase in venous return, application of the Fick principle to lung blood flow in phase I should provide a theoretical ${\dot{V}}_{R}{O}_{2}$ increase equal to the measured amplitude of the ${\dot{V}}_{R}{O}_{2}$ increase in the same phase. This analysis was performed by Lador et al. (2006), who found an average amplitude of the $\dot{Q}$ response in phase I equal to 4.3 L min^−1^. For an average resting ${C}_{a}{O}_{2}-{C}_{\overline{v}}{O}_{2}$ of 87 mL L^−1^, this would carry along a corresponding immediate $\dot{V}{O}_{2}$ increase of 374 mL min^−1^, which is very close to the observed amplitude of phase I for ${\dot{V}}_{R}{O}_{2}$, reported in the same study (355 ± 148 mL min^−1^), as was to be demonstrated.

Lador et al. (2006) found also faster phase II *τ* of $\dot{Q}$ and ${\dot{Q}}_{a}{O}_{2}$ than of $\dot{V}{O}_{2}$, with equal time delay. Thus, the amount of oxygen made available to the contracting muscle mass in phase II exceeded the oxygen demand. This caused a transient decrease of ${C}_{a}{O}_{2}-{C}_{\overline{v}}{O}_{2}$, with consequent transient increase of the oxygen stores in venous blood. They attributed the phase II increase in $\dot{Q}$ to the progressive activation of sympathetic heart stimulation. Under this condition, even though strictly speaking the performance of the respiratory system exceeds that of muscle metabolism, thus demonstrating an imperfect coupling of respiratory oxygen flow and muscle oxygen demand, the response of the respiratory system in coping with oxygen demand is anyway adequate. Thus, oxidative phosphorylation is accelerated at the necessary rate to follow the kinetics of activation of glycolysis: no early lactate accumulation occurs and Eq. (﻿40) applies, as occurs in case of adequate coupling of respiration to metabolism.

## The $\dot{V}{O}_{2}$ kinetics in hypoxia

The primary component (phase II) of the $\dot{V}{O}_{2}$ kinetics in hypoxia shows a higher (slower) *τ*_2_ than in normoxia (Cleuziou et al. 2005; Engelen et al. 1996; Hughson and Kowalchuk 1995; Lador et al. 2008, 2013; Springer et al. 1991), as does the phase II kinetics of $\dot{Q}$ and ${\dot{Q}}_{a}{O}_{2}$ (Lador et al. 2008, 2013). Only one study, however, reported at the same time the values of early lactate accumulation (Lador et al. 2013): we therefore refer to that study for the present analysis.

The average cumulative volume of oxygen (${V}_{L}{O}_{2}$) moving across the alveolar–capillary barrier during a constant-load exercise is given by

44 $${V}_{L}{O}_{2} \, \text{=}{\int }_{0}^{t}d{\dot{V}}_{L}{O}_{2}\; dt.$$

The simultaneous changes in venous blood oxygen stores (*∆V*_*v*_*O*_2_) occurring during the exercise transient are equal to (Barstow et al. 1990)

45 $$\Delta {V}_{v}{O}_{2}=\left[{\left(\frac{\dot{{V}}{{O}}_{2}}{\dot{Q}}\right)}_{ss}-{\left(\frac{\dot{V}{O}_{2}}{\dot{Q}}\right)}_{r}\right] \cdot {V}_{v},$$

where suffixes *ss* and *r* indicate the steady-state exercise and resting conditions, respectively, and *V*_*v*_ is the venous blood volume, that Barstow et al. (1990) assumed invariant within the exercise transient and equal to 3 L. On this basis, the results of Lador et al. (2013) yield a *∆V*_*v*_*O*_2_ equal to 260 and to 230 mL in normoxia and hypoxia, respectively. If we add these values to ${VO}_{2}$, we obtain the volume of oxygen extracted by the contracting muscles (${VO}_{2{M}}$) in the two conditions. In constant-power exercise, the muscular oxygen deficit (${DO}_{2{M}}$) is equal to (di Prampero 1981; di Prampero and Ferretti 1999)

46 $${DO}_{2{M}}=\dot{E}\; t-{VO}_{2{M}}=\dot{V}{O}_{2} \; t-{VO}_{2{M}},$$

where *t* is the exercise time. Since the changes in blood oxygen stores are accounted for in the definition of ${VO}_{2{M}}$, we also have

47 $${DO}_{2{M}}={DO}_{2{AL}}+{DO}_{2{LA}},$$

where ${DO}_{2{LA}}$ is the facultative component of ${DO}_{2{M}}$, represented by [*La*]_*e*_.

As already pointed out, in homogeneous aerobic conditions, i.e., when ${DO}_{2{LA}}$ is nil, ${DO}_{2{M}} \, = {DO}_{2{AL}}$ and the *τ* of muscle $\dot{V}{O}_{2}$ is equal to that of the mono-exponential PCr decrease (Binzoni and Cerretelli 1991), ranging between 20 and 25 s. In the study of Lador et al. (2013), the homogeneous aerobic condition is represented by the experiments in normoxia: no increase in blood lactate was observed during the exercise transient. Therefore, the estimated *τ* of muscle $\dot{V}{O}_{2}$ kinetics corresponded well to that reported for muscle PCr kinetics. It is noteworthy that in this case, the phase II *τ* of $\dot{Q}$ and ${\dot{Q}}_{a}{O}_{2}$, and thus of ${\dot{V}}_{R}{O}_{2}$, were faster than that of $\dot{V}{O}_{2}$.

Things are not so in hypoxia, as long as ${DO}_{2{M}}$ was larger than in normoxia and [*La*]_*e*_ was positive. Since in Lador et al. (2013), the applied mechanical power was the same in hypoxia as in normoxia, and so was the steady-state $\dot{V}{O}_{2}$, ${DO}_{2{AL}}$ was necessarily equal in both conditions. Therefore, the increase in ${DO}_{2{M}}$ in hypoxia was exclusively due to the increase in ${DO}_{2{LA}}$. The energy provided by ${DO}_{2{LA}}$ in hypoxia must be equal to the difference in ${DO}_{2{M}}$ between hypoxia and normoxia. The ratio between ${DO}_{2{LA}}$ and [*La*]_*e*_ (1.3 mM in Lador et al. 2013), expressed per unit of body mass, turns out equal to 2.5 mL O_2_ mM^−1^ kg^−1^, a value fairly close to the range normally admitted for the energy equivalent of blood lactate accumulation (di Prampero 1981; di Prampero and Ferretti 1999). The energy balance fits. It is noteworthy, however, that in this case, the *τ*_2_ of $\dot{Q}$ and ${\dot{Q}}_{a}{O}_{2}$, and thus of ${\dot{V}}_{R}{O}_{2}$, were slower than that of $\dot{V}{O}_{2}$.

## Matching things in the exercise transient

### Equilibrium below the maximal lactate steady state

To sum up, whenever the phase II kinetics of $\dot{Q}$ and ${\dot{Q}}_{a}{O}_{2}$, and thus of ${\dot{V}}_{R}{O}_{2}$, are faster than that of $\dot{V}{O}_{2}$, despite the rupture of the steady-state condition, a tight matching of ${\dot{V}}_{R}{O}_{2}$ to $\dot{V}{O}_{2}$ is maintained. However, as soon as the rate, at which $\dot{Q}$ and ${\dot{Q}}_{a}{O}_{2}$, and thus ${\dot{V}}_{R}{O}_{2}$, increase in an exercise transient, becomes as slow as or slower than the rate at which $\dot{V}{O}_{2}$ increases, then the tight coupling of ${\dot{V}}_{R}{O}_{2}$ to $\dot{V}{O}_{2}$ is also broken, as is the tight coupling of respiration and metabolism. This occurs anytime the homogeneous aerobic condition is broken, around the so-called lactate threshold. In this case, although muscle blood flow may be able to cope with the energy demand, as suggested by some studies (Behnke et al. 2001; Grassi et al. 1996), the ${\dot{V}}_{R}{O}_{2}$ kinetics, and thus the muscle $\dot{V}{O}_{2}$ kinetics, become slower than the kinetics of PCr decrease, and the glycolytic pathway is accelerated at a faster rate than oxidative phosphorylation. When this occurs, pyruvate accumulates in contracting muscle fibres, especially type II muscle fibres, thus promoting muscle, then blood lactate accumulation. If the power, though higher than that ensuring [*La*]_*e*_ = 0 mM, is low enough to allow the contracting muscle mass to operate in a heterogeneous aerobic condition (di Prampero and Ferretti 1999), a new equilibrium at a new apparent steady state is attained at a higher steady lactate concentration than that at rest. This lactate concentration may even be higher than 5 mM (Ribeiro et al. 1986): its highest value was defined as the maximal lactate steady state (Beneke 2003). At higher workloads, the exercise can go on for a time (limit time, *t*_lim_) that is shorter the higher is the applied mechanical power.

The concept of maximal lactate steady state is strictly related to the concept of even or uneven, or homogeneous and heterogeneous, aerobic metabolism (Antonutto and di Prampero 1995; di Prampero and Ferretti 1999). During moderate exercise, all active muscle fibres (essentially type I slow fibres) operate in homogeneous aerobic conditions, which means that each fibre is able to complete the entire pathway from glucose to oxygen consumption within itself. In this case, the oxygen deficit consists only of its obligatory component, and muscle PCr kinetics is a mirror image of muscle $\dot{V}{O}_{2}$ kinetics.

Increasing power, and thus $\dot{E}$, carries along the recruitment of a larger number of motor units, and thus of muscle fibres. Also type IIb fibres, with lower mitochondrial volume and oxidative potential than aerobic type I fibres, need to be activated. The maximal $\dot{V}{O}_{2}$of the former fibres is lower than that of the latter fibres, so that those fibres start accumulating lactate (hypoaerobic condition), while these fibres do not yet. This phenomenon may be accentuated by the progressive slowing of the kinetics of cardiovascular variables, which reduces the amount of oxygen relative to the energy demand that is provided to the contracting muscle mass during the exercise transient. Therefore, muscle lactate concentration increases. This tendency is counteracted, however, by the ability of type I fibres, which still have the possibility to increment their aerobic metabolism (hyperaerobic fibres), to take up lactate produced by adjacent type II fibres. This carries along a new equilibrium, whereby steady blood lactate concentration higher than at rest can be maintained. This condition, defined as unevenly aerobic condition (di Prampero and Ferretti 1999), is tantamount to the lactate shuttle concept (Brooks 1985, 2018; Brooks et al. 1999), is the source of [*La*]_*e*_, and allows re-establishment of an equilibrium between ${\dot{V}}_{R}{O}_{2}$ and $\dot{V}{O}_{2}$. Nevertheless, this equilibrium is fragile, precarious. The appearance of the so-called “slow component” of the ${\dot{V}}_{R}{O}_{2}$ kinetics (Camus et al. 1988; Paterson and Whipp 1991; Poole et al. 1988; Whipp and Wasserman 1972) pertains to this exercise domain. For a more detailed discussion of the physiological and energetic meaning of the slow component, we address the reader to more specific references (Jones et al. 2011; Poole and Jones 2012; Poole et al. 1994).

### Disequilibrium above maximal lactate steady state or critical power

If we further increase the exercise intensity, no form of equilibrium can be attained anymore. Both the metabolic rate of type I fibres and the lactate production by type IIb fibres increase. Although the power is still below the individual maximal aerobic power, muscle and blood lactate concentrations keep increasing with time. Muscle pH drops. Type IIb fibres fatigue and their efficiency decrease. Type I fibres become unable to oxidize also the lactate produced by type IIb fibres. The amplitude of the slow component becomes larger, ${\dot{V}}_{R}{{O}}_{2}$ increases until it reaches its maximum and exercise is then terminated (Poole et al. 1988). At even higher powers, also type I fibres attain their maximal $\dot{V}{O}_{2}$: we enter the supramaximal exercise domain, all active muscle fibres produce lactate, the rate of blood lactate accumulation, and thus, the term $b \dot{La}$ of Eq. (2) (anaerobic lactic power, as defined by Margaria et al. 1963), becomes higher and higher as the applied mechanical power is further increased. Blood homeostasis is under attack, the massive stimulation of central chemoreceptors determines a strong hyperventilation, but the rate of blood lactate accumulation overrides the capacity of controlling pH. Muscle pH drops dramatically and glycolysis is inhibited: exercise must be stopped. When the maximal lactic power has been attained (Margaria et al. 1964), this occurs in less than 1 min after the exercise onset.

The relationship between *t*_lim_ and power in this intense exercise domain is hyperbolic, as described by the critical power model (Jones et al. 2010; Monod and Scherrer 1965), and more precisely by the three-parameter critical power model (Morton 1996), which includes also extreme exercise intensities (Vinetti et al. 2019), up to the maximal lactic power. The critical power models clearly indicate that the new equilibrium here is not achievable, affecting maximal exercise duration.

The homeostatic equilibrium of the milieu intérieur, totally upset in supramaximal exercise, is re-established in the recovery phase after the end of exercise, including the return to a tight match between respiration and metabolism. The timing of functional recovery, which was precisely described already by Margaria et al. (1933), depends on the kinetics of lactate washout by oxidation to pyruvate, which then feeds the Krebs’ cycle, in the muscle fibres as well as in other organs. The kinetics of lactate removal is accelerated if moderate exercise is performed in the recovery period (Belcastro and Bonen 1975; Gisolfi et al. 1966; Reaburn and McKinnon 1990).

## Breaking the equilibrium: breath-holding

Breath-holding implies a sudden shift to ${\dot{V}}_{A}$ = 0 L min^−1^, so that the ${\dot{V}}_{A}/{\dot{V}}_{R}{CO}_{2}$ immediately jumps from − 21.6 to 0. This represents a dramatic rupture of the homeostatic equilibria in the respiratory system, determining abrupt unsteady state and uncoupling of respiration from metabolism within seconds. Since no gas exchange between ambient air and alveolar air occurs anymore, ${P}_{A}{O}_{2}$ and ${P}_{a}{O}_{2}$ fall impressively, whereas ${P}_{A}{CO}_{2}$ does not increase that much, only because most of the carbon dioxide produced by metabolism is accumulated in tissues. Yet the combination of hypoxia and hypercapnia leads rapidly to strong respiratory stimuli, which cause first diaphragmatic contractions, then the interruption of breath-holding. The catastrophe of the milieu intérieur is so dramatic, that the interruption of breath-holding at rest, even if performed starting from a lung volume equal to the total lung capacity, normally intervenes within 3 min (Ferretti 2001; Lindholm and Lundgren 2009). And the uncoupling of respiration and metabolism, accentuated by the appearance of a diving response even in humans, is not the most serious event under these circumstances, although it is the subject of this review. For a more general overview of the physiology of breath—holding and breath—hold diving, we address the reader to other, more specific, review articles (Bain et al. 2018; Elia et al. 2021c; Ferretti 2001; Fitz-Clarke 2018; Lindholm and Lundgren 2009; Pendergast and Lundgren 2009).

Serial analyses of alveolar gas composition at the end of breath-holds of increasing duration have led to set the limits of voluntary breath-holding at the boundaries of the anoxic collapse and of the hypercapnic narcosis regions on an O_2_–CO_2_ diagram (Ferretti et al. 1991; Lin et al. 1974). The attainment of these limits follows alveolar pathways during breath-holding, which have been precisely analysed over 5 decades ago (Agostoni 1963; Fagoni et al. 2017; Ferretti et al. 1991; Lanphier and Rahn 1963; Lin et al. 1974; Lindholm and Lundgren 2006; Otis et al. 1948; Taboni et al. 2019).

Oxygen has a much lower solubility in blood and cells than carbon dioxide (respectively, 0.0214 and 0.515 mL of gas per millilitre of human plasma at sea level at 37 °C; Christmas and Bassingthwaighte 2017), so that carbon dioxide storage capacity is some 25 times larger than oxygen storage capacity. Blood and lungs are mostly involved, with small capacity and thus fast equilibrium time constants (Farhi and Rahn 1960; Linér and Linnarsson 1994). Thus, the amount of carbon dioxide that goes into rapidly exchanging tissues at the beginning of a hypoventilation is by far larger than the amount of oxygen that moves out of the same tissues, before a new equilibrium is attained.

These are the reasons why the patterns followed by alveolar gases in the unsteady state at the beginning of hypoventilation reproduce what we call a hypoventilation loop (Farhi and Rahn 1955; Rahn and Fenn 1955). The curvilinear segment representing a hypoventilation loop on the O_2_–CO_2_ diagram is concave, starts and ends on the prevailing steady-state ${RQ}_{L}$ line, and follows a pattern moving leftward to it. This segment is larger, the closer to zero is the ${\dot{V}}_{A}/{\dot{V}}_{R}{CO}_{2}$ during hypoventilation.

Notwithstanding, validation of the hypoventilation loop concept has been rarely performed. The most complete study is still that by Rahn and Otis (1949), who induced acute alveolar hypoventilation by artificially increasing the anatomical dead space by adding a hose between the mouth and the breathing valve. They found that the alveolar pathway initially moved leftward and then upward on the O_2_–CO_2_ diagram. Farhi and Rahn (1955) later proposed that the alveolar pathway of ventilatory unsteady states can be described by exponential functions.

**Fig. 16:**
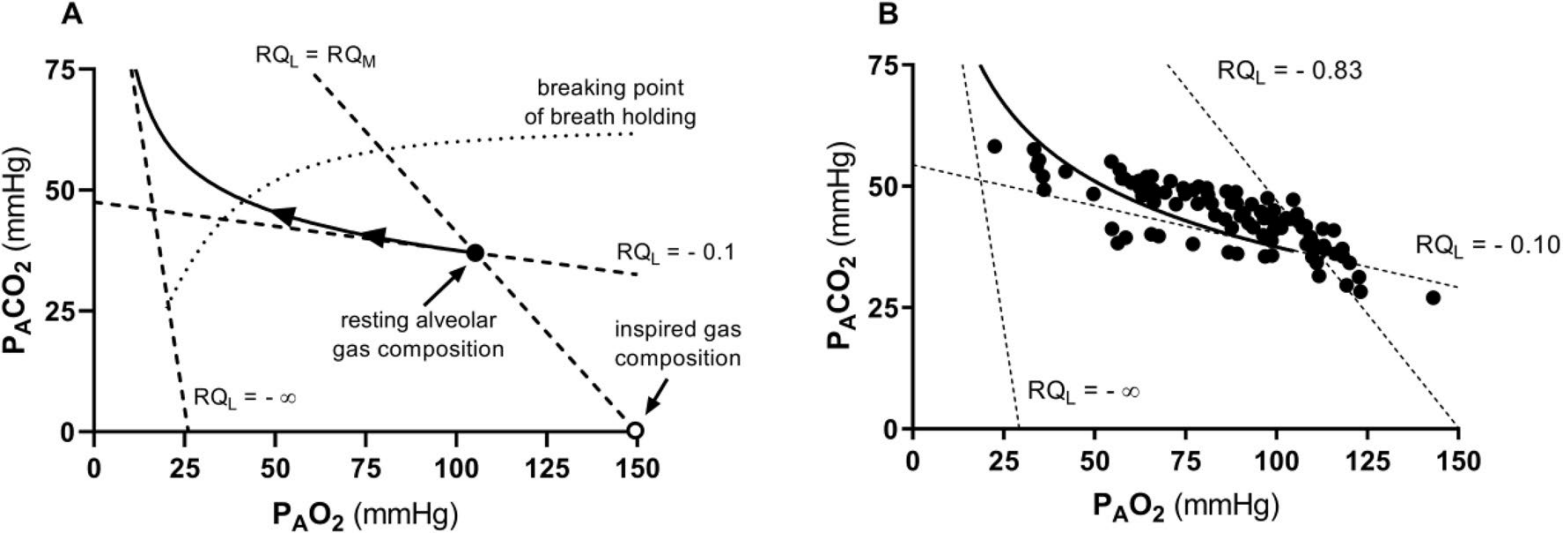
**A** Graphical representation of the hypoventilation loop model of the alveolar pathway during breath-holding (continuous curve) in air. Dashed lines represent the constructing lines, which are isopleths of the indicated lung respiratory quotient (${RQ}_{L}$). The dotted curve represents the conventional breaking point of breath-holding. Black arrowheads indicate the progressive changes of alveolar gas composition starting from the resting alveolar gas composition preceding breath-hold (black dot). ${RQ}_{M}$ metabolic respiratory quotient (at rest, − 0.83). **B** Experimental data of alveolar gas composition at the end of resting breath-holds of increasing duration up to the maximal, in air. The continuous curve represents the fitting function of the hypoventilation loop model. Dashed lines represent the constructing straight lines of the hypoventilation loop. ${P}_{A}{O}_{2}$ and ${P}_{A}{CO}_{2}$ alveolar partial pressures of oxygen and carbon dioxide, respectively [Modified after Taboni et al. (2020)]

Taboni et al. (2020) treated the alveolar gas pathways during breath-holding as an extreme hypoventilation loop. The theoretical loop is shown in Fig. 16a. The experimental data coincide with the model (Fig. 16b). The hypoventilation loop in ambient air is characterised by two segments. One is almost horizontal, depicting a large decrease in ${P}_{A}{O}_{2}$ accompanied by a minimal increase in ${P}_{A}{CO}_{2}$; the second segment bends upward, being characterised by a smaller decrease in ${P}_{A}{O}_{2}$ and a larger increase in ${P}_{A}{CO}_{2}$. The flat segment reflects the pressure gradient generated by the sudden fall of the ${\dot{V}}_{A}/{\dot{V}}_{R}{CO}_{2}$, which carries along the patterns of carbon dioxide storage in tissues (Farhi and Rahn 1960), while the oxygen stores are slowly emptied. The oxygen outflow from tissues does not correct for the oxygen transfer to lung capillaries in the absence of oxygen inflow from ambient air, whence the large fall of ${P}_{A}{O}_{2}$. This implies an extremely high ${RQ}_{L}$, totally dissociated from ${RQ}_{M}$, as reported in several studies (Ferretti et al. 1991; Lin et al. 1974; Otis et al. 1948; Taboni et al. 2020). In a few cases, a positive *RQ*_*L*_ was even found, suggesting reversal of carbon dioxide flux at the alveolar–capillary barrier (Ferretti et al. 1991; Lindholm and Linnarsson 2002). No correspondence between pulmonary gas exchange and tissue metabolism exists anymore.

As long as the pressure gradient sustaining it goes down, the gas flows between tissues and alveoli (or vice versa) decreases exponentially, tending toward a new equilibrium. As this occurs, the hypoventilation loop bends upward. As the equilibrium between alveoli and rapidly exchanging tissues has been attained, the ${P}_{A}{O}_{2}$ has become so low, that the pressure gradient sustaining oxygen flow across the alveolar–capillary barrier tends to zero. At the same time, the carbon dioxide produced by metabolism is entirely added to the alveoli, so that ${RQ}_{L}$ tends toward infinity, although the ${\dot{V}}_{A}/{\dot{V}}_{R}{CO}_{2}$ remains at 0. We attain the second trait of the hypoventilation loop.

In this condition, ${\dot{V}}_{R}{O}_{2}$ might still partly be sustained by changes in blood oxygen stores, through an increase in oxygen delivery associated with diaphragmatic contractions (Palada et al. 2008) and incurring arterial and venous haemoglobin desaturation (Lindholm et al. 1999, 2002), and by changes in muscle oxygen stores, which may be enhanced in divers (Elia et al. 2019). Blood and muscle oxygen stores may be improved by apnoea training (Elia et al. 2021a) and eventually by splenic contraction (Elia et al. 2021b).

Notwithstanding, this second part of the extreme hypoventilation loop cannot undergo experimental validation, so that it is deemed to remain a theoretical concept, because alveolar gas composition would go beyond that compatible with consciousness, if not with life. A maximal breath-hold is inevitably interrupted at an alveolar gas composition around that of the conventional breaking point curve (Lin et al. 1974). This allows predictions of the alveolar gas composition at the end of maximal breath-holds, independent of their duration, as it should correspond to the intersection of the experimental segment of the hypoventilation loop and the conventional breaking point curve, which also was modelled by fitting experimental data (Taboni et al. 2019).

During exercise apnoea, the patterns followed by alveolar gas composition are the same as at rest (Taboni et al. 2020). However, the time leading to the breaking point of apnoea is much shorter than at rest, because $\dot{E}$ is higher, as is $\dot{Q}$, so that the patterns of the cardiovascular responses to apnoeas are altered, and the rates of change of ${P}_{A}{O}_{2}$ and ${P}_{A}{CO}_{2}$ are increased (Lindholm et al. 2002; Sivieri et al. 2015; Taboni et al. 2018; Tocco et al. 2012) with respect to those demonstrated at rest (Costalat et al. 2013; Fagoni et al. 2015; Perini et al. 2008, 2010; Tocco et al. 2013).

During dives, alveolar gas composition is influenced also by changes in pressure and thus by the reduction of lung volume due to Boyle’s law (Ferretti 2001) and by complete pulmonary shunt at extreme depths (Fitz-Clarke 2007, 2009). The continuously changing external pressure during diving affects the dynamics of oxygen and carbon dioxide flows from and into the rapidly exchanging tissues, as compared with surface breath-holding (Linér and Linnarsson 1994). A hint of what may happen indeed during a dive has been provided by the analysis of ${P}_{a}{O}_{2}$ and ${P}_{a}{CO}_{2}$ during dives in simulated and real diving conditions (Bosco et al. 2020; Muth et al. 2003).

To sum up, breath-holding disrupts all equilibria in the respiratory system and no coupling of respiration to metabolism at all is possible. Whereas the cells try to cope with their energy demand, all regulations of the respiratory system are turned upside down. This being so, the only possibility of restoring the homeostatic equilibria is to interrupt apnoea. This occurs as soon as the conventional breaking point has been attained.

## Conclusions

**Fig. 17:**
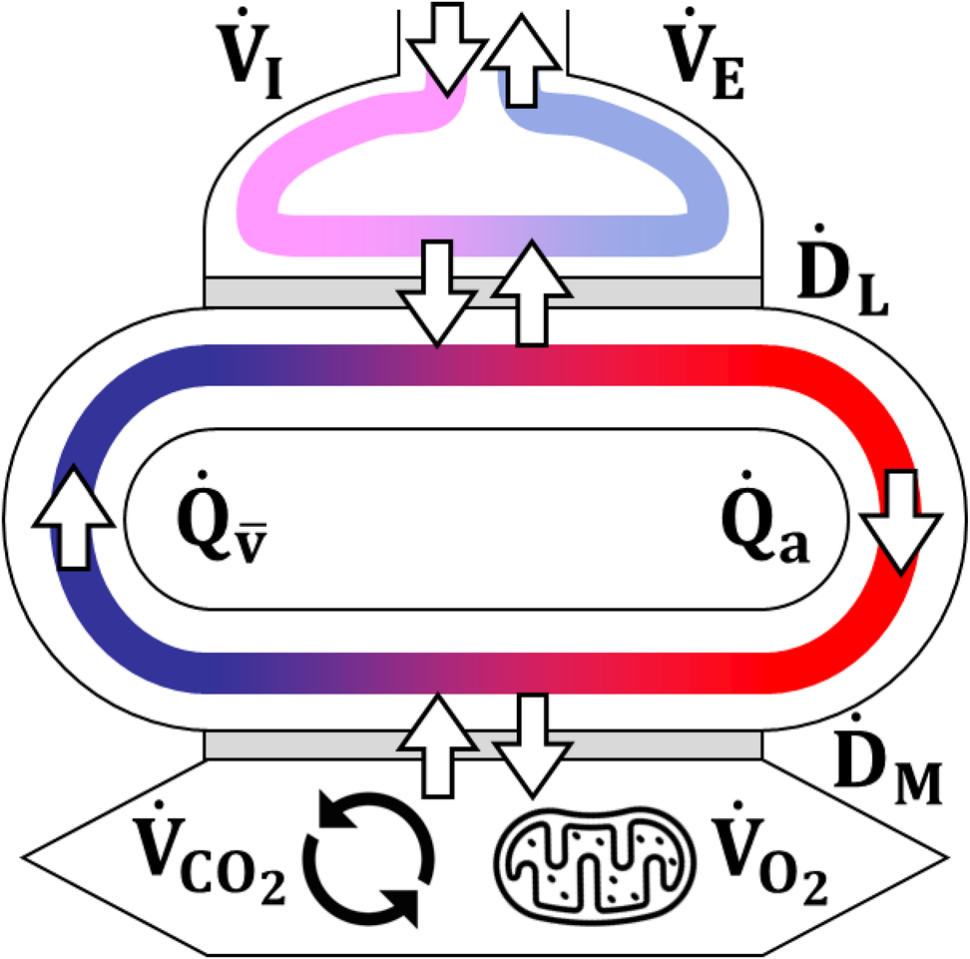
Schematic representation, in the form of a visual abstract, of the flow of oxygen and carbon dioxide along the respiratory system, from ambient air up to the actual site of chemical production (Krebs cycle) or consumption (mitochondria). Each white arrow represents a potential source of deviation from the steady state of the integrated flows. The oxygen or the carbon dioxide flows at a certain level along the system correspond to the difference between two opposite white arrows. ${\dot{V}}_{I}$ inspired gas flow, ${\dot{V}}_{E}$ expired gas flow, ${\dot{D}}_{L}$ lung diffusive flow of a gas, ${\dot{Q}}_{a}$ arterial flow of a gas, ${\dot{Q}}_{\overline{v} }$ venous flow of a gas, ${\dot{D}}_{M}$ muscular diffusive flow of a gas, $\dot{V}{O}_{2}$ oxygen consumption, $\dot{V}{CO}_{2}$ carbon dioxide production

In this review, we have outlined the conditions and constraints wherein a tight coupling of the respiratory system, meant in its holistic form (Taylor and Weibel 1981), and the cell (mostly muscle fibres during exercise) energy demand is attained. These conditions have been summarized in the form of a colour visual abstract in Fig. 17. The analysis of the steady-state condition has shown that the homeostatic equilibrium is centred on an arterial pH of 7.4 and a ${P}_{a}{CO}_{2} ={P}_{A}{CO}_{2}$ of 40 mmHg, with a ${\dot{V}}_{A}/{\dot{V}}_{R}{CO}_{2}$ of − 21.6. Several potential equilibria among the pertinent variables ensuring gas transfer along the respiratory system are compatible with the aforementioned homeostatic equilibrium, and they are actually exploited for instance at exercise, thus allowing adjustment of oxygen flow to oxygen demand.

These equilibria are broken in unsteady state. This may or may not break also the homeostatic equilibrium and the coupling of respiration and metabolism. This is not the case during the transient phase from rest to moderate exercise, when the response of the respiratory system is faster than that of the activation of the aerobic metabolic pathway. As the exercise intensity becomes higher, the response of the respiratory system gets slower and [*La*]_*e*_ appears: in this case, the coupling between respiration and metabolism is lost, but it is re-established as soon as, at steady state, lactate concentration stabilizes at a level that is higher than resting. However, if the increase in lactate concentration is such that the rapid blood buffering systems are unable to maintain arterial blood pH at 7.4, a slight hyperventilation, implying lower ${\dot{V}}_{A}/{\dot{V}}_{R}{CO}_{2}$ and ${P}_{a}{CO}_{2}$ below 40 mmHg.

In the severe exercise domain, above the critical power, the uncoupling cannot be recovered, anaerobic lactic metabolism provides energy beside and above the aerobic metabolism for the entire exercise duration, lactate concentration goes up, and arterial pH falls continuously, as does the pH of interstitial fluid. The absolute ${\dot{V}}_{A}/{\dot{V}}_{R}{CO}_{2}$ keeps decreasing below − 21.6, because of the ensuing hyperventilation, trying to restore pH; however, lactate keeps being accumulated in muscle and blood, at a rate that is higher, the higher is the exercise intensity. When this occurs, exhaustion is imminent.

The most catastrophic rupture of the homeostatic equilibrium occurs during breath-holding, because of the forced interruption of the oxygen flow from ambient air to mitochondria along the respiratory system, which is blocked and upset. No coupling at all is possible between respiration and metabolism in this case.

## Abbreviations

*A*: Surface area
*a*: Constant relating rate of phosphocreatine hydrolysis to rate of ATP resynthesis
ADP: Adenosine-di-phosphate
ATP: Adenosine-tri-phosphate
$\overleftarrow{\dot{ATP}}$: Rate of ATP hydrolysis
$\overrightarrow{\dot{ATP}}$: Rate of ATP resynthesis
*b*: Constant relating rate of lactate accumulation to rate of ATP resynthesis
BTPS: Body temperature and pressure, saturated with water vapour
*c*: Constant relating rate of oxygen consumption to rate of ATP resynthesis
${C}_{a}{CO}_{2}$: Carbon dioxide concentration in arterial blood
${C}_{a}{O}_{2}$: Oxygen concentration in arterial blood
*Cg*: Proportionality constant equal to the ratio between the STPD-to-BTPS and the ${P}_{A}{{\text{CO}}_{2}}$-to-${F}_{A}{{\text{CO}}_{2}}$ conversion factors
CO: Carbon monoxide
CO_2_: Carbon dioxide
${C}_{\overline{v}}{CO}_{2}$: Carbon dioxide concentration in mixed venous blood
${C}_{\overline{v}}{O}_{2}$: Oxygen concentration in mixed venous blood
*d*: Diffusion constant
${D}_{L}$: Lung diffusion capacity (conductance of the alveolar–capillary barrier)
$d\dot{M}$: Infinitesimal molar flow of a gas
${DO}_{2AL}$: Alactic oxygen deficit
${DO}_{2LA}$: Fraction of the oxygen deficit represented by early lactate
${DO}_{2M}$: Muscular oxygen deficit
*E*: Energy
$\dot{E}$: Overall metabolic power
${F}_{A}{CO}_{2}$: Carbon dioxide fraction in alveolar air
${F}_{E}{CO}_{2}$: Carbon dioxide fraction in expired air
${F}_{E}{O}_{2}$: Oxygen fraction in expired air
$${f}_{H}$$: Heart rate
${F}_{I}{CO}_{2}$: Carbon dioxide fraction in inspired air
${F}_{I}{N}_{2}$: Nitrogen fraction in inspired air
${F}_{I}{O}_{2}$: Oxygen fraction in inspired air
$\left[{Hb}\right]$: Blood haemoglobin concentration
$${K}_{e}$$: Equilibration coefficient
*L*: Length, thickness
$\dot{La}$: Rate of lactate accumulation in blood
[*La*]_*e*_: Early lactate, i.e., net blood lactate accumulation during the exercise transient
*n*: Number of moles (of gas in a volume or of oxygen bound to a mole of haemoglobin)
*O*_2_: Oxygen
*P*: Pressure
$\overline{P }$: Mean arterial pressure
*P*_50_: Oxygen partial pressure sustaining a saturation of 0.5
${P}_{A}{CO}_{2}$: Partial pressure of carbon dioxide in alveolar air
${P}_{a}{CO}_{2}$: Partial pressure of carbon dioxide in arterial blood
${P}_{A}{O}_{2}$: Oxygen partial pressures in alveolar air
${P}_{a}{O}_{2}$: Oxygen partial pressure in arterial blood
*P*_*B*_: Barometric pressure
${P}_{c}{O}_{2}$: Oxygen partial pressure in lung capillaries
${P}_{c^{\prime}}{O}_{2}$: Oxygen partial pressure at the end of lung capillaries
PCr: Phosphocreatine
$\dot{PCr}$: Rate of phosphocreatine decrease in contracting muscles
${P}_{c}$(*δ*): Capillary pressure at distance *δ* from the venous entrance
PFK: Phospho-fructo-kinase
pH: Negative logarithm of hydrogen ion concentration
Pi: Inorganic phosphate
${P}_{I}{O}_{2}$: Oxygen partial pressure in inspired air
*P/**La*: Moles of ATP resynthesized per mole of lactate accumulated
P-NMR: Phosphorus-nuclear magnetic resonance
${PO}_{2}$: Oxygen partial pressure, generic
*P*/*O*_*2*_: Moles of ATP resynthesized per mole of oxygen consumed
${P}_{\overline{v}}{O}_{2}$: Oxygen partial pressure in mixed venous blood
$\dot{Q}$: Total blood flow, or cardiac output
${\dot{Q}}_{a}{O}_{2}$: Oxygen flow in arterial blood, or oxygen delivery
${q}_{c}$: Lung capillary blood volume
${Q}_{s}$: Stroke volume of the heart
${\dot{Q}}_{\overline{v}}{O}_{2}$: Oxygen flow in mixed venous blood, or oxygen return
*R*: Energy introduced into 1 mol of gas by a unit increase in temperature
*r*: Rest (suffix)
*R*_*p*_: Total peripheral resistance
${RQ}_{B}$: Blood respiratory quotient
${RQ}_{L}$: Lung respiratory quotient
${RQ}_{M}$: Metabolic respiratory quotient
*RRi*: R–R interval, or time between two consecutive R peaks on an electrocardiogram
*s*: Solubility constant
${S}_{a}{O}_{2}$: Arterial oxygen saturation
*ss*: Steady state (suffix)
STPD: Standard temperature and pressure, dry
*T*: Temperature
*t*: Time
*T*_0_: Temperature at which *E* = 0 J
*t*½: Half-time
*t*_*c*_: Contact time
*V*: Volume
$$\dot{V}$$: Gas flow
$${\dot{V}}_{A}$$: Alveolar ventilation
${\dot{V}}_{A}/\dot{Q}$: Ventilation/perfusion ratio
${\dot{V}}_{A} /{\dot{V}}_{R}{\mathrm{CO}}_{2}$: Ratio between alveolar ventilation and lung carbon dioxide flow
$\dot{V}{CO}_{2}$: Rate of carbon dioxide production
${\dot{V}}_{E}$: Total expired air flow, or expiratory ventilation
${\dot{V}}_{I}$: Total inspired air flow, or inspiratory ventilation
${V}_{L}{O}_{2}$: Volume of oxygen crossing the alveolar–capillary barrier during a constant-load exercise
${\dot{V}}_{L}{O}_{2}$: Diffusive oxygen flow across the alveolar–capillary membrane
$\dot{V}{O}_{2}$: Rate of oxygen consumption
${VO}_{2M}$: Volume of oxygen extracted by the contracting muscles
${\dot{V}}{O}_{2}^{s}$: $\dot{V}{O}_{2}$ at steady state
$${\dot{V}}{O}_{2}^{t}$$: $$\dot{V}{O}_{2}$$ at time *t*
${\dot{V}}_{R}{CO}_{2}$: Carbon dioxide flow along the respiratory system
${\dot{V}}_{R}{O}_{2}$: Oxygen flow along the respiratory system
*V*_*v*_: Venous blood volume
*W*: Work
∆*G*: Free energy
∆[*La*]_*b*_: Net blood lactate accumulation
$$\Delta P$$: Pressure difference
∆*V*_*v*_*O*_2_: Changes in venous blood oxygen stores

## List of symbols

*β*: Blood transport coefficient of a gas, generic
*βc*: Blood transport coefficient of carbon dioxide
*βo*: Blood transport coefficient of oxygen
$\overline{\beta o }$: Mean slope of the oxygen equilibrium curve
*δ*: Distance from the venous entrance of a lung capillary
*λ*: Time constant increase per unit increase of lactate in blood during the exercise transient
*σ*: Oxygen-binding capacity of haemoglobin
*τ*: Time constant
*τ*_0_: Time constant in absence of early lactate accumulation

## Footnotes

1 This definition implies that in blood, ${\dot{V}}_{R}{O}_{2}$ and ${\dot{V}}_{R}{CO}_{2}$ are not the flow of the respective gases in arterial or in mixed venous blood, but the difference between these two flows; analogously, in the lungs, ${\dot{V}}_{R}{O}_{2}$ and ${\dot{V}}_{R}{CO}_{2}$ are not the flow of the respective gases in inspired or in expired air, but the difference between these two flows.

2 ${\dot{V}}_{R}{O}_{2}$ and ${\dot{V}}_{R}{CO}_{2}$ are flows along the respiratory system, oriented in opposite directions. Equations (﻿5a) and (﻿5b) clearly show that, since ${C}_{a}{O}_{2}$ > ${C}_{\stackrel{-}{v}}{O}_{2}$, whereas ${C}_{a}{CO}_{2}<{C}_{\overline{v}}{CO}_{2}$, ${\dot{V}}_{R}{O}_{2}$ results positive and ${\dot{V}}_{R}{CO}_{2}$ turns out negative. This implies that *RQ*_*L*_ is negative as well. This concept carries along a notation differing from the usual notation, wherein the modulus of these flows is used.

3 Since ${\dot{V}}_{R}{CO}_{2}$ is in STPD and ${\dot{V}}_{A}$ in BTPS, expressing both variables in standard condition, so that ${F}_{A}{CO}_{2} = \frac{{P}_{A}{CO}_{2} }{{P}_{B}{-47}}$, yields $\frac{{\dot{V}}_{A[{BTPS}]}}{{\dot{V}}_{R}{{CO}_{2}}_{[{STPD}]}}=-\frac{{P}_{B}-47 \, \mathrm{mmHg }}{{P}_{A}{CO}_{2}} \frac{760 \, \mathrm{mmHg}}{273 \, ^\circ \mathrm{K}}\frac{310 \, ^\circ \mathrm{K}}{{P}_{B}-47 \, \mathrm{mmHg}}=-\frac{863 \, \mathrm{mmHg }}{{P}_{A}{CO}_{2}}$. This means that, for ${P}_{A}{CO}_{2}=$ 40 mmHg, $\frac{{\dot{V}}_{A[{BTPS}]}}{{\dot{V}}_{R}{{\mathrm{CO}}_{2}}_{[{STPD}]}}=-\frac{863 }{40}=-21.6$. This value does not vary at exercise, because ${\dot{V}}_{A}$ and ${\dot{V}}_{R}{CO}_{2}$ increase in direct proportion.

4 The extraordinary stability of ${P}_{A}{CO}_{2}$ and ${P}_{a}{CO}_{2}$ around 40 mmHg throughout life has to do with the fact that the ventilatory response to carbon dioxide (actually to the *H*+ concentration in the cerebrospinal fluid) is a threshold phenomenon, with threshold at an *H*+ concentration compatible with ${P}_{a}{CO}_{2}=$ 40 mmHg. The phenomenon is a consequence of how the acid-sensing ion channels interact with *H*+ . Bound *H*+ is in dynamic equilibrium with free *H*+ . When a sufficiently high number of *H*+ units are bound to the extracellular region of the channel, the transmembrane domain 2 of the channel undergoes conformational changes leading to channel opening. An influx of sodium ions, and thus cell depolarization, occur, leading to activation of voltage-gated calcium channels. The ASIC 1 and ASIC 2 channels are widely expressed in the ventro-lateral medulla. For details, see e.g. Hanukoglu (2017), Song et al. (2016) and Zha (2013). On the role of central chemoreceptors in regulating pH and ${P}_{a}{CO}_{2}$, see Guyenet (2014), and Nattie and Li (2012).

5 It is important to mention that the arterial blood concentrations are weighed by perfusion to each unit, whereas the alveolar gas concentrations are weighed by the ventilation to each unit. The heterogeneity of perfusion distribution across the lungs is much more accentuated than the heterogeneity of ventilation distribution. Therefore, the number of alveolar units with low ${\dot{V}}_{A}/\dot{Q}$ values is by far larger than that of alveolar units with high ${\dot{V}}_{A}/\dot{Q}$ values, so that the former units contribute more than the latter to the formation of arterial blood.

6 In fact ${\dot{V}}_{A}=-\frac{\frac{760 }{{P}_{B}-47} \frac{310}{273} {RQ}_{L } \, {\dot{V}}_{R}{O}_{2}}{ \frac{{P}_{A}{CO}_{2}}{{P}_{B}-47}}$. Since the two terms (${P}_{B}-47$) cancel out, the proportionality constant in Eq. (﻿16) (*Cg*) turns out equal to 760*310/273, i.e., 863 mmHg if ${\dot{V}}_{R}{CO}_{2}$ is expressed in L min^−1^, or 0.863 mmHg if ${\dot{V}}_{R}{CO}_{2}$ is expressed in ml min^−1^.

7 Throughout the text, the acronym ${\dot{V}}_{R}{O}_{2}$ is used for oxygen flow, because it is the oxygen flow along the respiratory system. However, since these are steady-state relationships, wherein ${\dot{V}}_{R}{O}_{2}=\dot{V}{O}_{2}$, we could also use $\dot{V}{O}_{2}$ instead, as Cerretelli and di Prampero (1987) did.

8 It is fair to acknowledge that the piezo ion channel hypothesis on baroreceptors has been scrutinized and challenged by other authors (Stocker et al. 2019).

9 Equation (﻿35) does not include dissolved oxygen, which at a ${P}_{a}{O}_{2}$ of 100 mmHg contributes only 3 mL of oxygen per litre of blood, i.e., about 1.5% of the overall amount of oxygen in arterial blood: a negligible quantity indeed.

10 In this case, expressing $\left[{Hb}\right]$ in g L^−1^, ${\dot{V}}_{A}$ in L min^−1^, ${P}_{A}{CO}_{2}$ in mmHg and ${C}_{a}{O}_{2}-{C}_{\overline{v}}{O}_{2}$ in mL L^−1^, ${\dot{V}}_{R}{O}_{2}$ turns out in mL min^−1^.
